# A systematic review of the validity, reliability, and feasibility of measurement tools used to assess the physical activity and sedentary behaviour of pre-school aged children

**DOI:** 10.1186/s12966-021-01132-9

**Published:** 2021-11-04

**Authors:** Sophie M. Phillips, Carolyn Summerbell, Matthew Hobbs, Kathryn R. Hesketh, Sonia Saxena, Cassey Muir, Frances C. Hillier-Brown

**Affiliations:** 1 Department of Sport and Exercise Sciences, Durham University, Durham City, UK; 2 The Centre for Translational Research in Public Health (Fuse), Newcastle upon Tyne, UK; 3 School of Health Sciences, University of Canterbury, Christchurch, New Zealand; 4 Population Policy & Practice Research and Teaching Department, UCL Great Ormond Street Institute of Child Health, London, UK; 5 Faculty of Medicine, School of Public Health, Imperial College London, London, UK; 6 Population Health Sciences Institute, Newcastle University, Newcastle upon Tyne, UK; 7 Human Nutrition Research Centre , Newcastle University , Newcastle upon Tyne, UK; 8 Newcastle University Centre of Research Excellence in Healthier Lives Newcastle University , Newcastle upon Tyne, UK

**Keywords:** Physical activity, Sedentary behaviour, Pre-school, Validity, Reliability, Feasibility, Measurement

## Abstract

Physical activity (PA) and sedentary behaviour (SB) of pre-school aged children are associated with important health and developmental outcomes. Accurate measurement of these behaviours in young children is critical for research and practice in this area. The aim of this review was to examine the validity, reliability, and feasibility of measurement tools used to assess PA and SB of pre-school aged children.

Searches of electronic databases, and manual searching, were conducted to identify articles that examined the measurement properties (validity, reliability or feasibility) of measurement tools used to examine PA and/or SB of pre-school aged children (3–7 years old). Following screening, data were extracted and risk of bias assessment completed on all included articles.

A total of 69 articles, describing 75 individual studies were included. Studies assessed measurement tools for PA (*n* = 27), SB (*n* = 5), and both PA and SB (*n* = 43). Outcome measures of PA and SB differed between studies (e.g. moderate to vigorous activity, step count, posture allocation). Most studies examined the measurement properties of one measurement tool only (*n* = 65). Measurement tools examined included: calorimetry, direct observation, combined heart rate and accelerometry, heart rate monitors, accelerometers, pedometers, and proxy report (parent, carer or teacher reported) measures (questionnaires or diaries). Studies most frequently assessed the validity (criterion and convergent) (*n* = 65), face and content validity (*n* = 2), test-retest reliability (*n* = 10) and intra-instrument reliability (*n* = 1) of the measurement tools. Feasibility data was abstracted from 41 studies.

Multiple measurement tools used to measure PA and SB in pre-school aged children showed some degree of validity, reliability and feasibility, but often for different purposes. Accelerometers, including the Actigraph (in particular GT3X versions), Actical, ActivPAL and Fitbit (Flex and Zip), and proxy reported measurement tools used in combination may be useful for a range of outcome measures, to measure intensity alongside contextual information.

**Supplementary Information:**

The online version contains supplementary material available at 10.1186/s12966-021-01132-9.

## Background

Physical activity (PA) and sedentary behaviour (SB) in children are associated with numerous health and developmental outcomes [1–3]. Evidence of the importance of these associations in pre-school aged children has been a relatively recent area of research enquiry and is still emerging [4–7]. A pre-school aged child refers to any child who has not yet reached the age of formal schooling, usually aged between 3 and 5 years old but varies internationally [8]. The World Health Organization (WHO) [9] recommend that pre-school aged children should spend at least 180 minutes per day in a variety of physical activities, of which 60 minutes should include moderate to vigorous PA (MVPA). Recommendations for SB suggest that children should not be sedentary for extended periods of time, should not be restrained (such as in a pram) for more than 60 minutes at a time, and should engage in no more than 60 minutes of sedentary screen time per day. The WHO guidelines also suggest that when sedentary, pre-school aged children should engage in activities such as reading and storytelling [9]. Although estimates of guideline adherence vary in the literature, there is evidence to suggest that high proportions of pre-school aged children meet the 180 minutes PA guideline [10–12], but do not always engage in 60 minutes of MVPA [12]. Additionally, pre-school aged children are thought to spend extensive periods of their day sedentary [13, 14], and often do not meet the 60 minutes screen time guideline [10, 11, 13].

It is crucial to monitor PA and SB in pre-school aged children in response to changes in national and local policy; to survey guideline adherence; to develop appropriate policies and programmes; and to establish the efficacy of interventions and initiatives aimed at changing these behaviours [15, 16]. The measurement of PA and SB using quality tools which have optimal measurement properties, including validity, reliability and feasibility, are fundamental as they underpin research and practice in this area [15, 16]. However, there are no clear and up-to-date recommendations, or guidance, on the best tools to measure PA and SB in pre-school aged children.

PA and SB can be measured using various tools (or methods) including proxy report measures (questionnaires/diaries), device-based measurement tools (e.g. accelerometers, pedometers, heart rate monitors, combined heart rate and accelerometry), direct observation and measures of energy expenditure (e.g. doubly labelled water (DLW) and whole room calorimetry). Selecting the best quality tool to use for a particular purpose in any age group can be difficult [17] and there are additional and specific issues to consider for pre-school aged children. These include the more sporadic and intermittent nature of their movement [18], reduced cognitive capabilities which limit the ability to recall their own behaviour [19], and the increased likelihood that they will tamper with device based measurement tools [20].

Existing reviews have examined the measurement properties of selected measurement tools used to assess PA and SB in children [16, 21–25], including those which focused specifically on questionnaire based measures [26–29]. However, these reviews did not examine the full range of measurement tools used to assess PA and SB, and did not focus specifically on pre-school aged children.

In 2007, Oliver and colleagues, conducted a review examining prevalence and measurement issues in assessing the PA of pre-school aged children [19]. The authors summarised studies that had examined the validity and reliability of a range of measurement tools used to assess PA of young children; however, a rapid scoping search of relevant studies that we conducted prior to the review presented here, identified a large number of potential studies for inclusion that were published after Oliver et al’s review. Further, this review did not examine SB: the most likely reason for this is that the important associations of SB with health and developmental outcomes, independent of PA, have only started to emerge in the last 10–15 years [6, 7, 30].

Therefore, the aim of the present review was to examine the validity, reliability, and feasibility, of measurement tools used to assess PA and SB in pre-school aged children.

## Methods

This systematic review is reported in accordance with the Preferred Reporting Items for Systematic Reviews and Meta-Analyses (PRISMA) criteria [31] (see Additional file 1). The protocol for this systematic review was registered at the International Prospective Register for Systematic Reviews (PROSPERO), registration number CRD42019133613.

### Search strategy

Systematic searches of seven major electronic databases (Scopus, Web of Science, PsycARTICLES, PsycINFO, MEDLINE, CINAHL and SPORTDiscus); topic specific journals: Journal for the Measurement of Physical Behaviour and Pediatric Exercise Science; and the grey literature (opengrey.eu and Research Gate), were conducted to identify relevant studies. Searches were conducted in March 2019, and updated in March 2020. No restrictions were placed on language, year of study or publication status.

The search strategy included combinations of the: construct (physical activity, sedentary, sitting), population (pre-school, early years, early childhood, young children and kindergarten) and measurement properties (assessment, measurement, method, valid, reliable, feasible). Searches were adapted to each database, alongside the use of appropriate boolean operators and database specific filters (see Additional file 2). The lead review author (SMP) worked with the Durham University information science team, to refine the search strategies. References and citation searches of included studies, as well as checking the reference lists of selected existing reviews [16, 19, 21–29] were conducted for completeness.

### Eligibility criteria for included studies

Studies were eligible for inclusion if their aim was to examine the measurement properties (validity and/or reliability and/or feasibility) of a tool used to measure PA and/or SB of a general population sample of pre-school aged children between the ages of 3 and 7 years old. Table 1 provides an overview of the definitions of measurement properties that were examined in this review. There remains some debate over the terminology used for validity in the field of PA and SB measurement, particularly in relation to criterion validity [32]. For the purpose of this review, we developed a level of evidence scheme to distinguish between validity studies that used different comparison measurement tools. Level 1 includes criterion validity studies; the only methods considered ‘criterion’ were calorimetry based methods (e.g. whole room calorimetry and DLW) when compared against a measurement tool aiming to measure energy expenditure. Levels 2–4 include convergent validity, separated by the quality of the comparison measurement tool used. Level 2 includes studies where a tool has been compared against a measure which is considered to have relatively high validity (but not criterion), such as direct observation. Level 3 includes studies where a tool has been compared against a measure which is considered to have relatively low validity, such as device based measurement tools. Level 4 includes the comparison of two (or more) of the same type of measurement tool where neither tool is considered to have known higher validity, such as the outcomes of two makes of accelerometer being compared. Table 2 provides a full explanation on what constitutes each level of evidence.

**Table 1:** Definition of each of the measurement properties included in this review

Measurement property	Definition
**Validity**	*Ability for a measure to accurately reflect the construct it is designed to measure.*
Criterion validity	Output of a measure produces similar results to a ‘criterion’ measure. This includes studies that have examined a tool for determining energy expenditure with calorimetry (including doubly labelled water) used as the criterion measure.
Convergent validity	Output of a measure produces similar results to a reference measure not considered a criterion.
Face validity	Appearance of a measure, in that it appears to measure what it claims to measure
Content validity	Extent to which a measure covers all aspects of the intended domains or dimensions that it claims to measure
**Reliability**	*Extent to which a tool gives measurements that are consistent, stable and repeatable.*
Test-retest reliability	The extent to which a measure can obtain similar results in repeated trials, keeping as many conditions stable as possible
Inter/Intra Instrument Reliability	The extent to which scores are consistent when measurements are taken by different versions of the same instrument (inter-instrument) or by the same version of an instrument repeatedly (intra-instrument)
**Feasibility**	The extent to which a measurement tool: is suitable for the target population; can be successfully delivered in the target population/context; shows promise of being successful within the intended population. This can include: participant acceptability, researcher acceptability and cost, which can be assessed for all measurement tools through qualitative feedback of participants and through missing or lost data occurred from the measurement tool (with the exception of proxy or self-reported tools that can only be determined through qualitative means including the comprehensiveness and relevance of items).

Definitions guided by: Kelly et al. 2016 [32], Bowen et al. 2009 [33], Terwee et al. 2018 [34], Forouhi et al. [35], Evenson et al. [36]

**Table 2:** Level of evidence for validity studies included in this review

Level of evidence	Explanation	Criteria of comparisons (examples )	
		Measurement tool under study	Comparison tool/measure
1	Criterion validity	Any measurement tool to determine energy expenditure	Calorimetry, including doubly labelled water
2	Convergent validity: measurement tool compared with a measure which is considered to have *relatively high validity* (but not considered criterion)	Direct observation protocols (newly devised)	Direct observation protocol (pre-existing)
		Device based measurement tools: combined heart rate and accelerometer, heart rate monitor, accelerometer, pedometer	Direct observation Electrodiagram (for heart rate monitors)
		Proxy reported measurement tool: questionnaire, diaries	Direct observation
3	Convergent validity: measurement tool compared with a measure which is considered to have *relatively low validity*	Direct observation protocols (newly devised)	Device based measurement tools: combined heart rate and accelerometer, heart rate monitor, accelerometer or pedometer
		Device based measurement tools: combined heart rate and accelerometer, heart rate monitor, accelerometer, pedometer	Device based measurement tools with known higher validity than tool under study: combined heart rate and accelerometer, heart rate monitor, accelerometer
		Proxy reported measurement tool: questionnaire, diaries	Device based measurement tools: combined heart rate and accelerometer, heart rate monitor, accelerometer or pedometer
4	Convergent validity: two (or more) of the same type of measurement tools being compared, where *neither tool is considered to have known higher validity*	Device based measurement tools: combined heart rate and accelerometer, heart rate monitor, accelerometer	Device based measurement tools: combined heart rate and accelerometer, heart rate monitor, accelerometer
		Proxy reported measurement tool: questionnaire, diaries	Proxy reported measurement tool: questionnaire, diaries

Studies were excluded if:

- The aim of the study *was not* to examine the measurement properties of the tool. For example, studies were excluded if the aim was to examine the reproducibility or tracking of behaviours over time, inter-observer reliability, epoch lengths, wear time, the calibration of cut points or prediction equations (with no separate validation study);
- The aim of the study was *not* to examine a tool measuring PA and/or SB. For example, studies were excluded if their aim was to examine physical fitness, motor skills, PA environment, correlates of PA or SB, or the impact of interventions;
- The study included children under 3 years or over 7 years of age, or if the population sample included children with chronic conditions;
- The study was a study protocol or a review. Higher degree theses and conference abstracts were included, however, where the relevant information from the theses were also provided in published peer-reviewed journal articles, the journal article was included and the thesis excluded.

### Screening for relevant studies to include in the review

Following the searches, all identified articles were imported into a referencing manager software (Endnote X9.1) and duplicates were removed. Titles and abstracts of included articles were screened by the lead review author (SMP) for inclusion, with a further 10% screened by a second reviewer (MH). There was very high agreement between the two reviewers for title and abstract screening, with a Cohen’s kappa statistic [37] of *k* = 0.94. Any disagreement on the eligibility of particular studies was resolved through discussion, without the need for escalation to a third reviewer (FCHB). Full texts of potentially relevant studies were then double screened by two reviewers (SMP, MH, and/or FCHB) for inclusion.

### Data extraction of individual studies included in the review

Data from all relevant studies were extracted independently by two data extractors (SMP, CM, and/or FCHB) using a pre-piloted data extraction form. Any discrepancies were resolved by discussion. Extracted information included: study characteristics, participant characteristics, the measurement tool explored (e.g. accelerometry), the measurement tool(s) used as a comparison, data interpretation choices for device based measurement tools (e.g. cut points, epoch, placement), statistical method used to compare measurement tools, behaviour category (PA and/or SB), and the details of the units of measure (e.g. moderate-to-vigorous PA), measurement properties assessed (e.g. criterion validity), the results of the study, and the sources of funding for the study.

### Risk of bias assessment of individual studies included in the review

Risk of bias assessment was conducted independently by two reviewers (SMP, FCHB), with any discrepancies resolved through discussion. The risk of bias of all individual studies included in this review was assessed using a modified version of the Downs and Black Checklist, a method suitable for appraising non-randomised studies [38]. This modified checklist has been successfully used in previous systematic reviews examining PA assessment measures [21, 39]. The tool includes seventeen questions: eight focus on the quality of reported criteria, three on the external validity and five the internal validity. The maximum quality score a study could receive was 17, with study quality rated as good (13-17), fair (9-< 13) or poor (< 9), based on the protocols used in previous reviews [40–42].

An additional risk of bias assessment was conducted on studies examining proxy reported measurement tools, using the COnsensus-based Standards for the selection of health Measurement INstruments (COSMIN) risk of bias checklist [43, 44]. This checklist was devised specifically for assessing the risk of bias of participant reported measurement property studies. Based on the studies included in our review we conducted the assessment using the sub-sections relating to reliability and construct (convergent) validity. It was not possible to assess the quality of the content validity of the studies due to such minimal information available. Each item was scored using a 4 point scale (very good, adequate, doubtful, inadequate). The overall methodological rating of a study was determined using the COSMIN protocol of ‘the worst score counts’ principle [45] (e.g. if the lowest rating of all items was ‘doubtful’, the overall methodological quality of the measurement property in that study would be rated as ‘doubtful’).

### Interpretation of validity, reliability, and feasibility

Studies commonly use a number of different statistical analyses to define absolute (agreement between the two measurement tools) or relative (the degree to which the two measurement tools rank individuals in the same order) validity and reliability [32, 35]. These types of statistical analyses include correlations (Pearson’s; Spearman’s; Kendall’s; Intraclass), linear regressions, root mean square error (RMSE), Bland Altman, kappa statistics and area under the receiver operating curve (AUC-ROC) [35, 46–51]. Additionally, studies use different methods of analysing and reporting the feasibility of measurement tools. In order to demonstrate consistency in the interpretation of the results across studies, and also to compare and rank these results, we scoped the relevant literature to search for guidance.

We found no consensus in the literature as to which statistical test results indicate weak, moderate, or good validity or reliability. However, in line with a number of previous reviews of this type [23, 26, 28, 29, 36, 52], we provide an overview of what constitutes a ‘weak’, ‘moderate’, or ‘good’ statistical result for validity or reliability, to rank individual studies in this way.

For proxy reported based measurement tools, feasibility can only be determined using qualitative methods, including to determine if questions are relevant, comprehensible and understandable [34]. However, there is no standardised way of determining feasibility of other measurement tools. Therefore, feasibility in the present review was based on qualitative acceptability or feasibility of the measurement tools where data was available. Additionally, an indication of feasibility was also based on compliance and usable data information using values from the Effective Public Health Practice Project (EPHPP) quality assessment tool for withdrawal and drop out [53]. The EPHPP is primarily used for assessing the quality of quantitative intervention based studies in systematic reviews and rates studies as ‘strong’, ‘moderate’, or ‘weak’ based on the percentage of participants completing the study. We applied the criteria to provide an indication on the feasibility of the measurement tools in our review, to indicate a ‘weak’, ‘moderate’, or ‘good’ level of feasibility based on the percentage of usable data from the measurement tools (as a result of missing data and/or drop out) [53]. This was used to provide an indication on feasibility only, as the true feasibility of measurement tools should be determined through the use of qualitative research methods [36, 54–56]. When interpreting summary scores for feasibility, more weight was given to qualitative findings; the scores of these studies were based on the qualitative data provided in the original study. Information on the interpretation of the studies can be found in Table 3.

**Table 3:** Main statistical analyses and interpretation of statistics

	Relative or Absolute Validity/reliability?	Weak	Moderate	Good
**Correlations (*r*)**				
**Pearson’s**	Relative	< 0.60	0.60–0.79	≥0.80
**Spearman’s**	Relative	< 0.60	0.60–0.79	≥0.80
**Kendall’s**	Relative	< 0.60	0.60–0.79	≥0.80
**Intraclass correlation coefficient**	Absolute	< 0.60	0.60–0.69	≥0.70
**Linear Regression** (% variance explained by the measurement tool)	Relative	< 60%	60–79%	≥80%
**Root mean squared error**	Absolute	*	*	*
**Bland Altman (mean difference, limits of agreement, bias)**	Absolute	*	*	*
**Kappa** (*r*)	Absolute	< 0.60	0.60–0.69	≥0.70
**Area under the receiver operating curve (AUC-ROC)**	Relative	< 0.70	0.70–0.79	≥0.80
**Feasibility (% of usable data)**		< 60%	60–79%	80–100%

(References: ([23, 26, 28, 29, 35, 36, 46–53]))

*Depends on the units of measurement

### Combining the results of individual studies for overall interpretation

We summarised the results of studies where they aimed to compare a particular measurement property (separated by level of evidence for validity) of a particular measurement tool (e.g. Actigraph GT3X accelerometers). We have included summaries of this information in: Table 4 (level 1 validity evidence), Table 5 (level 2 validity evidence), Table 6 (level 3 validity evidence), Table 7 (level 4 validity evidence), and Table 8 (reliability and feasibility evidence). The evidence outlined in the tables was interpreted as follows:

- Where the specific measurement property for a specific measurement tool was deemed ‘good’ in over half of these studies, the summary assessment was deemed to be ‘good’.
- Where the specific measurement property for a specific measurement tool was deemed ‘moderate’ in over half of these studies, the overall assessment was deemed to be ‘moderate’.
- Where the specific measurement property for a specific measurement tool was deemed ‘weak’ in over half of these studies, the overall assessment was deemed to be ‘weak’.
- In instances where the specific measurement property of a measurement tool had mixed evidence in the studies, such as studies with outcomes of ‘weak’ and ‘moderate’, or ‘moderate’ and ‘good’, the overall assessment was deemed to be the most positive of the two outcomes.

**Table 4:** Summary table of level 1 validity evidence of the measurement tools

Measurement tools under study	Outcome measures		References
	Energy expenditure	VO_**2**_	
**Combined heart rate and accelerometer**			
Actiheart			[57]
**Accelerometers**			
Actigraph (MTI)			[58, 59]
Actigraph (GT3X)			[60, 61]
Actical			[57, 62, 63]
Actigraph (wGT3X-BT)			[64]
ActivPAL			[60, 65]
GENEActiv			[60]
Triaxial Research Tracker 3 (RT3)			[57]
Actiwatch (AW16)			[66]
Tracmor_D_			[67]
**Proxy reported measurement tools**			
Children’s physical activity questionnaire (CPAQ)			[68]

This table shows a summary of the results of studies where they aimed to compare a particular measurement tool (e.g. Actigraph GT3X accelerometer) against calorimetry (including doubly labelled water). The summary ratings were based on the quality of the tools for this specific measurement property. Where the measurement tool was deemed ‘good’ in the majority of the studies, the summary assessment was deemed ‘good’. Where the measurement tool was deemed ‘moderate’ in the majority of the studies, the summary assessment was deemed ‘moderate’. Where the measurement tool was deemed ‘weak’ in the majority of the studies, the summary assessment was deemed ‘weak’. In instances where the measurement tool had mixed evidence in the studies, such as studies with outcomes of ‘weak’ and ‘moderate’, or ‘moderate’ and ‘good’, the overall assessment was deemed to be the most positive of the two outcomes. All tools of reasonable quality where any evidence was available are included in this table, including where only one or two studies reported that result.

Cut points: ^1^Ekelund et al. 2001 [69]; ^2^Puyau et al. 2002 [70]; ^3^Pate et al. 2006 [58]; ^4^ Evenson et al. 2008 [71]; ^5^Pfeiffer et al. 2006 [63]; ^6^Adolph et al. 2012 [57]

*Methodology used to assess the ability of the tool is detailed in the methods above and is indicated in the summary table as:

Good =  Moderate=  Weak=

Key for colour of boxes:

= evidence from ≥3 studies

= evidence from <3 studies

**Table 5:** Summary table of level 2 validity evidence of the measurement tools

Method	Outcome measures							References
	SB	Posture allocation	LPA	MVPA	TPA	Levels of activity	Step counts	
**Direct observation**								
Fargo Activity Timesampling Survey (FATS-continuous sampling)								[72]
**Combined heart rate and accelerometer**								
Actiheart								[57]
**Heart rate monitor (HRM)**								
Polar Vantage XL Monitor								[73]
**Accelerometers**								
Actigraph (GT3X+)								[74]
Actigraph (GT3X)								[61, 75–77]
Actigraph (GT1M)								[78–80]
Actigraph (MTI/CSA)								[81–85]
Actical								[57, 62, 86]
ActivPAL								[65, 78, 87–89]
Fitbit (Flex)								[90]
Fitbit (Zip)								[91]
NewLifestyles NL-1000								[92]
Triaxial Research Tracker 3 (RT3)								[57, 80]
Actiwatch (Spectrum)								[76]
Actiwatch (MiniMitter)								[93]
Actiwatch (AW16)								[83]
Actiwatch L								[94]
**Pedometers**								
Yamax Digiwalker (SW-200)								[82, 95–97]
Yamasa AM-5 Pedometer								[98]
MVP 4 Walk4Life Digital								[99]
**Proxy reported measurement tools**								
Teacher/mother reported habitual PA								[98, 100]

Published cut points used:^1^ *Evenson et al., 2008* [71]; ^2^ *Johansson et al., 2015* [101]; ^*3*^*Pate et al., 2006* [58]; ^4^ *Sirard et al., 2005 *[85]; ^5^*Van Cauwenberghe et al., 2011 * [102]; ^*6*^*Puyau et al., 2002 *[70]; ^*7*^*Reilly et al., 2003 *[84]; ^*8*^*Freedson et al., 2005* [103]; ^*9*^*Adolph et al., 2012* [57]; ^10^ *Pfeiffer et al., 2006* [63]; ^*11*^ *Schaefer et al., 2014* [104]; ^12^*Vanhelst et al., 2000* [105]; ^13^*Rowlands et al.,2004* [106]; ^14^*Sun et al., 2008* [107]; ^15^*Chu et al.,2007* [108]; ^16^*Ekblom et al., 2012* [109]

*Methodology used to assess the ability of the tool is detailed in the methods above and is indicated in the summary table as:

Good =  Moderate=  Weak=

Key for colour of boxes: = evidence from ≥3 studies

= evidence from <3 studies

This table shows a summary of the results of studies where they aimed to compare a particular measurement tool (e.g. Actigraph GT3X accelerometer) against direct observation (or electrodiagram for the heart rate monitor). The summary ratings were based on the quality of the tools for this specific measurement property. Where the measurement tool was deemed ‘good’ in the majority of the studies, the summary assessment was deemed ‘good’. Where the measurement tool was deemed ‘moderate’ in the majority of the studies, the summary assessment was deemed ‘moderate’. Where the measurement tool was deemed ‘weak’ in the majority of the studies, the summary assessment was deemed ‘weak’. In instances where the measurement tool had mixed evidence in the studies, such as studies with outcomes of ‘weak’ and ‘moderate’, or ‘moderate’ and ‘good’, the overall assessment was deemed to be the most positive of the two outcomes. All tools of reasonable quality where any evidence was available are included in this table, including where only one or two studies reported that result

**Table 6:** Summary table of level 3 validity evidence of the measurement tools

**Measurement tools under study**	**Outcome measures**							**References**
	**SB**	**Posture allocation**	**LPA**	**MVPA**	**TPA**	**Levels of activity**	**Step count**	
**Direct observation**								
OSRAC-P (Observation System for Recording Physical Activity in Children- Preschool)								[110]
SOFIT-P (System for Observing Fitness Instruction Time for Preschoolers)								[111]
**Accelerometers**								
Fitbit (Flex)								[112]
ActivPAL								[113]
Best Fit Friend								[114]
**Pedometers**								
Yamax Digi-Walker (SW-200)								[115, 116]
Yamax Digi-Walker (SW-700)								[117]
Omron Walking Style Pro (HJ-720IT-E2)								[118, 119]
**Proxy reported measurement tools**								
Nursery teacher’s report *(based on Toyama Cohort Study survey questions)*								[120]
Leisure time report								[121]
Pre School Physical Activity Questionnaire (PRE-PAQ)								[122]
Netherland’s Physical Activity Questionnaire (NPAQ)								[123]
Questionnaire developed for parents of pre-schoolers in Mexico								[124]
Teacher activity rating								[121]
7 day activity diary (adapted from CLASS)								[125]
Habitual Activity Estimation Scale (HAES)								[126]
Children’s physical activity questionnaire (CPAQ)								[68]
Children’s Leisure Activities Study Survey (CLASS)								[127]
TV Diary								[128]
Teacher/mother reported habitual PA								[86, 98]

This table shows a summary of the results of studies where they aimed to compare a particular measurement tool (e.g. Pre School Physical Activity Questionnaire (PRE-PAQ)) against a measure which is considered to have *relatively low validity* such as a device based measurement tool (see Table 2 for a full explanation)). The summary ratings were based on the quality of the tools for this specific measurement property. Where the measurement tool was deemed ‘good’ in the majority of the studies, the summary assessment was deemed ‘good’. Where the measurement tool was deemed ‘moderate’ in the majority of the studies, the summary assessment was deemed ‘moderate’. Where the measurement tool was deemed ‘weak’ in the majority of the studies, the summary assessment was deemed ‘weak’. In instances where the measurement tool had mixed evidence in the studies, such as studies with outcomes of ‘weak’ and ‘moderate’, or ‘moderate’ and ‘good’, the overall assessment was deemed to be the most positive of the two outcomes. All tools of reasonable quality where any evidence was available are included in this table, including where only one or two studies reported that result.

*Methodology used to assess the ability of the tool is detailed in the methods above and is indicated in the summary table as:

Good =  Moderate=  Weak=

Key for colour of boxes:

= evidence from ≥3 studies

= evidence from <3 studies

**Table 7:** Summary table of level 4 validity evidence of the measurement tools

Measurement tools under study	Outcome measures					Reference
	SB	MVPA	TPA	Activity counts	Habitual physical activity reports	
**Combined heart rate and accelerometer and accelerometers**						
Actiheart, Actical and Triaxial Research Tracker 3 (RT3)						[57]
**Accelerometers**						
Actigraph (GT1M)^1-6^ and RT3^7-10^						[80]
Actigraph (GT1M)^4^ and ActivPAL						[78]
Actigraph (GT3X+)^3^ and Actical^11^						[129]
Actigraph (GT3X)^1,3^ and Actiwatch (Spectrum)^12^						[76]
Actigraph (CSA/MTI) and Actiwatch (AW16)						[83]
Actical^4^ and ActivPAL						[130]
**Proxy reported measurement tools**						
Parental vs teacher reports						[98, 100]

This table shows a summary of the results of studies where they aimed to compare two (or more) of the same type of measurement tools where *neither tool is considered to have known higher validity* (e.g. comparison between Actical and ActivPAL). The summary ratings were based on the quality of the tools for this specific measurement property. Where the measurement tool was deemed ‘good’ in the majority of the studies, the summary assessment was deemed ‘good’. Where the measurement tool was deemed ‘moderate’ in the majority of the studies, the summary assessment was deemed ‘moderate’. Where the measurement tool was deemed ‘weak’ in the majority of the studies, the summary assessment was deemed ‘weak’. In instances where the measurement tool had mixed evidence in the studies, such as studies with outcomes of ‘weak’ and ‘moderate’, or ‘moderate’ and ‘good’, the overall assessment was deemed to be the most positive of the two outcomes. All tools of reasonable quality where any evidence was available are included in this table, including where only one or two studies reported that result.

Cut points: ^1^Sirard et al. 2005 [85]; ^2^Freedson et al. 2005 [103]; ^3^Pate et al., 2006 [58]; ^4^Evenson et al. 2008 [71]; ^5^Van Cauwenberghe et al. 2011 [102]; ^6^Puyau et al. 2002 [70]; ^7^Vanhelst et al. 2000 [105]; ^8^Rowlands et al. 2004 [106]; ^9^Sun et al. 2008 [107]; ^10^Chu et al. 2007 [108]; ^11^Pfeiffer et al. 2006 [63]; ^12^Ekblom et al. 2012 [109]

*Methodology used to assess the ability of the tool is detailed in the methods above and is indicated in the summary table as:

Good =  Moderate=  Weak=

Key for colour of boxes:

= evidence from ≥3 studies

= evidence from <3 studies

**Table 8:** Summary table of the reliability and feasibility of the measurement tools

Method	Reliability	Feasibility	Reference
**Calorimetry**			[60–62, 65–67, 131, 132]
**Direct observation**			[72, 76, 87, 91, 111, 133]
**Heart rate monitors**			
Polar Vantage XL Monitor			[73]
**Accelerometers**			
Fitbit (Zip)			[91]
Triaxial Research Tracker 3 (RT3)			[117]
Actigraph (GT3X/+)			[75, 76, 113, 114, 129]
Actigraph (CSA/MTI)			[68, 82, 83, 85, 115, 122]
Actigraph (wGT3X-BT)			[64]
Actigraph (GT1M)			[78, 113, 116, 118, 124, 125, 128]
ActivPAL			[65, 78, 87–89, 113, 130]
Actical			[86, 129, 130]
Actiwatch (AW16)			[66, 83]
Actiwatch (Spectrum)			[76]
Actiwatch-L			[120]
Caloriecounter			[120]
Tracmor_D_			[67]
Caltrac			[134]
Best Fit Friend			[114]
**Pedometers**			
Yamax (SW-700)			[117, 126]
Yamax Digiwalker (SW-200)			[95, 115, 116]
MVP 4 Walk4Life Digital			[99]
Omron Walking Style Pro (HJ-720IT-E2)			[118]
**Proxy reported measurement tools**			
Children’s Leisure Activities Study Survey (CLASS)			[127]
Questionnaire developed for parents of pre-schoolers in Mexico			[124]
Teacher activity rating			[121]
TV Diary			[128]
Leisure time report			[121]
Pre School Physical Activity Questionnaire (PRE-PAQ)			[122]
Netherland’s Physical Activity Questionnaire (NPAQ)			[123]
‘Toybox’ Primary Caregivers Questionnaire			[135]
Children’s physical activity questionnaire (CPAQ)			[68]

*Methodology used to assess the ability of the tool is detailed in the methods above and is indicated in the summary table as:

Good =  Moderate=  Weak=

Key for colour of boxes: = evidence from ≥3 studies

= evidence from <3 studies

This table shows a summary of the results of studies where they tested the reliability or feasibility of the measurement tool. The summary ratings were based on the quality of the tools for the specific measurement property. Where the measurement tool was deemed ‘good’ in the majority of the studies, the summary assessment was deemed ‘good’. Where the measurement tool was deemed ‘moderate’ in the majority of the studies, the summary assessment was deemed ‘moderate’. Where the measurement tool was deemed ‘weak’ in the majority of the studies, the summary assessment was deemed ‘weak’. In instances where the measurement tool had mixed evidence in the studies, such as studies with outcomes of ‘weak’ and ‘moderate’, or ‘moderate’ and ‘good’, the overall assessment was deemed to be the most positive of the two outcomes. All tools of reasonable quality where any evidence was available are included in this table, including where only one or two studies reported that result

For completeness, we have included information about all tools of reasonable quality in the summary tables (Tables 4, 5, 6, 7 and 8) where there was any available evidence, including where there was only one or two studies that reported results. The information is greyed out to highlight where the evidence is based on ≥3 studies.

## Results

### Study selection

**Fig. 1:**
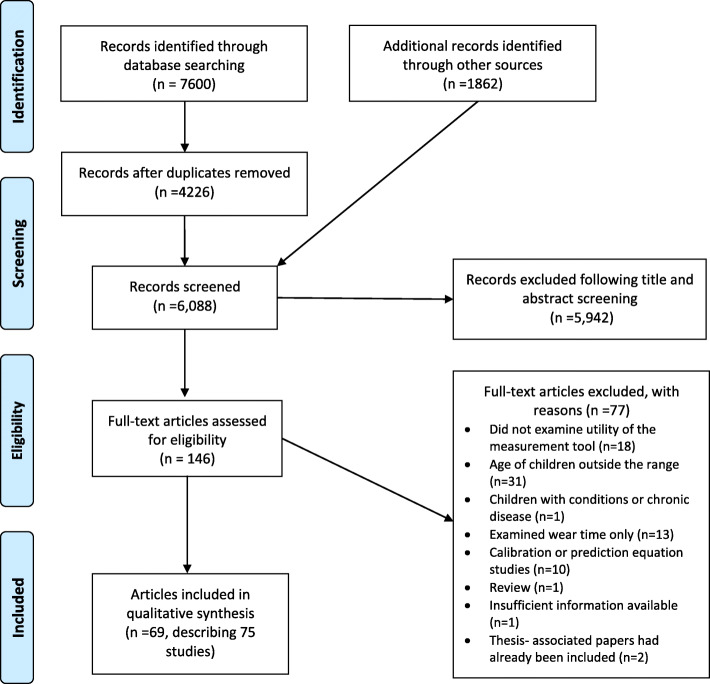
Flow chart of included studies [31]

A total of 6088 records were screened for inclusion, of which 146 were included for full text screening and 69 articles were included in the review, describing 75 individual studies (See Fig. 1). Sixty seven articles were retrieved following the initial searches, and a further two from the updated searches [60, 75]. All included articles were in English and, in most cases, were peer reviewed journal articles (*n* = 66) [57–68, 72–98, 100, 110–113, 115–118, 120–137]. We included one abstract [119] and two Masters theses [99, 114] due to the articles meeting the inclusion criteria and providing sufficient information. A list of excluded studies with reasons can be found in Additional file 3.

### Description of studies

Detailed information of all included studies can be found in Additional files: 4 (level 1 validity evidence), 5 (level 2 validity evidence), 6 (level 3 validity evidence), 7 (level 4 validity evidence), 8 (reliability) and 9 (feasibility). The majority of the studies were conducted in high income countries: USA (*n* = 24), UK (*n* = 17), Australia (*n* = 10), Belgium (*n* = 3), Canada (*n* = 3), Hong Kong (*n* = 3), New Zealand (*n* = 3), Greece (*n* = 2), Japan (*n* = 2), Netherlands (*n* = 2), Germany (*n* = 1), Korea (*n* = 1), Sweden (*n* = 1), and both the USA and Sweden (*n* = 1). One study was conducted in Mexico, an upper to middle income country [124] and one study was a collaboration between high income and an upper to middle income country: Belgium, Bulgaria, Germany, Greece, Poland and Spain [135]. No studies were conducted in low income countries. Study sample sizes ranged from 4 [110] to 269 [85] (median *n* = 34). Based on criteria outlined in the COSMIN measurement property checklist [43], only 7 of the included studies had excellent (*n* = > 100), 15 had good (*n* = 50–99), 26 had moderate (*n* = 30–49), and 27 had small (*n* = < 30) sample sizes. All studies reporting child sex (*n* = 69) included both male and female participants. The median age of children included in the studies was 4.5 years. The studies commonly explored the measurement properties of the measurement tools in free-living conditions (*n* = 54), used structured protocols that were reflective of habitual behaviour of pre-school aged children (*n* = 11) or were conducted in laboratory settings (*n* = 10). A large proportion of the studies did not report on any funding received for the research (*n* = 31). Many of the studies that reported funding were supported by more than one funding source (see Additional file 10 for funding sources of studies).

Studies assessed the measurement tools for PA only (*n* = 27), SB only (*n* = 5), and both PA and SB (*n* = 43). Units of measure varied across studies, and included: activity preferences, activity levels, activity counts, activity energy expenditure, energy expenditure, frequency of activity, heart rate, metabolic equivalent of task (MET) values, posture allocation, step count, time spent in different intensities of activity and VO_2_.

The majority of the studies examined the measurement properties of one measurement tool only (*n* = 65). Several studies examined the measurement properties of two (*n* = 8) or infrequently three (*n* = 2) measurement tools simultaneously, in comparison with the reference methods. Articles examined the measurement properties of: calorimetry (*n* = 2), direct observation (*n* = 4), combined heart rate and accelerometry (*n* = 1), heart rate monitors (*n* = 1), accelerometers (*n* = 44), pedometers (*n* = 13), and proxy report measures (questionnaires or diaries) reported by parent, carer or teacher (*n* = 13).

Validity of the measurement tools was the most frequently examined measurement property; level 1 validity (*n* = 12), level 2 validity (*n* = 36), level 3 validity (*n* = 23) and level 4 validity (*n* = 9). Only two studies examined the face and content validity of the measurement tools. Ten studies described the test-retest reliability of the measurement tools and 1 study the intra-instrument reliability. Feasibility data was abstracted from 41 studies; 13 of these studies had a primary aim of determining the feasibility of the measurement tool, the remaining 28 studies commented on reasons for drop out or exclusion of data, which also classified as assessing feasibility. Table 9 provides an overview of the measurement properties that were examined for each tool, to help determine which type of evaluation was conducted on each of the measurement tools. This table does not state the quality of the tools; please refer to Tables 4, 5, 6, 7 and 8 that outline summaries of the quality of the tools based on the available evidence.

**Table 9:** Overview of all measurement tools examined and measurement properties explored

Measurement tools examined	Validity^a^					Reliability		Feasibility
	Criterion: Level 1	Convergent: Level 2	Convergent: Level 3	Convergent: Level 4	Face/Content	Test-retest-	Intra-instrument	
**Calorimetry**								•
**Direct observation**		•	•					•
**Combined heart rate and accelerometer**								
Actiheart	•	•		•				
**Heart rate monitor**								
Polar Vantage XL Monitor		•						•
**Accelerometers**								
Actical	•	•		•				•
Actigraph (CSA/MTI)	•	•		•				•
Actigraph (GT1M)		•		•				•
Actigraph (GT3X)	•	•		•				•
Actigraph (GT3X+)		•		•				•
Actigraph (wGT3X-BT)	•							•
Actometer		•						
ActivPAL	•	•	•	•				•
Actiwatch (AW16)	•	•		•				•
Actiwatch (MiniMitter)		•						
Actiwatch (Spectrum)		•		•				•
Actiwatch-L		•						•
Best Fit Friend			•					•
Caloriecounter								•
Caltrac								•
Fitbit (Flex)		•	•					
Fitbit (Zip)		•					•	•
GENEActiv	•							
New Lifestyles NL-1000		•						
Tracmor_D_	•							•
Triaxial Research Tracker 3 (RT3)	•	•		•		•		
**Pedometers**								
MVP 4 Walk4Life Digital		•				•		•
Omron Walking Style Pro (HJ-720IT-E2)		•	•					•
Yamax Digi-Walker (SW-200)		•	•					•
Yamax Digi-Walker (SW-700)			•			•		•
Yamasa AM-5 Pedometer		•						
Pedometer (type not specified)		•	•					
**Proxy reported measurement tools**								
Children’s Leisure Activities Study Survey (CLASS)			•			•		
Children’s physical activity questionnaire (CPAQ)	•		•			•		
Habitual Activity Estimation Scale (HAES)			•					
Leisure time report			•			•		
Netherland’s Physical Activity Questionnaire (NPAQ)			•			•		
Nursery teacher’s report			•					
Pre School Physical Activity Questionnaire (PRE-PAQ)			•		•	•		
Questionnaire developed for parents of pre-schoolers in Mexico			•		•	•		
Teacher activity rating			•			•		
Teacher/mother reported habitual PA		•	•	•				
‘Toybox’ Primary Caregivers Questionnaire						•		
TV Diary			•			•		
7 day activity diary (adapted from CLASS)			•					

^a^Validity separated per level of evidence depending on the quality of measurement tool used (see Table 2 for full explanation)

### Risk of bias assessment

Risk of bias was assessed for all included studies using the modified Downs and Black checklist [38]. Studies consistently described the main aims of the research, the main outcomes to be measured, the exposures of interest and the main findings. In most cases, the staff, places and facilities were representative of what would usually be the case for the children under study, as testing often took place in nursery settings, at home, or was assessing habitual activity behaviours consisting of children’s usual behaviours and routines. However, a proportion of the studies were not reflective of usual activity for children due to them being laboratory based or involving structured protocols.

The main concern regarding potential bias of the studies was related to the lack of reporting of key information in the studies. This included lack of clarity on the representativeness of the sample population compared with the population from which they were recruited (*n* = 63). Also, many studies failed to consistently report reasons for drop out (e.g. non-completion, or missing or incomplete data) (*n* = 34). Many of the studies only reported the number of children included in analysis, but did not include the number who started the study sample, and so it was unclear as to whether drop out was an issue in these studies.

The majority of the studies demonstrated fair to good methodological quality ratings. Only two studies received a low methodological quality score [136, 137] and were removed from the overall summary analysis, however this did not affect the overall results of the review. The full Downs and Black risk of bias assessment for all included studies can be found in Additional file 11.

An additional risk of bias assessment was conducted on studies reporting proxy reported measurement tools, using the COSMIN risk of bias [43, 44]. The checklist demonstrated that the majority of tools examining convergent validity were of low quality; five studies were rated doubtful [100, 122, 125, 127, 128] and seven studies inadequate [68, 98, 120, 121, 123, 124, 126]. In the majority of the studies it was clear what the comparator tool measured and the statistical methods used were generally appropriate. Main concerns with the methodological quality of the studies related to the measurement properties of the comparator tools, with inadequate information provided. Proxy reported tools examined for test-retest reliability showed a range of quality, with some studies rated as adequate [122, 127, 128], two as doubtful [68, 121] and some inadequate [123, 124, 135]. The full COSMIN risk of bias assessment can be found in Additional file 12. This risk of bias assessment highlighted that the studies on proxy reported measurement tools were generally of low quality. However no studies were removed from the overall summary analysis based on this additional assessment, due to this evidence being the best available evidence for proxy reported measurement tools. The quality of the evidence is regarded in the interpretation of the studies throughout the review.

### Summary of measurement properties of measurement tools, separated by measurement property type

Results presented here are in line with the level of evidence scheme displayed in Table 2. The results will therefore be discussed as follows: Level 1 validity evidence where the tool under study is compared with calorimetry; level 2 validity, where the tool under study is compared with a measurement tool with relatively high validity e.g. direct observation; level 3 validity, where the tool under study is compared with a measurement tool with relatively low validity e.g. device based method such as accelerometry; level 4 validity, where two of the same type of measurement tool are compared where neither tool is considered to have known higher validity e.g. two makes of accelerometer; reliability; followed by feasibility. Detailed study tables of all included studies can be found in Additional files 4, 5, 6, 7, 8 and 9 presented by measurement property examined and separated by level of evidence. Where reported, these tables also include details on the interpretation choices used for device based measurement tools, including: cut points, epoch length, placement, wear time, non-wear time and valid number of days. It is critical that when using this review to help with measurement tool choice decisions, researchers should replicate the procedures in which the tool was validated (e.g. using the same cut points, epoch length and placement that the tool has shown to be valid for), which can all be found in the Additional files.

#### Level 1 validity

The criterion method of calorimetry (including DLW) for the outcome of energy expenditure was used in 12 studies. Multiple accelerometers were shown to have reasonable ability to determine energy expenditure, but often based on very limited evidence (one study only). There was however stronger evidence to suggest that the Actigraph (in particular the MTI and GT3X) and Actical were both able to determine EE and VO_2_ max [58–61], and the ActivPAL to determine EE [60, 65].

Table 4 provides a summary table of included studies that examined level 1 validity evidence of measurement tools (detailed information can be found in Additional file 4).

#### Level 2 validity

The most commonly used comparison methods for convergent validity of device based measurement tools was direct observation. Studies demonstrated that a number of device-based measurement tools were reasonably accurate at determining different PA and SB outcomes [81–84, 86, 93, 96]. The Actigraph accelerometer was the most frequently evaluated tool. Overall, these studies showed the Actigraph devices (in particular GT3X versions) had a good ability to determine SB, vigorous PA (VPA) and moderate to vigorous PA (MVPA) [61, 74–77, 79, 80]. Fewer studies evaluated the Actical accelerometer but these showed similar results [57, 62]. The ActivPAL accelerometer was shown to be suitable at assessing SB, MVPA and posture allocation [65, 87–89]; a unique quality that is not identified by other measurement tools. However, there is space for this to be developed further, as the accuracy of this measurement tool for identifying posture allocation is lower than in other population samples due to the amount of time that pre-school aged children will spend in ‘other’ postures, such as kneeling and crawling [138]. Fitbits also show some promising results for the measurement of SB, MVPA, total PA (TPA), and step counts [90, 91]; however, these conclusions are based on a very limited number of studies.

For pedometers, when compared against direct observation, study results were mixed but there is limited evidence to suggest that the Yamax Digiwalker SW-200 is able to determine step counts with reasonable accuracy [95, 96].

Table 5 provides a summary table of included studies that examined level 2 validity evidence of measurement tools (detailed information can be found in Additional file 5).

#### Level 3 validity

Level 3 validity evidence mainly consisted of proxy reported measurement tools and they were most frequently compared to accelerometry. Proxy reported measurement tools were generally poor at determining PA and SB outcomes. However, the Pre-PAQ was shown to be moderately accurate at determining stationary behaviour, light PA and VPA [122] and the *leisure time report* was able to determine MVPA [121]. The Netherland’s physical activity questionnaire and *nursery teacher’s report (based on Toyama Cohort Study survey questions)* could distinguish between different levels of activity [120, 123]. Although relatively few proxy reported tools demonstrated reasonable criterion or convergent validity, this could be due to a lack of face and content validity testing during the development of these tools [34, 139]. It is also worth noting that this evidence is based on very few, low quality studies. An advantage of the proxy reported tools over the other measurement tools is that they were able to capture the context and type of the behaviours, such as screen time, rather than just determine movement.

The direct observation protocol Observation System for Recording Physical Activity in Children- Preschool (OSRAC-P) showed promising agreement with a heart rate monitor and pedometer for determining different levels of activity [110]. Whilst the System for Observing Fitness Instruction Time for Preschoolers (SOFIT-P) did not demonstrate strong correlations with the output of the Actigraph (GT3X) [111].

The Fitbit (Flex) showed excellent ability to determine SB and TPA, but not MVPA, when compared with the Actigraph (GT3X+) [112]. For pedometers, activity counts from the Actigraph (CSA/MTI/GT1M) accelerometer were moderately correlated with step counts of the pedometer Yamax Digiwalker SW-200 [115, 116] and the Omron Walking Style Pro Pedometer (HJ-720IT-E2) [118] (values ranging from *r* = 0.64 to 0.92); suggesting that pedometers may be a plausible cheaper alternative to accelerometers in some instances [82].

Table 6 provides a summary table of included studies that examined level 3 validity evidence of measurement tools (detailed information can be found in Additional file 6).

#### Level 4 validity

A selection of studies included a comparison between two different makes of accelerometer each with unknown validity. These studies demonstrated that the combined heart rate with accelerometer, Actiheart, was shown to be similar in the activity count outcome to the accelerometers, Actical and RT3 (*r* = 0.80 to 0.95) [57]. Similarly, the Actigraph (GT1M) and RT3 showed reasonable similarity in activity count outcome [80] (*r* = 0.72). However, the majority of the studies did not show reasonable convergence, demonstrating that the various types of accelerometer can produce different outcomes [76, 78, 83, 129, 130]. The increased availability of accelerometers and differences in the outcomes of these studies demonstrates the importance of assessing validity of different devices simultaneously, alongside comparison measures [19]. There was weak comparisons between parental and teacher reported habitual physical activity [98, 100].

Table 7 provides a summary table of included studies that examined level 4 validity evidence of measurement tools (detailed information can be found in Additional file 7).

#### Face and content validity

Only two studies commented on the face and content validity of the proxy reported measurement tools [122, 124]. Face and content validity was determined by focus groups with parents and pre-school staff and consulting experts during questionnaire development [122]; and a pilot study with 21 parents, to determine the comprehension and reproducibility of the measure [124]. The Pre-PAQ included individual response options for both weekend days, due to parents indicating that children’s PA varied more on a weekend than during the week [122]. No further information was reported on the level of face and content validity within these studies; however, no major comprehension concerns were reported. There was minimal information about the procedures in these included studies and so it was not possible to assess the quality or provide firm conclusions on the content validity.

#### Test-retest reliability

Several of the proxy reported tools showed reasonable test-retest reliability (values ranging from *r* = 0.76 to 0.94) [121, 124, 127, 128] or variable test-retest results, whereby good test-retest for some items on the questionnaire but poor for other items [121–123, 135]. There was very limited evidence showing the test-retest of accelerometry and pedometry, with good test-retest for some activities but poor for others (ICC range from 0.34 to 0.87) [117]. Table 8 provides a summary table of included studies that examined reliability of measurement tools (detailed information can be found in Additional file 8).

#### Intra-instrument reliability

One accelerometer, the Fitbit (Zip) was the only tool to be examined for intra-instrument reliability in the included studies; showing excellent intra-instrument reliability (ICC = 0.91) when two devices were worn simultaneously on the right hip during a 5 minutes structured walking task in the nursery [91]. Table 8 provides a summary table of included studies that examined reliability of measurement tools (detailed information can be found in Additional file 8).

#### Feasibility

Whole room calorimetry was shown to be accepted by pre-school aged children as a way of measuring PA and SB [131, 132]. However, this method is expensive, cannot examine free living activity, may only be feasible for smaller scale projects and is highly burdensome on researchers due to the required training and expertise; thereby not being viable for surveillance and the majority of research projects [140]. Similarly, there were promising results for the feasibility of direct observation protocols [111, 133]. However, due to the intensive and demanding nature of direct observation, there are limits on the practicality of this method. Observations usually take place in just one location and for a short period of time, impacting the viability of this method in large samples and to identify habitual activity [140].

Feasibility and acceptability of device based measurement was generally high [73, 78, 115, 130, 134], even when more than one device was worn simultaneously [115, 130]. Although high acceptability of the ActivPAL accelerometer was reported [130], there was evidence of concerns of irritability of the ActivPAL accelerometer based devices due to these being attached directly to the skin [78, 88]. This may also be a concern when using other devices that attach in a similar way [54, 114]. The proportions of missing and excluded data, for reasons such as device malfunction or children not wearing the device for a sufficient period of time, should be considered when calculating the sample size for studies.

There were no studies that determined the feasibility of proxy reported measurement tools. Table 8 provides a summary table of included studies that examined feasibility of measurement tools (detailed information can be found in Additional file 9).

#### Generalisability of results

The majority of the studies reported the age (*n* = 74) and sex (*n* = 69) of the included children. However, only ten studies [66, 86, 99, 100, 111, 122, 123, 128, 134] reported other key attributes that help determine the generalisability of the results to the wider population, including: ethnic origin and socioeconomic profile (SEP). Therefore, there is insufficient evidence to suggest that results from the individual studies are generalisable across other populations.

### Ethnicity

Eighteen studies described the ethnicity of the sample in which the measurement property of the tool was examined. The majority of the studies that reported on ethnicity had samples with children whom were primarily white or Caucasian [57, 60, 66, 77, 85, 93, 94, 100, 122, 123, 134]. Three of the studies reported primarily Hispanic populations samples [99, 111, 128], followed by primarily African American samples [58, 63]. These was no indication of lower measurement properties of the tools in any specific ethnic group in these studies, however, this was not examined directly. Details of these studies are outlined in Additional files 4, 5, 6, 7, 8 and 9.

### Socioeconomic profile

Fourteen studies reported on the socioeconomic profile (SEP) of their sample. Some studies reported that at least some of the participants were recruited from pre-schools characterised to have individuals with lower SEP, such as Head Start Centres, which require proof of income to demonstrate that families are at or below poverty level [66, 86, 99, 111, 125, 128, 135]. The remaining studies that reported on SEP were based on individual level demographics. These studies reported that the samples were primarily made up of individuals of higher SEP [100, 122, 123, 134]. Only one study reported that the children in their sample were from lower to lower-middle SEP families [126]. Whilst one study also reported an equal amount of participants from both high and low SEP families [115]. None of the studies directly examined whether SEP affected validity or reliability, or whether there was reduced feasibility of the tools in different SEP groups. There was no evidence to suggest that SEP was affecting the validity or reliability of the measurement tools being evaluated. The majority of the studies found no indication of reduced feasibility amongst the different SEP, however, two studies conducted with participants of lower SEP reported a lack of feasibility when using pedometer-based measurement tools [99, 126]. Details of these studies are outlined in Additional files 4, 5, 6, 7, 8 and 9.

## Discussion

This systematic review identified 69 articles, describing 75 studies that were examining the measurement properties of measurement tools used to assess PA and/or SB in pre-school aged children. In this review, we provide an overview on what measurement tools have been examined for what outcome measures, with an indication on whether these have been shown to be valid, reliable or feasible.

The heterogeneity of the studies included in this review emphasises the complexity of measurement of PA and SB behaviours, identified previously by others [141, 142], alongside the additional challenges associated with measurement in a pre-school aged population [20, 143]. We show that different measurement tools often examine different dimensions of PA and SB (e.g. time spent in different intensities of activity, posture allocation, step count, energy expenditure) in line with previous literature [32, 144]. Measurement tools may all have a specific use depending on their derived purpose, desired measurement outcomes and the context in which the tool is being used [145]. However, when selecting an appropriate and useful measurement tool to assess PA and SB amongst children, there is often a trade-off between the three main utilities (validity, reliability, feasibility), alongside further considerations, such as the sample size of the study, budget, and availability of resources [144]. As such, it is not a question of which measurement tool is ‘best’ for assessing the PA and SB of pre-school aged children, but rather, what measure or combination of measures, are most appropriate in the given context for the desired outcome measures [55, 141, 144, 146, 147]. In line with this, the measurement properties of the tools are only reflective of the context in which they have been tested and cannot be generalised to other contexts, for example, if a tool showed good validity, reliability or feasibility but was examined in a laboratory based setting for use over a short period of time then the tool can only be said to be valid, reliable or feasible in this context, and the assessment does not apply to free living longer term measurement. We will, however, make recommendations based on our findings that we believe would be of interest to those involved in research based on the frequently used PA and SB outcomes.

Overall, based on the current evidence, which included a limited number of studies of varying quality, of the measurement properties of measurement tools used to examine PA and SB of pre-school aged children, multiple accelerometers, including the Actigraph (in particular GT3X versions), Actical, ActivPAL and Fitbits (Flex and Zip), can provide valid measures, with some evidence of feasibility, of movement-related behaviours that would be of interest in a range of research where resources and capacity allow. However, disadvantages include the need for expertise in the analysis of data, device malfunctions and continuous technological advances and development of new and improved activity monitoring devices, which increases difficultly for standardisation within and between studies [148, 149]. Alongside this, there are also differences on subjective decisions when using accelerometry, including: epochs, cut points, placement, wear time, non-wear time and valid number of days, which determines whether the data is valid or not [19, 20, 138, 150, 151]. Proxy report based measurement tools, in particular the PRE-PAQ and *leisure time report,* also show some promise, for some dimensions of PA and SB. Although, the evidence quality is weak, therefore, much more evaluation of the measurement properties of these types of tools is needed. These measurement tools have advantage in terms of identifying contextual behaviour, cost, accessibility and can be consistently used in a standardised way, as they are not reliant on upgrades in technology [152]. Additionally, whole room calorimetry and direct observation were shown to be accepted and feasible methods for measuring PA and SB of pre-school aged children. However, this was based on a very small number of studies and small sample sizes. As these methods are expensive, highly burdensome and intensive, require extensive training, and can only capture a short period of time, they may only be feasible for smaller scale projects and for only some dimensions of PA and SB.

Face and content validity have been suggested to be a crucial first step in determining the appropriateness of a measure, to determine if the measurement tool is assessing what it intends to measure and to identify whether the measure is understandable to the target population [34, 153, 154]. However, only two of the included studies reported on an element of face and content validity of their measures [122, 124].

A limited number of monitors were examined for reliability; the Fitbit Zip showed excellent intra-instrument reliability, however, this was based on one study only [91]. More research studies examining the intra-instrument reliability of measurement tools in pre-school aged children are warranted. Similarly, very few studies explicitly examined the feasibility, including acceptability, of the measurement tools [73, 78, 88, 99, 111, 114, 115, 128, 130–134], although, a proportion of the studies did report exclusion of participants and missing data. The measurement properties of measurement tools may be comprised if they are not feasible in the context and with the population in which they are to be used. The ability to identify reasons for missing data, reasons for non-compliance and overall acceptability and feasibility of the measurement tools, to determine end user experience, will help with understanding which tools are most applicable for use [36].

The review revealed a discrepancy in the amount of studies examining each measurement tool; with an emphasis placed on examining the measurement properties of device based measurement tools, primarily accelerometers, making up 70% of all studies, with multiple studies examining the same type of accelerometer. Accelerometers were also frequently used as a comparison measurement tool. A major limitation of the included studies utilising accelerometers was the considerable variation in the interpretation of data, due to differences in the subjective decisions on epochs, cut points, placement, wear time, non-wear time and valid number of days [19, 20, 138, 150, 151]. At least sixteen different published cut points were used in the included studies [57, 58, 63, 69–71, 84, 85, 101, 103–109], with some studies applying their own. Such methodological inconsistencies can be problematic, as applying different cut points to the same data can result in statistically and biologically significant differences in the outcomes of PA [102, 155, 156]. Although we provide reference to the published cut points used for each study in the summary tables, ultimately, the overall aims of the studies included in this review were not to show which data interpretation choices were most valid, and so we cannot determine which data interpretation choices are best to use. Therefore, it is important to note that the devices shown to be valid can only be said to be valid with the data interpretation choices in which they were tested. If researchers decide that a device based measurement tool is best for their research study, it is important to ensure use of the respective epochs, cut points and monitor placement outlined in the validation studies. We provide information on the interpretation choices used in each study in the tables outlined in Additional files 4, 5, 6 and 7.

Additionally, tools in the included studies were often evaluated over a short space of time, usually around 1 h, due to use of an intensive reference method (such as whole room calorimetry or direct observation). Consequently, wear time, non-wear time and information on the amount of valid number of days required for longer term data collection were not required. However, for examining PA, a full 7 days of measurement is preferred where possible [157, 158], with a minimum of 3 days required for reasonable estimates [159–161]. For examining SB, research suggests that ≥4 days of monitoring is required for reasonable estimates [162]. In addition to this, it is suggested that data include at least one weekend day [163]. Similarly, there are differences in the recommended number of hours wear time of the device, ranging from 6 to 10 hour wear time per day required for the most reliable estimates of PA [158–160, 164]. Collecting data using a 7 day wear protocol is recommended to increase the chances of there being enough data. Studies should ensure that they include the minimum amount of data, including both number of days and hours per day, to meet a reliability score of at least 0.7, and should report this in their study [163, 165]. Similarly, there is no consensus on defining non-wear time of accelerometry data [20, 138]. Non-wear time is often determined by consecutive zero counts in the data sets, however, this varies between studies and becomes more challenging when also examining SB using accelerometry [138]. A common and useful way in which non-wear time can be determined is by completion of a log to state when the accelerometer was removed and reasons for this; which can then be cross-validated with numbers of consecutive zeros within the data sets [20, 166].

The majority of studies included in this review were conducted in a free living (habitual) context, including at the pre-school and/or at home [32]. However, the results of the studies were often lacking in potential for generalisability and translation. As is the case for much research, identified studies have traditionally been conducted in high income countries. There was no evidence to suggest differences in the measurement properties of the measurement tools across different ethnicities or SEP, however a very small proportion of the studies explored these factors, and none directly. Similarly, there was minimal evidence of the measurement properties of the tools being evaluated with large sample sizes, so the capability of the tools at scale are unknown.

A major limitation in the field of PA and SB measurement is the lack of criterion methods for the majority outcomes, and indeed a lack of consensus on what might be considered a criterion. We used the level of evidence scheme to distinguish between differing levels of validity of the comparison measurement tools, but caution should always be applied when validity is measured against non-criterion methods, especially when the validity of these methods themselves are not well established.

### Strengths and weaknesses of the review

A primary strength of this review is that we assessed international literature for evidence on this topic, which included extensive searching of multiple platforms; published academic research articles through database searching, searches of the grey literature, and manual searches. Another strength of this review is that we examined various measurement properties of measurement tools; including the feasibility of the measures.

A potential weakness of this review was the risk of bias assessment, due to the tool that was used. Although we believe that we used the best available tool, it was not ideal because it was not devised specifically for studies examining the measurement properties of measurement tools. However, we did use an additional risk of bias assessment on proxy reported measurement tools due to this being available for these types of studies. Additionally, although two reviewers screened a 10% sample of the title and abstracts with very high agreement, only one reviewer screened the remaining studies and so it is plausible that studies were missed through human error.

A limitation of the research is that we did not explore the measurement property of ‘responsiveness’ in our search terms. Responsiveness, defined as ‘*The ability of a PROM to detect change over time in the construct to be measured*‘ [145], is a measurement property that is not well established in the field of PA and SB measurement. This is apparent from the literature [28, 29] and when conducting initial scoping searches for this review, no studies exploring responsiveness were identified. No studies were identified, also, through our additional hand searches. We are, therefore, currently unable to determine whether any proxy reported tools are able to detect changes in behaviour over time (e.g. in response to an intervention) and it is essential that this is the focus of future research.

### Implications for research

The lack of multiple studies examining the same measurement tool makes it difficult to produce firm conclusions on the measurement properties of some tools [28]. Differences in the number of studies examining each measurement tool, the ways in which the studies were conducted, the differences in comparison measurement tools used, the included samples, and reported outcome measures can make direct comparisons between the results of the studies difficult.

Future research should focus on the measurement properties of measurement tools being examined in different populations to ensure external validity of the measures and to extend the generalisability of the findings from these types of studies. This should include using large sample sizes, individuals with varying SEP and with different ethnicities, and conducted in lower income countries; this would help to evaluate the measurement properties, including feasibility and acceptability, of the measures in different contexts [16]. However, only after a measurement tool is shown to be valid, reliable, and feasible in the population for which it was originally developed [145].

In addition to this, research projects examining the PA or SB of pre-school aged children should look to identify the context in which the measurement properties of tools have been examined prior to choosing which tool may be most appropriate for their purpose. If the measurement properties of the selected tool are unknown in the context in which they are to be used, researchers should aim to conduct a validation check even if just on a sub-sample of children included in the study. This would be useful to identify whether the measurement tools are working as intended and to ensure greater confidence in the results of the study.

Our review highlights the importance of reporting on internal validity, such as missing data and non-completion within studies. Studies that reported such data revealed various important implications of using measurement tools, including that malfunctions of measurement tools, primarily with device based measurement tools and calorimetry, can have sufficient impact on the included final sample [76, 118, 122]. We highlight the need to examine and report missing data, as these considerations can substantially impact on the utility of a tool [56].

Further qualitative work in this area is needed, with two main purposes: 1) to determine the feasibility and acceptability of measurement tools and 2) to examine face and content validity. In some instances in this review, an indication of feasibility of the tools was provided based on numerical scores due to this being the only available feasibility data within the studies. However, we wish to highlight that the true feasibility of a measure cannot be expressed in numbers only, and much more qualitative work is needed in this area. This has been recognised in previous research, highlighting the importance of conducting qualitative research to examine the feasibility of measurement tools in the target population prior to use [54, 55, 139]; with such work being important to understand recruitment bias and reasons for missing data or non-completion [36, 56]. Additionally, qualitative work to determine validity is rarely conducted, demonstrated by only two studies included in the current review that commented on these aspects [122, 124]. However, face and content validity conducted using qualitative methods have been highlighted as a crucial first step in examining the validity of measurement tools for PA and SB, to determine whether tools are valid for their intended purpose [28, 32].

Future research should focus on further development and evaluation of proxy report methods together with the target population and ensure representativeness and feasibility of the measurement tool in the context in which it is intended to be used.

## Conclusions

The measurement tools used to measure PA and SB in pre-school aged children show mixed measurement properties, and were generally based on minimal studies providing variable quality of evidence. There is a clear need for further and more in-depth evaluation work. Based on currently available evidence, we conclude that the Actigraph (in particular GT3X versions), Actical and ActivPAL, have the greatest measurement properties for assessing common movement related outcomes (e.g. SB, MVPA, TPA) for free living activity of pre-school aged children, and should be the tool of choice where resources allow and where logistically possible. The Fitbit (Flex and Zip) also shows very promising results; however, these were based on a very limited sample of studies. Where measurement of a large sample is required and where budgets are limited, proxy measures can provide some valid data, alongside useful contextual information not captured by device-based measurement tools. A combination of accelerometers and proxy reported measurement tools (based on parent or carer reports) may be most useful for a range of PA and SB outcome measures.

## Supplementary Information

**Additional file 1.** PRISMA Checklist.

**Additional file 2.** Search strategy and outcomes.

**Additional file 3.** Excluded studies with reasons.

**Additional file 4. **Study details of level 1 validity evidence.

**Additional file 5. **Study details of level 2 validity evidence.

**Additional file 6. **Study details of level 3 validity evidence.

**Additional file 7. **Study details of level 4 validity evidence.

**Additional file 8. **Study details of reliability evidence.

**Additional file 9. **Study details of feasibility evidence.

**Additional file 10.** Source of funding for each study.

**Additional file 11. **Downs and Black risk of bias assessment.

**Additional file 12. **COSMIN risk of bias assessment.

## Data Availability

All data generated or analysed during this study are included in this published article and its supplementary information files.

## Abbreviations

AUC-ROC: Area under the receiver operating curve
COSMIN: COnsensus-based Standards for the selection of health status Measurement Instruments
DLW: Doubly labelled water
EPHPP: Effective Public Health Practice Project
LPA: Light physical activity
MVPA: Moderate to vigorous physical activity
PA: Physical activity
PRE-PAQ: Pre School Physical Activity Questionnaire
PRISMA: Preferred Reporting Items for Systematic Reviews and Meta-Analyses
PROSPERO: Prospective Register for Systematic Reviews
SB: Sedentary behaviour
SEP: Socioeconomic profile
TPA: Total physical activity
UK: United Kingdom
USA: United States of America
