# Supplementary material for: A systematic review of the validity, reliability, and feasibility of measurement tools used to assess the physical activity and sedentary behaviour of pre-school aged children
Source: Int J Behav Nutr Phys Act. 2021 Nov 4;18:141. doi: 10.1186/s12966-021-01132-9 (PMC8567581; doi:10.1186/s12966-021-01132-9)
Supplement: Supplementary file 2 — Additional file 2. Search strategy and outcomes. [file 12966_2021_1132_MOESM2_ESM.docx]

**Additional File 2: Search strategy and outcomes**

| Source of Search Conducted | Date of Search | Searches Conducted | Search Outcome |
| --- | --- | --- | --- |
| Scopus- *Science Direct* | 05/03/20 | ( TITLE-ABS-KEY ( "physical activit*" )  AND  TITLE-ABS-KEY ( sedentary  OR  sitting )  AND  TITLE-ABS-KEY ( valid*  OR  reliab*  OR  feasib* )  AND  TITLE-ABS-KEY ( pre*school  OR  "early years"  OR  "early childhood"  OR  "young children"  OR  kindergarten* )  AND  TITLE-ABS-KEY ( assess*  OR  measure*  OR  method ) ) | 203 |
|  |  | ( TITLE-ABS-KEY ( "physical activ*" )  AND  TITLE-ABS-KEY ( assess*  OR  measure*  OR  method )  AND  TITLE-ABS-KEY ( valid*  OR  reliab*  OR  feasib* )  AND  TITLE-ABS-KEY ( pre*school  OR  "early years"  OR  "early childhood"  OR  "young children"  OR  kindergarten* ) ) | 878 |
|  |  | ( TITLE-ABS-KEY ( sedentary  OR  sitting )  AND  TITLE-ABS-KEY ( assess*  OR  measure*  OR  method )  AND  TITLE-ABS-KEY ( valid*  OR  reliab*  OR  feasib* )  AND  TITLE-ABS-KEY ( pre*school  OR  "early years"  OR  "early childhood"  OR  "young children"  OR  kindergarten* ) ) | 346 |
| Web of Science- *Web of Science* | 05/03/20 | **ALL FIELDS: (**physical activit*) *AND* **ALL FIELDS:** (sedentary OR sitting) *AND* **ALL FIELDS:** (assess* OR measure* OR method) *AND* **ALL FIELDS:** (valid* OR reliab* OR feasib*) *AND* **ALL FIELDS:** (pre$school OR "early years" OR "early childhood" OR "young children" OR "kindergarten") | 282 |
|  |  | **ALL FIELDS:**(sedentary OR sitting) *AND* **ALL FIELDS:** (assess* OR measure* OR method) *AND* **ALL FIELDS:** (valid* OR reliab* OR feasib*) *AND* **ALL FIELDS:** (pre$school OR "early years" OR "early childhood" OR "young children" OR "kindergarten") | 329 |
|  |  | **ALL FIELDS: (**physical activit*) *AND* **ALL FIELDS:** (valid* OR reliab* OR feasib*) *AND* **ALL FIELDS:** (pre$school OR "early years" OR "early childhood" OR "young children" OR "kindergarten") *AND* **ALL FIELDS:** (assess* OR measure* OR method) | 993 |
| CINAHL, PsycARTICLES, PsycINFO, Medline SPORTdiscus- *EBSCOhost* | 05/03/20 | TX "physical activit*" AND TX ( sedentary OR sitting ) AND TX ( assess* OR measure* OR method ) AND TX ( valid* OR reliab* OR feasib* ) AND TX ( "pre#school" OR "early childhood" OR "early years" OR "young children" OR "kindergarten" ) | 487 |
|  |  | TX ( sedentary OR sitting ) AND TX ( assess* OR measure* OR method ) AND TX ( valid* OR reliab* OR feasib* ) AND TX ( "pre#school" OR "early childhood" OR "early years" OR "young children" OR "kindergarten" ) | 1,789 |
|  |  | TX "physical activit*" AND TX ( assess* OR measure* OR method ) AND TX ( valid* OR reliab* OR feasib* ) AND TX ( "pre#school" OR "early childhood" OR "early years" OR "young children" OR "kindergarten" ) | 2,293 |
| Journals- Pediatric Exercise Science *Human Kinetics Journals* | 05/03/20 | "physical activity" OR "sedentary" OR "sitting" AND "pre?school" OR "early years" OR "early childhood" OR "young children" OR "kindergarten" | 63 |
| Journals- Journal for the Measurement of Physical Behaviour- *Human Kinetics Journals* | 05/03/20 | "physical activity" OR "sedentary" OR "sitting" | 69 |
| Grey Literature- Opengrey.eu | 05/03/20 | "physical activity" OR "sedentary" AND "preschool" OR "early years" OR "young children" | 1408 |
| Grey Literature- Research Gate | 05/03/20 | "physical activity" OR "sedentary" AND "preschool" OR "early years" OR "young children" | 310 |
