# Supplementary material for: A systematic review of the validity, reliability, and feasibility of measurement tools used to assess the physical activity and sedentary behaviour of pre-school aged children
Source: Int J Behav Nutr Phys Act. 2021 Nov 4;18:141. doi: 10.1186/s12966-021-01132-9 (PMC8567581; doi:10.1186/s12966-021-01132-9)
Supplement: Supplementary file 3 — Additional file 3. Excluded studies with reasons. [file 12966_2021_1132_MOESM3_ESM.docx]

**Additional file 3: Excluded Studies with Reasons**

Total excluded following full paper screening (n=77).

**(Exclusion criteria 1) Study did not examine the utility of the tool (n=18):**

1. De Craemer, M., De Decker, E., Verloigne, M., De Bourdeaudhuij, I., Manios, Y., Cardon, G., & ToyBox-study group. (2016). The effect of a cluster randomised control trial on objectively measured sedentary time and parental reports of time spent in sedentary activities in Belgian preschoolers: the ToyBox-study. *International Journal of Behavioral Nutrition and Physical Activity*, *13*(1), 1.
2. Downing, K. L., Janssen, X., Cliff, D. P., Okely, A. D., & Reilly, J. J. (2019). Energy expenditure associated with posture transitions in preschool children. *PloS one*, *14*(4).
3. Flynn, J. I., Coe, D. P., Larsen, C. A., Rider, B. C., Conger, S. A., & Bassett, J. D. (2014). Detecting indoor and outdoor environments using the ActiGraph GT3X+ light sensor in children. *Medicine and science in sports and exercise*, *46*(1), 201-206.
4. LOGAN, N., REILLY, J. J., GRANT, S., & PATON, J. Y. (2000). Resting heart rate definition and its effect on apparent levels of physical activity in young children. *Medicine & science in sports & exercise*, *32*(1), 162.
5. Montgomery, C., Reilly, J. J., Jackson, D. M., Kelly, L. A., Slater, C., Paton, J. Y., & Grant, S. (2004). Relation between physical activity and energy expenditure in a representative sample of young children. *The American journal of clinical nutrition*, *80*(3), 591-596.
6. Mouratidou, T., Miguel, M. L., Androutsos, O., Manios, Y., De Bourdeaudhuij, I., Cardon, G., ... & Payr, A. (2014). Tools, harmonization and standardization procedures of the impact and outcome evaluation indices obtained during a kindergarten‐based, family‐involved intervention to prevent obesity in early childhood: the ToyBox‐study. *obesity reviews*, *15*, 53-60.
7. O'Connor, T. M., Cerin, E., Robles, J., Lee, R. E., Kerr, J., Butte, N., ... & Baranowski, T. (2013). Feasibility study to objectively assess activity and location of Hispanic preschoolers: a short communication. *Geospatial health*, *7*(2), 375.
8. Puyau, M. R., Adolph, A. L., Liu, Y., Wilson, T. A., Zakeri, I. F., & Butte, N. F. (2016). Energy cost of activities in preschool-aged children. *Journal of Physical Activity and Health*, *13*(s1), S11-S16.
9. Reilly, J. J., Janssen, X., Cliff, D. P., & Okely, A. D. (2015). Appropriateness of the definition of ‘sedentary’ in young children: Whole-room calorimetry study. *Journal of science and medicine in sport*, *18*(5), 565-568.
10. Saris, W. H. M., Snel, P., & Binkhorst, R. A. (1977). A portable heart rate distribution recorder for studying daily physical activity. *European journal of applied physiology and occupational physiology*, *37*(1), 17-25.- Age of children unknown, also does not appear to be evaluating the tools in relation to PA and/or SB, but rather just looking at heart rate.
11. Song, K. M., Bjornson, K. F., Cappello, T., & Coleman, K. (2006). Use of the StepWatch activity monitor for characterization of normal activity levels of children. *Journal of Pediatric Orthopaedics*, *26*(2), 245-249.

***Explored epoch length only (n=3)***

1. Colley, R. C., Harvey, A., Grattan, K. P., & Adamo, K. B. (2014). Impact of accelerometer epoch length on physical activity and sedentary behaviour outcomes for preschool-aged children. *Health Rep*, *25*(1), 3-9.
2. Kim, Y., Beets, M. W., Pate, R. R., & Blair, S. N. (2013). The effect of reintegrating Actigraph accelerometer counts in preschool children: Comparison using different epoch lengths. *Journal of science and medicine in sport*, *16*(2), 129-134.
3. Leeger-Aschmann, C. S., Schmutz, E. A., Zysset, A. E., Kakebeeke, T. H., Messerli-Bürgy, N., Stülb, K., ... & Puder, J. J. (2019). Accelerometer-derived physical activity estimation in preschoolers–comparison of cut-point sets incorporating the vector magnitude vs the vertical axis. *BMC public health*, *19*(1), 513.

***Tool explored PA environment, but not PA (n=2)***

1. Doerr, C. S., Bock, C., Fischer, J. E., & De Bock, F. (2014). Preschools’ Friendliness Toward Physical Activity: Item Battery and Two Scores Developed by Mixed Methods. *American journal of preventive medicine*, *46*(6), 593-604.
2. Park, S. H., Park, C. G., McCreary, L., & Norr, K. F. (2017). Cognitive Interviews for Validating the Family Nutrition Physical Activity Instrument for Korean-American Families With Young Children. *Journal of pediatric nursing*, *36*, 1-6

***Inter-observer reliability only (n=2)***

1. Brown, W. H., Pfeiffer, K. A., McIver, K. L., Dowda, M., Almeida, J. M., & Pate, R. R. (2006). Assessing preschool children's physical activity: the Observational System for Recording Physical Activity in children-preschool version. *Research quarterly for exercise and sport*, *77*(2), 167-176.
2. McIver, K. L., Brown, W. H., Pfeiffer, K. A., Dowda, M., & Pate, R. R. (2009). Assessing children's physical activity in their homes: The observational system for recording physical activity in children‐home. *Journal of applied behavior analysis*, *42*(1), 1-16.

**(Exclusion Criteria 2) Age of children outside range (n=31):**

***Included children under 3 years (n=10)***

1. Bingham, D., Collings, P., Clemes, S., Costa, S., Santorelli, G., Griffiths, P., & Barber, S. (2016). Reliability and validity of the early years physical activity questionnaire (EY-PAQ). *Sports*, *4*(2), 30.
2. Burdette, H. L., Whitaker, R. C., & Daniels, S. R. (2004). Parental report of outdoor playtime as a measure of physical activity in preschool-aged children. *Archives of pediatrics & adolescent medicine*, *158*(4), 353-357.
3. De, F. B., Menze, J., Becker, S., Litaker, D., Fischer, J., & Seidel, I. (2010). Combining accelerometry and HR for assessing preschoolers' physical activity. *Medicine and science in sports and exercise*, *42*(12), 2237-2243.
4. Henriksson, H., Forsum, E., & Löf, M. (2014). Evaluation of Actiheart and a 7 d activity diary for estimating free-living total and activity energy expenditure using criterion methods in 1· 5-and 3-year-old children. *British Journal of Nutrition*, *111*(10), 1830-1840
5. Klesges, L. M., & Klesges, R. C. (1987). The assessment of children's physical activity: a comparison of methods. *Medicine and Science in Sports and Exercise*, *19*(5), 511-517.
6. KLESGES, R. C., Klbsges, L. M., Swenson, A. M., & Pheley, A. M. (1985). A validation of two motion sensors in the prediction of child and adult physical activity levels. *American journal of epidemiology*, *122*(3), 400-410.
7. Rice, K. R., Joschtel, B., & Trost, S. G. (2013). Validity of family child care providers' proxy reports on children's physical activity. *Childhood Obesity*, *9*(5), 393-398.
8. Sarker, H., Anderson, L. N., Borkhoff, C. M., Abreo, K., Tremblay, M. S., Lebovic, G., ... & Birken, C. S. (2015). Validation of parent-reported physical activity and sedentary time by accelerometry in young children. *BMC research notes*, *8*(1), 735.
9. Vale, S., Santos, R., Silva, P., Soares-Miranda, L., & Mota, J. (2009). Preschool children physical activity measurement: importance of epoch length choice. *Pediatric exercise science*, *21*(4), 413-420.
10. Wiseman, N., Harris, N., & Downes, M. (2017). Validation of an iPad activity to measure preschool children’s food and physical activity knowledge and preferences. *international journal of behavioral nutrition and physical activity*, *14*(1), 11.

***Included children over 7 years (n=17)***

1. Aadland, E., Andersen, L. B., Ekelund, U., Anderssen, S. A., & Resaland, G. K. (2018). Reproducibility of domain-specific physical activity over two seasons in children. *BMC public health*, *18*(1), 821.
2. Bender, J. M., Brownson, R. C., Elliott, M. B., & Haire-Joshu, D. L. (2005). Children's physical activity: using accelerometers to validate a parent proxy record. *Medicine and science in sports and exercise*, *37*(8), 1409.
3. Busser, H. J., Ott, J., Van Lummel, R. C., Uiterwaal, M., & Blank, R. (1997). Ambulatory monitoring of children's activity. *Medical engineering & physics*, *19*(5), 440-445.
4. Cale, L. (1993). *Monitoring physical activity in children* (Doctoral dissertation, Loughborough University).
5. Camargo, D. M., Santisteban, S., Paredes, E., Flórez, M. A., & Bueno, D. (2015). Confiabilidad de un cuestionario para medir la actividad física y los comportamientos sedentarios en niños desde preescolar hasta cuarto grado de primaria. *Biomédica*, *35*(3), 347-356.
6. Cohen, A., McDonald, S., McIver, K., Pate, R., & Trost, S. (2014). Assessing physical activity during youth sport: the observational system for recording activity in children: youth sports. *Pediatric exercise science*, *26*(2), 203-209.
7. Duncan, J. S., Badland, H. M., & Schofield, G. (2009). Combining GPS with heart rate monitoring to measure physical activity in children: A feasibility study. *Journal of Science and Medicine in Sport*, *12*(5), 583-585.
8. Going, S. B., Levin, S., Harrell, J., Stewart, D., Kushi, L., Cornell, C. E., ... & Sallis, J. (1999). Physical activity assessment in American Indian schoolchildren in the Pathways study. *The American journal of clinical nutrition*, *69*(4), 788S-795S.
9. Hendrick, P., Boyd, T., Low, O., Takarangi, K., Paterson, M., Claydon, L., & Milosavljevic, S. (2010). Construct validity of RT3 accelerometer: A comparison of level-ground and treadmill walking at self-selected speeds. *Journal of Rehabilitation Research & Development*, *47*(2).
10. Mehdizadeh, A., Shafiee, M., Khadem-Rezaiyan, M., Sardar, M. A., Vatanparast, H., Rose, E., ... & Nematy, M. (2019). Evidence for the Validity of the Children's Attraction to Physical Activity (CAPA) Scale in Iranian Preschool Children. *Journal of pediatric nursing*, *44*, e52-e57.
11. Nascimento‐Ferreira, M. V., De Moraes, A. C. F., Toazza‐Oliveira, P. V., Forjaz, C. L., Aristizabal, J. C., Santaliesra‐Pasías, A. M., ... & Moreno, L. A. (2018). Reliability and validity of a questionnaire for physical activity assessment in South American children and adolescents: the SAYCARE study. *Obesity*, *26*, S23-S30.
12. Nguyen, D. M. T., Lecoultre, V., Sunami, Y., & Schutz, Y. (2013). Assessment of physical activity and energy expenditure by GPS combined with accelerometry in real-life conditions. *Journal of Physical Activity and Health*, *10*(6), 880-888.
13. Ojiambo, R., Konstabel, K., Veidebaum, T., Reilly, J., Verbestel, V., Huybrechts, I., ... & Bammann, K. (2012). Validity of hip-mounted uniaxial accelerometry with heart-rate monitoring vs. triaxial accelerometry in the assessment of free-living energy expenditure in young children: the IDEFICS Validation Study. *Journal of Applied Physiology*, *113*(10), 1530-1536.
14. Rowe, P. J., Schuldheisz, J. M., & Van Der Mars, H. (1997). Validation of SOFIT for measuring physical activity of first-to eighth-grade students. *Pediatric Exercise Science*, *9*(2), 136-149.
15. Rowlands, A.V. (1998). The measurement of physical activity in children, (Doctoral dissertation, Prifysgol Bangor University).
16. Van Camp, C. M., & Berth, D. (2018). Further evaluation of observational and mechanical measures of physical activity. *Behavioral Interventions*, *33*(3), 284-296.
17. Van Camp, C. M., & Hayes, L. B. (2017). Identifying beneficial physical activity during school recess: Utility and feasibility of the Fitbit. *Journal of Behavioral Education*, *26*(4), 394-409.

***Mean of children >6 years (n=3)***

1. Basterfield, L., Adamson, A. J., Parkinson, K. N., Maute, U., Li, P. X., Reilly, J. J., & Gateshead Millennium Study Core Team. (2008). Surveillance of physical activity in the UK is flawed: validation of the Health Survey for England Physical Activity Questionnaire. *Archives of disease in childhood*, *93*(12), 1054-1058.
2. Tanaka, C., Tanaka, S., Kawahara, J., & Midorikawa, T. (2007). Triaxial accelerometry for assessment of physical activity in young children. *Obesity*, *15*(5), 1233-1241.
3. Sekine, M., Yamagami, T., Chen, X., Hayashikawa, Y., Hamanishi, S., & Kagamimori, S. (2002). Validity of a questionnaire evaluating physical activity level in young children. *Environmental health and preventive medicine*, *6*(4), 264-267.

***Age not specified (n=1)***

1. Ridgers, N. D., Stratton, G., & McKenzie, T. L. (2010). Reliability and validity of the System for Observing Children’s Activity and Relationships during Play (SOCARP). *Journal of Physical Activity and Health*, *7*(1), 17-25.-

**(Exclusion Criteria 3): Children with conditions or chronic disease (n=1):**

1. Anderson, D. R., Field, D. E., Collins, P. A., Lorch, E. P., & Nathan, J. G. (1985). Estimates of young children's time with television: a methodological comparison of parent reports with time-lapse video home observation. Child development, 1345-1357.

**(Exclusion Criteria 4) Wear time only (n=13):**

1. Aadland, E., & Johannessen, K. (2015). Agreement of objectively measured physical activity and sedentary time in preschool children. *Preventive medicine reports*, *2*, 635-639.
2. Addy, C. L., Trilk, J. L., Dowda, M., Byun, W., & Pate, R. R. (2014). Assessing preschool children’s physical activity: how many days of accelerometry measurement. *Pediatric exercise science*, *26*(1), 103-109-
3. Lima, R. A., Barros, S. S. H., Cardoso Júnior, C. G., Silva, G., Farias Júnior, J. C. D., Andersen, L. B., & Barros, M. V. G. D. (2014). Influence of number of days and valid hours using accelerometry on the estimates of physical activity level in preschool children from Recife, Pernambuco, Brazil. *Revista Brasileira de Cineantropometria & Desempenho Humano*, *16*(2), 171-181.
4. Byun, W., Beets, M., & Pate, R. (2015). Sedentary behavior in preschoolers: How many days of accelerometer monitoring is needed?. *International journal of environmental research and public health*, *12*(10), 13148-13161.
5. DuRant, R. H., Baranowski, T., Davis, H. A. R. R. Y., Rhodes, T. H. O. M. A. S., Thompson, W. O., Greaves, K. A., & Puhl, J. A. C. Q. U. E. L. I. N. E. (1993). Reliability and variability of indicators of heart-rate monitoring in children. *Medicine and Science in Sports and Exercise*, *25*(3), 389-395.
6. Durant, R. H., Baranowski, T., Davis, H. A. R. R. Y., Thompson, W. O., Puhl, J. A. C. Q. U. E. L. I. N. E., Greaves, K. A., & Rhodes, T. H. O. M. A. S. (1992). Reliability and variability of heart rate monitoring in 3-, 4-, or 5-yr-old children. *Medicine and science in sports and exercise*, *24*(2), 265-271.
7. DuRANT, R. H., Baranowski, T., Puhl, J., Rhodes, T., Davis, H., Greaves, K. A., & Thompson, W. O. (1993). Evaluation of the Children's Activity Rating Scale (CARS) in young children. *Medicine & Science in Sports & Exercise*
8. Hinkley, T., O’CONNELL, E. O. I. N., Okely, A. D., Crawford, D., Hesketh, K., & Salmon, J. (2012). Assessing volume of accelerometry data for reliability in preschool children. *Medicine & Science in Sports & Exercise*, *44*(12), 2436-2441.
9. Hislop, J., Law, J., Rush, R., Grainger, A., Bulley, C., Reilly, J. J., & Mercer, T. (2014). An investigation into the minimum accelerometry wear time for reliable estimates of habitual physical activity and definition of a standard measurement day in pre-school children. *Physiological Measurement*, *35*(11), 2213.
10. Kang, M., Bjornson, K., Barreira, T. V., Ragan, B. G., & Song, K. (2014). The minimum number of days required to establish reliable physical activity estimates in children aged 2–15 years. *Physiological measurement*, *35*(11), 2229.
11. Penpraze, V., Reilly, J. J., MacLean, C. M., Montgomery, C., Kelly, L. A., Paton, J. Y., ... & Grant, S. (2006). Monitoring of physical activity in young children: how much is enough?. *Pediatric Exercise Science*, *18*(4), 483-491.
12. Ricardo, L. I. C., Wendt, A., Galliano, L. M., de Andrade Muller, W., Niño Cruz, G. I., Wehrmeister, F., ... & Silva, C. M. (2020). Number of days required to estimate physical activity constructs objectively measured in different age groups: Findings from three Brazilian (Pelotas) population-based birth cohorts.
13. Toschke, J. A., von Kries, R., Rosenfeld, E., & Toschke, A. M. (2007). Reliability of physical activity measures from accelerometry among preschoolers in free-living conditions. *Clinical nutrition*, *26*(4), 416-420.

**(Exclusion Criteria 5) Calibration/prediction equation only (n=10):**

1. Ahmadi, M. N., Brookes, D., Chowdhury, A., Pavey, T., & Trost, S. G. (2019). Free-living Evaluation of Laboratory-based Activity Classifiers in Preschoolers. *Medicine and science in sports and exercise*.
2. Bornstein, D. B., Beets, M. W., Byun, W., Welk, G., Bottai, M., Dowda, M., & Pate, R. (2011). Equating accelerometer estimates of moderate-to-vigorous physical activity: in search of the Rosetta Stone. *Journal of Science and Medicine in Sport*, *14*(5), 404-410.
3. Butte, N. F., Wong, W. W., Lee, J. S., Adolph, A. L., Puyau, M. R., & Zakeri, I. F. (2014). Prediction of energy expenditure and physical activity in preschoolers. *Medicine and science in sports and exercise*, *46*(6), 1216.
4. Johansson, E., Larisch, L. M., Marcus, C., & Hagströmer, M. (2016). Calibration and validation of a wrist-and hip-worn actigraph accelerometer in 4-year-old children. *PloS one*, *11*(9), e0162436.
5. Li, S. (2019). *Machine Learning Approaches to Calibrate Wrist-Worn Accelerometry for Physical Activity Assessment in Preschoolers* (Doctoral dissertation, The University of Texas at San Antonio).
6. Roscoe, C. M., James, R. S., & Duncan, M. J. (2017). Calibration of GENEActiv accelerometer wrist cut-points for the assessment of physical activity intensity of preschool aged children. *European journal of pediatrics*, *176*(8), 1093-1098.-calibration?
7. Tanaka, C., Hikihara, Y., Ando, T., Oshima, Y., Usui, C., Ohgi, Y., ... & Tanaka, S. (2019). Prediction of Physical Activity Intensity with Accelerometry in Young Children. *International journal of environmental research and public health*, *16*(6), 931.
8. Trost, S. G., Cliff, D., Ahmadi, M., Van Tuc, N., & Hagenbuchner, M. (2018). Sensor-enabled activity class recognition in preschoolers: Hip versus wrist data. *Medicine and science in sports and exercise*, *50*(3), 634-641.
9. van Cauwenberghe, V., Labarque, V., Trost, S. G., De Bourdeaudhuij, I., & Cardon, G. (2011). Calibration and comparison of accelerometer cut points in preschool children. *International Journal of Pediatric Obesity*, *6*(sup3), e582-589.
10. Zakeri, I. F., Adolph, A. L., Puyau, M. R., Vohra, F. A., & Butte, N. F. (2013). Cross-sectional time series and multivariate adaptive regression splines models using accelerometry and heart rate predict energy expenditure of preschoolers. *The Journal of nutrition*, *143*(1), 114-122.

***(Exclusion Criteria 6) Review (n=1):***

1. Rowlands, A. V., Eston, R. G., & Ingledew, D. K. (1997). Measurement of physical activity in children with particular reference to the use of heart rate and pedometry. *Sports Medicine*, *24*(4), 258-272.

**(Exclusion Criteria 7) Insufficient information available (n=1):**

1. Downing, K. L., Janssen, X., & Reilly, J. J. (2019). Feasibility of wearable cameras to assess screen time and time spent restrained in children aged 3 to 5 years: a study protocol. *BMJ open*, *9*(5), e028265.

**(Exclusion Criteria 8) Thesis, but associated papers had already been included (n=2):**

1. Kelly, L. (2005). "Objectively measured physical activity and sedentary behaviour in young children."
2. Byun, W. (2013). Sedentary behavior in preschool children: Studies of reliability, level, and effect of objectively measured sedentary behavior, ProQuest Information & Learning. 73
