# Supplementary material for: A systematic review of the validity, reliability, and feasibility of measurement tools used to assess the physical activity and sedentary behaviour of pre-school aged children
Source: Int J Behav Nutr Phys Act. 2021 Nov 4;18:141. doi: 10.1186/s12966-021-01132-9 (PMC8567581; doi:10.1186/s12966-021-01132-9)
Supplement: Supplementary file 4 — Additional file 4. Study details of level 1 validity evidence. [file 12966_2021_1132_MOESM4_ESM.docx]

**Additional File 4: Study details of level 1 validity evidence (n=12)**

| Study details | Methods | | | Units of measure | Validity results |
| --- | --- | --- | --- | --- | --- |
|  | **Measurement tool(s) under study**  *Placement, epoch, cut points, wear time, non-wear time and number of valid days* | **Criterion Method** | **Study protocol** *(lab/free living)* |  |  |
| **Combined heart rate and accelerometry and accelerometers (n=1)** | | | | | |
| **PA and SB (n=1)** | | | | | |
| Adolph et al. (2012) [57];  USA;  n=64; 3-5 years, mean age=4.5 years;  37 male, 27 female  50% White, 27% Black, 22% Hispanic, 2% Asian | **Actiheart (MiniMitter)**  *Chest*  *15 sec epoch*  **Actical (Respironics, MiniMitter)**  *Right hip*  *15 sec epoch*  **Triaxial Research Tracker 3 (RT3- Stayhealthy)**  *Right hip*  *1 sec epoch*  *Cut points: Piecewise linear regressions determined thresholds into SB, LPA, MPA, VPA* | Whole room Calorimetry | 3 hour whole room calorimeter protocol *(laboratory based)* | Accelerometers: activity counts per minute  Room respiration calorimetry: HR, EE, AEE, physical activity rating | **Pearson’s Correlation:**  In increasing order - Actical, Actiheart, RT3 total counts were positively correlated with AEE and physical activity ratings r=0.69-0.82 (p=0.001).  Actiheart significantly correlated with activity energy expenditure r=0.72 (p<0.001).  **Overall classification accuracy of accelerometers compared to calorimetry:**  Actical -71%  Actiheart – 72%  RT3-73% |
| **Accelerometers (n=10)** | | | | | |
| **PA and SB (n=3)** | | | | | |
| Janssen et al. (2013a) [61];  Australia  N=40; 4-6 years, mean age 5.3 years;  22 male, 18 female. | **Actigraph (GT3X)**  *Right hip*  *15 sec epoch*  *Cut points: Activity energy expenditure and* VO_2_*: Puyau et al., 2002, Pate et al., 2006; and activity intensity:*  *Puyau et al., 2002, Pate et al., 2006, Evenson et al., 2008, Sirard et al., 2005, Van Cauwenberghe et al., 2011 and Reilly et al., 2003* | Whole room calorimetry | 150 minute structured activity laboratory protocol *(lab based)* | Actigraph- Activity counts converted to activity energy expenditure, VO_2_ and activity intensity (SB, LPA, MVPA).  Whole room calorimetry- activity energy expenditure | **Dependent t-tests, with Bonferroni correction:**  **VO_2_-**  Actigraph using Pate cut point significantly overestimated VO_2_ during SB and LPA and for total VO_2_ (p<0.001) but did not show a significant difference between measured and predicted VO_2_ during MVPA (p=0.072).  **Energy expenditure-**  Actigraph using Puyau cut point significantly underestimated AEE during MVPA and LPA and for total AEE (p<0.0125) but did not show a significant difference for activity energy expenditure during SB (p=0.5481).  **Classification accuracy determined using ROC-AUC, sensitivity, specificity:**  **Both PA intensity and EE:**  Classification accuracy for Evenson cut point for SB (ROC-AUC = 0.90, sensitivity = 90.7%, specificity= 89.7%), LPA (ROC-AUC = 0.76, sensitivity = 69.9%, specificity= 82.2%) and MVPA (ROC-AUC = 0.76, sensitivity = 60.5%, specificity= 91%); higher accuracy compared to all others expect Pate cut point for MVPA (ROC-AUC = 0.78, sensitivity = 69.6%, specificity= 86.4%). |
| Janssen et al. (2014) [65];  Australia;  N=18; 4-6 years, mean age 5.2 years;  9 male, 9 female | **ActivPAL**  *Thigh*  *15 sec epoch*  *Cut points: produced during ROC curve analysis* | Whole room calorimetry | 150 minute room calorimeter protocol *(laboratory based)* | ActivPAL- activity counts to determine MVPA and METs equation  Whole room calorimetry – EE | **Classification accuracy determined by ROC-AUC, sensitivity, specificity:**  ActivPAL METs equation overestimated METs during SB (+6.0%) and underestimated METs for LPA (-15.3%), MVPA (-32.8%) ad total METs (-13.6%) (all p<0.001).  Classification accuracy for activPAL determined MVPA when using both EE and direct observation as criterion: Sensitivity = 94.8%, Specificity = 84.8%, ROC-AUC = 0.90. |
| Janssen et al. (2015) [62];  Australia;  N=40, 4-6 years, mean age 5.3 years;  22 male, 18 female | **Actical (Respironics, Philips)**  *Right hip*  *15 and 60 sec epoch*  *Cut points: Pfeiffer et al., 2006, Adolph et al., 2012 and Evenson et al., 2008* | Whole room calorimetry | 150 minute room calorimeter protocol *(laboratory based)* | Actical- AEE; activity intensity - SB, LPA, MVPA  Whole room calorimetry – Energy expenditure | Actical for predicting AEE:  **Dependent t-test:**  Pfeiffer equation overestimated AEE during SB and underestimated AEE during LPA (0.017 vs 0.005 kcal⋅kg^-1^⋅min^-1^, 0.023 vs 0.030 kcal⋅kg^-1^⋅min^-1^, respectively; both p<0.01). No difference for MVPA (p=0.76) or total AEE (p=0.80).  **Classification accuracy determined by ROC-AUC, sensitivity and specificity :**  Broken down by intensity:  SB: Adolph (15s): ROC-AUC=0.80, sensitivity = 89.1%, specificity= 71.4%  Adolph (60s): ROC-AUC=0.82, sensitivity = 82.5%, specificity=81.1%  Evenson (15s): ROC-AUC=0.79, sensitivity = 91%, specificity= 68%  Classiﬁcation accuracy was signiﬁcantly higher for Adolph compared to Evenson (P < 0.05).  LPA: Adolph (15s): ROC-AUC=0.68, sensitivity = 51.9%, specificity= 84.3%  Adolph (60s): ROC-AUC=0.73, sensitivity = 66.2%, specificity=80.2%  Evenson (15s): ROC-AUC=0.65, sensitivity = 51%, specificity= 79.1%  Adolph showed a signiﬁcantly higher sensitivity (66.2%), which resulted in signiﬁcantly higher classiﬁcation accuracy compared to Evenson (P < 0.05).  MVPA: Adolph (15s): ROC-AUC=0.82, sensitivity = 71%, specificity= 93.6%  Adolph (60s): ROC-AUC=0.85 sensitivity = 74.3%, specificity=95.3%  Evenson (15s): ROC-AUC=0.75, sensitivity = 54.9%, specificity= 96%  Pfeiffer (15s): ROC-AUC=0.70, sensitivity = 43.9%, specificity= 96.5%  Classiﬁcation accuracy was signiﬁcantly higher for Adolph compared to all others (p< 0.05).  Actical compared with direct observation and EE:  **Kappa:**  κ values were slightly increased compared to using direct observation only. Adolph (κ = 0.72, 95% CI = 0.70–0.74), Adolph(15s) (κ = 0.65, 95% CI = 0.64–0.65) and Evenson (κ = 0.61 95% CI = 0.60–0.62) exhibited substantial agreement predicting PA and SB determined by DO and calorimetry.  **Classification accuracy determined by ROC-AUC, sensitivity and specificity :**  ROC-AUC values when using direct observation combined with EE were slightly higher but similar to using direct observation only.  Adolph was signiﬁcantly better than all others (P < 0.05) when classifying LPA and MVPA. For SB, classiﬁcation accuracy was good for the Adolph and Evenson (ROC-AUC = 0.82–0.85) with Adolph performing signiﬁcantly better than Evenson. However, when comparing Evenson and Adolph(15s) this difference disappeared. |
| **PA (n=7)** | | | | | |
| Nyström et al. (2017) [64];  Sweden;  N=40; 5.2- 5.7 years, mean age 5.5 years;  22 male, 18 female | **Actigraph (wGT3X-BT)**  *Non-dominant wrist*  *1 sec epoch*  *No cut points reported for PA*  *Wear time:*  *When the awake wearing time (non-sleeping data) was ≥600 minutes*  *Non-wear time:* *Determined using raw acceleration data. SD of each axis calculated over a 30-min period. If SD of acceleration of any two axes less than 0.002 g for the same window= non-wear time. Non-wear time confirmed by diary completed by parents.*    *Number of days: Minimum of 3 days of valid data.* | Doubly labelled water (DLW) | 7 days activity *(free living)* | Actigraph- Counts per minute to determine AEE  DLW- TEE and AEE | **Bland Altman:**  Mean difference between the methods was 2.4kj/24h with limits of agreement of 621.6kj/24h (mean VM total) and mean difference -2.4kj/24h with limits of agreement of 584.8kj/24h (mean VM waking). |
| Pate et al. (2006) [58];  USA;  N=29; 3-5 years, mean age 4.4 years;  13 male, 16 female  55.2% African American,  44.8% White | **Actigraph (MTI 7164)**  *Right hip*  *15 sec epoch*  *Cut points: Pate et al., 2006* | Indirect calorimetry-Cosmed portable metabolic system (Model K4b2) | 20 minutes of unstructured activites in pre-school setting *(usual activity- children were wearing metabolic system at time of activity and were asked to select new activity every 4-6 minutes)* | Actigraph- activity counts  Cosmed metabolic system- VO_2_ estimation | **Spearman’s correlation:**  Correlation between the measurement tools was r=0.66 (p<0.001).  **Pearson’s correlation:**  Correlation between VO_2_ and accelerometer counts across the activities was r=0.82  **Intraclass correlation coefficient:**  Measured and predicted VO_2_ was r=0.57.  **% agreement, kappa, modified kappa, sensitivity, specificity:**  MVPA: % agreement was 0.69, kappa 0.36, modified kappa 0.38. Sensitivity 96.6% and specificity 86.2%.  VPA: % agreement was 0.81, kappa 0.13 and modified kappa 0.62. Sensitivity 65.5% and specificity 95.4%. |
| Reilly et al. (2006) [59];  Scotland, UK;  N=85; 3-6 years, mean age 4.6 years;  51 male, 34 female | **Actigraph (CSA/MTI***)*  *Right hip*  *1 min epoch*  *Cut points: Ekelund et al., 2001 and Puyau et al., 2002*  *Wear time: >360 minutes*  *Non wear time:*  *Parents to record when accelerometer removed*  *Valid n of days: 3 days (2 weekdays and 1 weekend day)* | Doubly labelled water (DLW) | 3 days of habitual activity *(free living)* | Actigraph-activity counts to determine TEE  DLW- TEE | **Bland Altman:**  Ekelund equation-  Mean paired difference between predicted TEE (Actigraph data) and DLW was +0.3 MJ/d. Difference was not statistically significant (p=0.10), limits of agreement were +4.3 and -3.7 MJ/d. Errors were significantly negatively correlated with TEE (r=-0.65, p<0.001). Errors in estimation larger in boys compared with girls.  Puyau equation-  Mean paired difference between TEE (Actigraph data) and DLW was -0.3 MJ/d. Difference was not statistically significant (p<0.10), limits of agreement were +3.2 to -3.8 MJ/d. Errors were significantly negatively correlated with TEE r=-0.89 (p<0.001). No gender differences in accuracy. |
| Steenbock et al. (2019);  Germany; [60]  n=41; 3yrs – 6.3 years, mean age 4.8 years;  22 male, 19 female  100% Caucasian | **GENEActiv**  *Right hip, left wrist, right wrist*  **Actigraph (GT3X***)*  *Left and right hip*  **ActivPAL**  *Thigh*  *Cut points: Regressions used to predict energy expenditure and METs* | Indirect calorimetry: MetaMax3b | 75 minute protocol of both pre-determined and free play activities *(usual activity)* | Accelerometers – EE and METs  Indirect calorimetry - EE | **Root mean squared error (RMSE):**  For predicting EE: GENEActiv devices obtained mean RMSE of 2.56 kJ/min (left and right wrist) and 2.73kJ/min(right hip); GT3X on the left and right hip obtained mean RMSE of 2.60 and 2.74 kJ/min, respectively; thigh worn activPAL provided a mean RMSE of 2.76kJ/min. |
| Pfeiffer et al. (2006) [63];  USA;  n=18; 3.4-5.7 years, mean age 4.4 years ;  7 male, 11 female  89% African American | **Actical (MiniMitter)**  *Right hip*  15 sec epoch  *Cut points: Pfeiffer et al., 2006* | Indirect calorimetry-Cosmed portable metabolic system (Model K4b2) | 20 minutes of unstructured activities at the pre-school, but child instructed to choose a different activity every 6 minutes *(usual activity)* | Actical: Activity counts to determine VPA and MVPA, and estimated VO_2_  Cosmed: VO_2_ | **Intraclass correlation coefficient:**  ICC between VO_2_ estimated from Actical counts and by Cosmed = 0.59 using log transformed data.  **Spearman’s correlation:**  Spearman correlation coefficient between the VO_2_ estimated from Actical and Cosmed r=0.80 (p<0.001).  **% agreement and Cohen’s kappa:**  Percent agreement, kappa, modified kappa for VPA were: 0.85, 0.26 and 0.71 respectively.  Percent agreement, kappa, modified kappa for MVPA were: 0.73, 0.40, 0.46 respectively. |
| Lopez‐Alarcon et al. (2004) [66];  USA;  N=29; 4-6 years, mean age 4.9 years;  17 male, 12 female  58% White  42% African American  6 of the children recruited from Head Start Center | **Actiwatch (AW16)**  *Right ankle*  *1 min epoch* | Doubly labelled water (DLW) | 8 days of habitual activity- first day not included in the analyses *(free living)* | Actiwatch: Activity counts  DLW: TEE | **Pearson’s correlation:**  Activity counts and TEE were not significantly associated r=0.27 (p=0.15).  **Multiple linear regression analyses:**  Activity counts were not significantly related to total energy expenditure in any regression models (p=0.31). |
| Sijtsma et al. (2013) [67];  Netherlands;  N=30; 3.1-4.1 years, mean age 3.5 years;  12 male, 18 female | **Direct life triaxial accelerometer (Tracmor_D_)***-*  *Middle of lower back*  *1 min epoch*  *Wear time:*  *Parents reported sleep and wake time, and times of wearing accelerometer. Day invalid when time during which accelerometers worn and time spend sleeping did not add up to at least 19 hours.*  *Valid n of days: At least 3 days of wear time* | Doubly labelled water (DLW) | 3-5 days of habitual activity, during waking hours apart from water activites *(free living)* | TracmorD Triaxial Accelerometer- Activity counts  DLW- TEE, AEE, PAL | **Pearson’s correlations:**  Activity counts per minute and per day were significantly correlated with PAL r = 0.61 (p= 0.004) and r = 0.46; p= 0.042) respectively.  Activity counts per minute correlated with activity EE r = 0.56 (p= 0.011), but no correlation between activity counts per day and activity EE was found r = 0.38 (p=0.098).  Activity counts per minute and per day were not correlated with TEE r = 0.34 (p= 0.094) and r = 0.21 (p =0.326) respectively.  **Linear regression:**  Activity counts per minute explained 31% of the variance in AEE (p=0.011). |
| **Proxy reported measurement tools (n=1)** | | | | | |
| **PA (n=1)** | | | | | |
| Corder et al. (2009) [68];  UK;  N=27; 4-5 years, mean age 4.9 years;  17 male, 10 female | **Children’s physical activity questionnaire (CPAQ)** | Doubly labelled water (DLW) | Questionnaire for past 7 days, remaining protocol (accelerometer and DLW) for 11 days assessing habitual activity *(free living)* | CPAQ- Physical activity energy expenditure (PAEE) and time spent in MVPA  Actigraph- Time spent in MVPA  DLW- PAEE | **Spearman’s correlations:**  PAEE assessed by CPAQ was not significantly correlated with PAEE from DLW r=0.22 (p=0.28)  **Bland Altman:**  Mean bias (PAEE) = -14.4 ±52.4 (95% CI -35.2, 6.3) |

**Abbreviations**: SB= sedentary behaviour; LPA= light physical activity; MVPA =moderate to vigorous physical activity; HR= heart rate; EE= energy expenditure; AEE =activity energy expenditure; TEE= total energy expenditure; VM= vector magnitude; PAL =physical activity level; MET = metabolic equivalent of task; ROC-AUC= area under the receiver operating curve; RMSE= Root mean squared error; Tracmor_D_= Direct life triaxial accelerometer; RT3= Triaxial Research Tracker 3; CPAQ= Children’s physical activity questionnaire
