# Supplementary material for: A systematic review of the validity, reliability, and feasibility of measurement tools used to assess the physical activity and sedentary behaviour of pre-school aged children
Source: Int J Behav Nutr Phys Act. 2021 Nov 4;18:141. doi: 10.1186/s12966-021-01132-9 (PMC8567581; doi:10.1186/s12966-021-01132-9)
Supplement: Supplementary file 5 — Additional file 5. Study details of level 2 validity evidence. [file 12966_2021_1132_MOESM5_ESM.docx]

**Additional File 5: Study details of level 2 validity studies (n=36, describing 45 comparisons)**

| Study details | Methods | | | Units of measure | Validity results |
| --- | --- | --- | --- | --- | --- |
|  | **Measurement tool(s) under study**  *Placement, epoch, cut points, wear time, non-wear time and number of valid days* | **Comparison Tool** | **Study protocol** *(lab/free living)* |  |  |
| **Direct observation (newly devised) (n=1)** | | | | | |
| **PA and SB (n=1)** | | | | | |
| Klesges et al. (1985) [72];  USA;  n=30; 3.5-6.5 years, mean age 4.8 years; 10 male, 20 female | **Fargo Activity Timesampling Survey (FATs)– Continuous observation.** | **Fargo Activity Timesampling Survey (FATs)– Time sampling** | Child observed for 1 hour during free play in nursery setting *(free living* | Behaviour (sleeping/lying down, crawling, climbing, standing, walking, running)  Intensity (minimal, moderate, extreme) | **Pearson’s correlations:**  Correlations between the 2 observation sampling methods (p<0.001). Total behaviour r=0.79, total intensity r=0.77 and total behaviour by intensity = r=0.90.  Correlations between the 2 sampling methods for all behaviours and intensities (p<0.001).  **T-tests:**  Continuous method yielded higher levels of crawling (t (28)= 2.87, p<0.001), walking (t (28)= 7.86, p<0.001), running (t (28)= 4.23,  p<.001), moderate levels of activity (t (28)= 4.16, p<0.001), and extreme levels of activity (t (28)= 3.26, p<0.01) than the time sampling method. Continuous method yielded lower levels of sitting (t (28)= 4.35, p<0.001) and minimal levels of activity (t (28)= 4.64, p<0.001) compared with the time sampling |
| **Combined heart rate and accelerometry and accelerometers (n=1)** | | | | | |
| **PA and SB (n=1)** | | | | | |
| Adolph et al. (2012) [57];  USA;  n=64; 3-5 years, mean age=4.5 years;  37 male, 27 female  50% White, 27% Black, 22% Hispanic, 2% Asian | **Actiheart (MiniMitter)**  *Chest*  *15 sec epoch*  **Actical (Respironics, MiniMitter)**  *Right hip*  *15 sec epoch*  **Triaxial Research Tracker 3 (RT3- Stayhealthy)**  *Right hip*  *1 sec epoch*  *Cut points: Piecewise linear regressions determined thresholds into SB, LPA, MPA, VPA* | Direct observation (CARS) | 3 hour whole room calorimeter protocol *(laboratory based)* | Accelerometers: activity counts per minute  Direct observation: Activity levels | **Pearson’s Correlation:**  In increasing order - Actical, Actiheart, RT3 total counts were positively correlated with AEE and physical activity ratings r=0.69-0.82 (p=0.001).  Similar correlations observed between accelerometer counts and CARS and HR r=0.64-0.80 (p=0.001).  **True positive predictive rates for MVPA:**  Actical- 69%  Actiheart – 82%  RT3- 79% |
| **Heart Rate Monitors (n=1)** | | | | | |
| **PA and SB (n=1)** | | | | | |
| Bar-Or et al. (1996) [73];  Canada;  n=27; 3-5 years, mean age=4.3 years;  18 male, 9 female | **Polar Vantage XL Monitor**  *Chest and wrist*  *Records every 5s* | Electrodiagram (ECG) | 19 minute structured activity protocol *(laboratory setting)* | HRM- Heart beats per minute  Electrodiagram- ECG recordings | **Pearson’s correlation:**  Correlations between ECG and heart rate monitor values ranged from r=0.92 to r=0.99  Values very similar for resting (ECG = 97.3 ±7.5, XL = 97.9 ±7.2 for supine and ECG = 111.1 ±16.5, XL = 110.4 ±16.3 for upright, both r=0.99). However, the XL monitor tended to underestimate heart rate during cycling (ECG = 142.7 ±11.0, XL = 140.2 ±11.5, r=0.93), and overestimate heart rate during recovery (ECG = 112.4 ±12.8, XL = 118.0 ±12.3, r=0.92). |
| **Accelerometers (n=26)** | | | | | |
| **PA and SB (n=19)** | | | | | |
| Sirard et al. (2005) [85];  USA;  N=269; 3-5 years, mean age not reported;  125 male, 144 female  3yr; 37.7% white, 4yr; 25.6% white, 5yr; 38.7% white | **Actigraph (MTI)**  *Right hip*  *15 sec epoch*  *Cut points: Sirard et al., 2005* | Direct observation (CARS) | Children wore Actigraph for the entire time they were at school for up to 10 consecutive weekdays, direct observation for 1-3 hours of activity in preschool *(free living)* | Actigraph – Activity counts to determine SB, TPA, LPA, MPA, VPA and MVPA  Direct observation –Activity levels (SB, LPA, MPA, VPA, MVPA. | **Pearson’s correlation:**  Significant (p<0.001) correlations between Actigraph and direct observation:  TPA r=0.58, SB r=0.70, LPA r=0.59, MPA r=0.50, MVPA r=0.46, VPA r=0.61. |
| Hands et al. (2006) [82];  Australia;  N=23; 5-6 years, mean age 5.5 years;  12 male, 11 female | **Actigraph (MTI AM7164)**  *Waist*  *10 sec epoch* | Direct observation (CARS) | Children monitored for 30 minutes over 5 days during free playing in their school setting *(free living)* | Actigraph- Activity counts  Direct observation- Activity levels | **Pearson’s correlations:**  Accelerometer and direct observation r=0.77 (p<0.01).  Accelerometer shared 59% of variance with direct observation.  **One way ANOVA:**  When the children were grouped into low, moderate, and high activity levels using observation, there was statistically significant differences between groups for the accelerometer. However data from a pedometer data was better able to separate the groups than from the accelerometer. |
| Dobell et al. (2019) [75];  UK;  N=62; 3-4 years, mean age 3.5 years;  36 males, 30 females (sex of participants prior to exclusion of 4 data points) | **Actigraph (GT3X)**  *Right hip and non-dominant wrist*  *5, 10, 15 and 30 sec epochs*  *Cut points: produced during ROC curve analysis.* | Direct observation (OSRAC-P) | 60 minutes of activity in pre-school setting *(free living)* | Actigraph- Raw acceleration and activity counts to determine SB, LPA, MVPA  Direct observation – Activity levels | **Spearman’s correlation:**  Correlations between accelerometer raw counts and observations were weak to moderate (ranging from r=0.34 to r=0.55, all p<0.05).  **Area under the receiver operating characteristic:**  Agreement for accelerometer counts and observation levels for both SB and MVPA (AUC ≥0.7).  Agreement between observation and accelerometer for LPA ROC = 0.53 to 0.56.  Hip and wrist placement resulted in similar levels of agreement with the wrist performing marginally better for SB and LPA, and the hip performing better for MVPA. |
| Hislop et al. (2016) [74];  Scotland, UK;  N=32; 3-5 years, mean age 4.2 years;  21 male, 11 female | **Actigraph (GT3X+)**  *Non-dominant wrist and hip*  *1 sec epoch*  *Cut points: Johansson et al., 2015 for wrist data and Evenson et al., 2008 for hip data* | Direct observation (CARS) | Children wore accelerometers whilst being video recorded for 60 minute activity in pre-school *(free living)* | Actigraph- activity counts to determine minutes in SB, LPA, MVPA and TPA  Direct observation- Activity levels | **Bland Altman:**  Agreement between CARS and actigraph (mean minutes):  SB:  CARS 6.2(5.8); Actigraph wrist 7.2 (3.9) and Actigraph hip 12.2 (7.2).  LPA:  CARS 27.3(10.6); Actigraph wrist 16.9 (6.6) and Actigraph hip 17.2(6.6).  MVPA:  CARS 2.2 (2.2); Actigraph wrist 11.5 (6.3) and Actigraph hip 6.1 (4.5)  TPA:  CARS 29.5(10.4); Actigraph wrist 28.4(9.8) and Actigraph hip 23.3 (9.8).  Mean difference and 95% LOA:  Standing and LPA:  Actigraph wrist (3.1, -14.3, 19.9)  Actigraph hip (2.8, -10.3, 16.4)  MVPA:  Actigraph wrist (-9.3, -20.0, 1.5)  Actigraph hip (-3.8, -10.7, 3.1)  TPA:  Actigraph wrist (1.1, -9.9, 12.1)  Actigraph hip (6.3, -8.8, 21.4)  Large differences in the count data from the hip and wrist placements. |
| Kahan et al (2013) –  Study 1 [77]:  USA; n=57; 4-5 years, 4.7 years,  25 male, 32 female  70.2% White, 12/3% Asian/Pacific Islander, 10.5% Black/African American,  7% Hispanic/Latino. | **Actigraph (GT3X)**  *Right hip*  *15 sec epoch*  *Cut points: Evenson et al., 2008, Pate et al., 2006, Sirard et al., 2005 and Van Cauwenberghe et al., 2011* | Direct observation (OSRAC-P) | At least 15 minutes of activity in pre-school *(free living)* | Actigraph- % time spent in SB and MVPA determined from activity counts  Direct observation- Activity intensity levels | **Z-test:**  SB- Sirard followed by Van Cauwenberghe cut points most closely matched OSRAC-P estimates; both overestimated SB by less than 9%.  MVPA- Sirard followed by Van Cauwenberghe and Evenson cut points most closely matched OSRAC-P estimates. Sirard cut points underestimated and Van Cauwenberghe and Evenson cut points overestimated OSRAC-P estimates by less than 5% and by about 10% respectively.  **Bland Altman:**  Biases between OSRAC-P and Actigraph Sirard cut points were -8.4% ±18.3% (SB) and 5.7% ±9.7% (MVPA). Limits of agreement were -45.0% to 28.1% (SB) and -13.7% to 25.1% (MVPA). Comparatively less was observed when SB means exceeded about 70%. For MVPA, the greatest clustering around zero difference was observed when mean MVPA was very low. |
| Kahan et al (2013)-  Study 2 [77]:  USA; n=12; 4-5 years, mean age not reported;  3 male, 9 female | **Actigraph (GT3X)**  *Right hip*  *5 sec epoch*  *Cut points: Evenson et al., 2008, Pate et al., 2006, Sirard et al., 2005 and Van Cauwenberghe et al., 2011* | Direct observation (OSRAC-P) | At least 15 minutes of activity in pre-school *(free living)* | Actigraph- % time spent in SB and MVPA determined from activity counts  Direct observation- Activity intensity levels | **Kappa coefficients:**  Sirard (κ = 0.48) and Van Cauwenberghe (κ = 0.48) cut points showed lowest kappa values, and Evenson (κ = 0.58) showed highest.  **Sensitivity:**  SB: Evenson – 75.2%; Pate- 50%; Sirard- 81.4%; Van Cauwenberghe- 80%  MVPA: Evenson –54.8%; Pate-62.7%; Sirard-35.8%; Van Cauwenberghe- 53.9%  Across cut points, sensitivity was higher for SB than for MVPA, for all except Pate cut points.  **Specificity:**  SB: Evenson – 66.4%; Pate- 85.7%; Sirard- 50.7%; Van Cauwenberghe- 52.4%  MVPA: Evenson –78.9%; Pate-72.2%; Sirard-87.6%; Van Cauwenberghe- 79.1%  Across cut points, specificity was higher for MVPA than for SB, for all except Pate cutpoints.  **Positive Predictive Value:**  SB: Evenson – 46.1%; Pate- 74.2%; Sirard- 57.5%; Van Cauwenberghe- 58%  MVPA: Evenson –42.1%; Pate-62.8%  Sirard-44.8%; Van Cauwenberghe- 40.3%  Positive predictive value was higher for SB than for MVPA.  **Negative Predictive Value:**  SB: Evenson – 87.6%; Pate- 67.6%; Sirard- 76.9%; Van Cauwenberghe- 76.1%  MVPA: Evenson –87%; Pate-88%; Sirard-84%; Van Cauwenberghe- 86.7%  Negative predictive value was higher for MVPA than SB, except for Evenson.  **Accuracy:**  SB: Evenson – 68.9%; Pate- 69.6%; Sirard- 64.5%; Van Cauwenberghe- 64.8%  MVPA: Evenson –73.9%; Pate-73%; Sirard-77.5%; Van Cauwenberghe- 73.8%  Accuracy was higher for MVPA than SB across all cut points. Sirard and Pate cut points were most accurate for MVPA and SB, respectively. |
| Janssen et al. (2013a) [61];  Australia  N=40; 4-6 years, mean age 5.3 years;  22 male, 18 female. | **Actigraph (GT3X)**  *Right hip*  *15 sec epoch*  *Cut points: Activity energy expenditure and* VO_2_*: Puyau et al., 2002, Pate et al., 2006; and activity intensity:*  *Puyau et al., 2002, Pate et al., 2006, Evenson et al., 2008, Sirard et al., 2005, Van Cauwenberghe et al., 2011 and Reilly et al., 2003* | Direct observation (CARS) | 150 minute structured activity laboratory protocol *(lab based)* | Actigraph- Activity counts converted to activity energy expenditure, VO_2_ and activity intensity (SB, LPA, MVPA).  Direct observation- Activity intensity: SB, LPA, MVPA. | **Classification accuracy determined using ROC-AUC, sensitivity, specificity:**  **PA intensity-**  Using direct observation alone as the criterion measure:  SB: Classification accuracy significantly higher for Evenson cut point (ROC-AUC = 0.80, sensitivity = 86.7%, specificity= 72.9%) compared to all other cut points (P<0.05).  LPA: Classification accuracy was significantly higher for Evenson cut point (ROC-AUC = 0.65, sensitivity = 54.8%, specificity= 74.8%) compared to all other cut points (P<0.05).  MVPA: using the Pate cut point (ROC-AUC = 0.72, sensitivity = 54.2%, specificity= 88.9%) cut-point resulted in classification accuracy which was significantly higher compared to all other cut points (P<0.05).  **Both PA intensity and EE:**  Classification accuracy for Evenson cut point for SB (ROC-AUC = 0.90, sensitivity = 90.7%, specificity= 89.7%), LPA (ROC-AUC = 0.76, sensitivity = 69.9%, specificity= 82.2%) and MVPA (ROC-AUC = 0.76, sensitivity = 60.5%, specificity= 91%); higher accuracy compared to all others expect Pate cut point for MVPA (ROC-AUC = 0.78, sensitivity = 69.6%, specificity= 86.4%). |
| Hislop et al.(2012a) [79];  Scotland, UK;  N=31; 3-5 years, mean age 4.4 years;  15 male, 16 female | **Actigraph (GT1M)**  *Waist*  *1 sec epoch*  *Cut points: SB, LPA, MVPA -Puyau et al.,2002, Van Cauwenberghe et al., 2011, Sirard et al., 2005; MVPA only- Pate et al., 2006; SB only – Reilly et al., 2003, Evenson et al., 2008* | Direct observation (CARS) | 60 minutes activity in pre-school setting *(free living)* | Actigraph- SB, LPA, MVPA  Direct observation- Activity levels | **Friedman’s repeated measures ANOVA, followed by Wilcoxon paired t-tests:**  Significant difference between the mean time in SB, LPA and MVPA estimated by the different cut points and the CARS (p=0.00).  Actigraph compared with direct observation categories, using the different cut points:  Puyau 🡪 No significant difference between SB (p=0.8); LPA (p=0.06), MVPA (p=0.06) and CARS.  Sirard🡪 No significant difference for MVPA and CARS (p=0.2). Significant difference for SB and LPA and CARS (p<0.01).  Van Cauwenberghe🡪Significant difference for SB, LPA and MVPA, with CARS (p<0.01).  Pate 🡪Significant difference for MVPA and CARS (p<.01).  Reilly and Evenson🡪Significant difference for SB and CARS (p<0.01).  **Bland Altman:**  SB- There was a bias towards a greater difference in estimation of time spent in SB between accelerometer and CARS when using Sirard, Van Cauwenberghe and Reilly cut points, lower mean difference in time in SB for the Evenson cut points, Puyau SB cut point were close to 0, but had wide limits of agreement (13.2 to 14.0mins).  Sirard (-7.2; -20.2,5.7)  Puyau (0.4; -13.25, 13.99)  Van Cauwenberghe (-8.2; -22.7,6.2)  Evenson (13.2; -2.2,28.6)  Reilly (-3.6;-17.6,10.4)  LPA-All cut points had lower estimation of time spent in LPA in relation to CARS.  Sirard (6.3; -6.0,18.6)  Puyau (-2.2; -15.0,10.5)  Van Cauwenberghe (11.4; 1.4,24.2)  MVPA- Positive bias, with underestimation in MVPA when compared with CARS when using Sirard and Puyau cut points, overestimation when using Van Cauwenberghe and Pate cut points.  Sirard (0.8; -6.2,7.8)  Puyau (1.7; -8.0, 11.5)  Van Cauwenberghe (-3.3; -11.8,5.3)  Pate (-8.7; -19.9,2.5) |
| Alhassan et al. (2017) [76];  USA;  N=33; mean age 4.4 years; 21 male, 12 female | **Actiwatch(Spectrum)**  *Non dominant wrist*  *15 sec epoch*  *Cut points: Ekblom et al., 2012*  **Actigraph (GT3X)**  *Waist, centre of lower back*  *15 sec epoch*  *Cut points: Pate et al., 2006 and Sirard et al., 2006* | Direct observation (OSRAC-P) | 30 minutes of daily activity in pre-school *(free living)* | Accelerometers- activity counts to determine SB, LPA, MPA, VPA, MVPA  Direct observation- Activity intensity categories | **Spearman’s correlation:**  Association between direct observation intensity categories and the accelerometers r=0.47.  **Wilcoxon rank sum:**  Actiwatch and Actigraph able to differentiate between median values for adjacent direct observation intensity levels - All comparisons between accelerometers and direct observation intensity categories significant (p=0.03).  **Bland Altman:**  Show that both Actiwatch and Actigraph similarly underestimate mins of MVPA compared with direct observation.  **% Agreement between devices and direct observation:**  SB: Actiwatch = 65.4%; Actigraph = 61.5-62.7%  LPA: Actiwatch = 53.8%; Actigraph = 60.7-65%  MPA: Actiwatch = 82.8%; Actigraph= 80.3-86.3%  VPA: Actiwatch = 86.3%; Actigraph = 86.1-86.5%  MVPA: Actiwatch = 77.5%; Actigraph = 75.3-76.9% |
| Kelly et al. (2004) [83];  UK;  n=78; 3-4 year olds, mean age 3.5 years;  30 male, 48 female | **Actiwatch (AW16)**  *Right hip*  *1 min epoch*  **Actigraph (CSA/MTI)**  *Right hip*  *1 min epoch* | Direct observation (CPAF) | 39-45 minutes structured play class *(usual activity)* | Accelerometers- Activity counts per minute  Direct observation- Activity levels | **Spearman’s correlations:**  Actiwatch not significantly correlated with direct observation r =0.16.  Actigraph significantly correlated with direct observation r=0.72 (p <0.001).  **Pearsons’s correlation:**  Within child significant positive correlations between Actigraph and direct observation r =0.52 (p <0.01; range –0.22 to +0.87), and for Actiwatch and direct observation r=0.55 (p <0.01; range –0.41 to +0.88). |
| Finn & Specker (2000) [93];  USA;  n=40; 3-4 years, mean age not reported;  16 male, 24 female  95% Caucasian | **Actiwatch (MinMitter)**  *Waist lower back*  *1 min epoch* | Direct observation (CARS) | 5-6 hours activity in child care setting *(free living)* | Actiwatch- Activity counts per minute  Direct Observation- Activity level | **Correlations:**  3 min CARS scores with 3 min activity counts had a median correlation coefficient of r=0.74 (range 0.03 to 0.92).  **Mixed model repeated measures:**  Positive relationship between within-child correlation coefficients and the mean 3 min CARS r=0.37 (p=0.02) or mean activity counts r=0.31 (p=0.05). |
| Djafarian et al. (2013) [94];  Scotland, UK;  N=42; 3-5 years, mean age 4.06 years;  22 male, 20 female  100% Caucasian | **Actiwatch-L**  *Non dominant wrist*  *1 min epoch* | Direct observation (CARS) | 2 hours usual structured and unstructured activity in preschool setting *(usual activity, not free living)* | Actiwatch-L : Activity counts per minute  Direct observation: Activity level | **Correlations:**  Correlation between Actiwatch-L counts and CARS scores for varying intensities, range from r=0.41 to r=0.63 (p<0.001). |
| Davies et al. (2012) [88];  UK;  n=32; 3.1-4.9 years, mean age 4.1 years;  11 male, 21 female | **ActivPAL**  *Thigh*  *1 sec epoch* | Direct observation (not specific tool) | 1 hour activity in nursery setting *(free living)* | Time spent in the different postures (posture allocation): Sit/ lie, stand, walk | **Paired t-test:**  ActivPAL underestimated total time spent sitting (mean difference -4.4%, p<0.01) and overestimated time standing (mean difference 7.1%, p<0.01). No difference in overall % time categorised as walk (p=0.2).  **Bland Altman:**  Direct observation with ActivPAL output showed that bias was not associated with the amount of time detected in each category (r=-0.17, p=0.4 for sit/lie; r=-0.03, p=0.9 stand).  **Sensitivity, specificity, positive predictive value (PPV):**  Sit/lie validation: individual, sensitivity = 92.8%; specificity = 97.3% and PPV = 97%. Group, specificity = 99.5% and PPV = 99.4%  -Stand validation: individual, sensitivity = 91.8%; specificity = 86.5% and PPV = 70.4%. Group, specificity = 87.9% and PPV = 72.4%  -Walk validation: individual, sensitivity = 77.9%; specificity = 96.5% and PPV = 73.4%. Group, specificity = 96.7% and PPV = 77.6% |
| Janssen et al. (2013b) [89];  Australia;  n=38; 4-6 years, mean age 5.3 years;  20 male, 18 female | **ActivPAL**  *Thigh*  *1 sec epoch* | Direct observation (not specific tool) | 150 minute protocol *(laboratory based)* | Posture allocation (*sit/lie; stand; walk)* | **Classification accuracy determined by ROC-AUC, sensitivity and specificity:**  Sit/Lie: ROC-AUC = 0.84-0.88 (including and excluding ‘other’ postures respectively). Classification accuracy increased significantly when ‘other’ postures were excluded (p<0.05).  Standing: ROC-AUC= 0.76-0.77 (including and excluding other postures respectively).  Walking: ROC-AUC = 0.73-0.74 (including and excluding other postures respectively).  **Dependent samples t-test:**  Differences between time spent in postures between ActivPAL and direct observation:  Mean difference and 95% confidence intervals between the methods (ActivPAL-observation) were:  Sit/Lie: +5.9% (0.6% to 11.1%)  Stand: +14.8% (11.6% to 17.9%)  Walk: -10.0% (-12.9% to -7.0%)  No significant difference was found between time spent in sit/lie for activPAL and direct observation (p=0.58).  Significant difference between time spent in stand/walk for activPAL and direct observation (p<0.05). |
| Janssen et al. (2014) [65];  Australia;  N=18; 4-6 years, mean age 5.2 years;  9 male, 9 female | **ActivPAL**  *Thigh*  *15 sec epoch*  *Cut points: produced during ROC curve analysis* | Direct Observation (CARS) | 150 minute room calorimeter protocol *(laboratory based)* | ActivPAL- activity counts to determine MVPA and METs equation  Direct observation- Activity levels | **Classification accuracy determined by ROC-AUC, sensitivity, specificity:**  ActivPAL METs equation overestimated METs during SB (+6.0%) and underestimated METs for LPA (-15.3%), MVPA (-32.8%) ad total METs (-13.6%) (all p<0.001).  Classification accuracy for activPAL determined MVPA and direct observation: sensitivity = 88.3%, specificity = 88.2%, ROC-AUC =0.88.  Classification accuracy for activPAL determined MVPA when using both EE and direct observation as criterion: Sensitivity = 94.8%, Specificity = 84.8%, ROC-AUC = 0.90. |
| Ettienne et al. (2016) [86];  USA;  n=30; Mean age =3.5 years;  17 male, 13 female  46% Native Hawaiian, 14% Other Pacific Islander-  All of mixed ethnicities.  Children recruited from Head Start sites | **Actical (Respironics, Philips)**  *Non dominant wrist*  *15 sec epoch*  *Cut points: Schaefer et al., 2014*  *Non-wear time: >1300 minutes of SB per day.* | Direct observation (SOFIT-P) | 7 days habitual activity examined by accelerometer, up to 3 hours of observation during pre-school hours *(free living)* | Actical- activity counts to determine activity intensity- SB, LPA, MPA, VPA.  Direct observation – activity intensity categories | **Cohen’s Kappa:**  Proportion of agreement between Actical and DO =74%, weighed kappa coefficient was 0.17 (p<0.001). |
| Janssen et al. (2015) [62];  Australia;  N=40, 4-6 years, mean age 5.3 years;  22 male, 18 female | **Actical (Respironics, Philips)**  *Right hip*  *15 and 60 sec epoch*  *Cut points: Pfeiffer et al., 2006, Adolph et al., 2012 and Evenson et al., 2008* | Direct observation (CARS) | 150 minute room calorimeter protocol *(laboratory based)* | Actical- AEE; activity intensity - SB, LPA, MVPA  Direct observation- PA Intensity categories | Actical compared with DO:  **Kappa:**  Adolph (60s): (κ = 0.64, 95% CI = 0.64–0.65) exhibited substantial agreement.  Adolph(15s): (κ = 0.58, 95% CI = 0.57–0.58) and Evenson (κ = 0.52, 95% CI = 0.52–0.53) exhibited moderate agreement for predicting PA and SB determined by DO.  **Classification accuracy determined by ROC-AUC, sensitivity and specificity :**  Broken down by intensity:  SB: Adolph (15s): ROC-AUC=0.80, sensitivity = 89.1%, specificity= 71.4%  Adolph (60s): ROC-AUC=0.82, sensitivity = 82.5%, specificity=81.1%  Evenson (15s): ROC-AUC=0.79, sensitivity = 91%, specificity= 68%  Classiﬁcation accuracy was signiﬁcantly higher for Adolph compared to Evenson (P < 0.05).  LPA: Adolph (15s): ROC-AUC=0.68, sensitivity = 51.9%, specificity= 84.3%  Adolph (60s): ROC-AUC=0.73, sensitivity = 66.2%, specificity=80.2%  Evenson (15s): ROC-AUC=0.65, sensitivity = 51%, specificity= 79.1%  Adolph showed a signiﬁcantly higher sensitivity (66.2%), which resulted in signiﬁcantly higher classiﬁcation accuracy compared to Evenson (P < 0.05).  MVPA: Adolph (15s): ROC-AUC=0.82, sensitivity = 71%, specificity= 93.6%  Adolph (60s): ROC-AUC=0.85 sensitivity = 74.3%, specificity=95.3%  Evenson (15s): ROC-AUC=0.75, sensitivity = 54.9%, specificity= 96%  Pfeiffer (15s): ROC-AUC=0.70, sensitivity = 43.9%, specificity= 96.5%  Classiﬁcation accuracy was signiﬁcantly higher for Adolph compared to all others (p< 0.05).  Actical compared with direct observation and EE:  **Kappa:**  κ values were slightly increased compared to using direct observation only. Adolph (κ = 0.72, 95% CI = 0.70–0.74), Adolph(15s) (κ = 0.65, 95% CI = 0.64–0.65) and Evenson (κ = 0.61 95% CI = 0.60–0.62) exhibited substantial agreement predicting PA and SB determined by DO and calorimetry.  **Classification accuracy determined by ROC-AUC, sensitivity and specificity :**  ROC-AUC values when using direct observation combined with EE were slightly higher but similar to using direct observation only.  Adolph was signiﬁcantly better than all others (P < 0.05) when classifying LPA and MVPA. For SB, classiﬁcation accuracy was good for the Adolph and Evenson (ROC-AUC = 0.82–0.85) with Adolph performing signiﬁcantly better than Evenson. However, when comparing Evenson and Adolph(15s) this difference disappeared. |
| Byun et al. (2018a) [90];  USA;  n=28; 3-5 years, mean age 4.8 years;  15 male, 13 female | **Fitbit (Flex 1)**  *Non-dominant wrist*  *60 sec epoch* | Direct observation (not a specific tool) | Children performed a set of unstructured and structured activites of different intensities for 34 minutes *(free living)* | Fitbit - Activity counts to define activity intensities: SB, LPA, MPA, VPA  Direct observation- activity intensity score: SB, LPA, MPA, VPA | **Spearman’s rank correlation:**  Correlations between direct observation and Fitbit were statistically significant for SB r=0.81 (p<0.05), MVPA r=0.62 (p<0.05), and TPA r=0.81 (p<0.05), but not for LPA r=0.21.  **Relative agreement (in minutes):**  Fitbit recorded higher minutes of SB (absolute mean difference = 2.3mins), and LPA (absolute mean difference = 4.6mins), but lower mins of MVPA (absolute mean difference =6.9mins) and total PA (absolute mean difference =2.3mins).  **Mean absolute % error:**  Lower for SB (28.8%) and TPA (11.5%), than for MVPA (46%) and LPA (92%).  **Kappa:**  Levels of agreement determined by the kappa statistic for SB (*k=0*.78) and TPA (*k=0*.78), weaker for LPA (*k=0.*18) and MVPA (*k=0*.51).  **ROC-AUC, sensitivity, specificity:**  SB: ROC-AUC: = 0.92; Sensitivity= 96.8%; Specificity=88.6%  LPA: ROC-AUC = 0.63; Sensitivity= 55%; Specificity=70%  MVPA: ROC-AUC = 0.77; Sensitivity= 53.3%; Specificity=99.4%  TPA: ROC-AUC = 0.92; Sensitivity= 88.6%; Specificity=96.8% |
| Saris & Binkhorst (1977) [137];  Netherlands;  n=4 ; 4.8-6.1 years; mean age and sex not reported | **Actometer**  *Right ankle and wrist* | Direct observation (not specified) | Children engaged in free play activity in their pre-school setting *(free living)* | Actometer units  Direct observation - Activity category (sitting, standing, walking, running) and intensity (high, low) | **Correlations:**  Significant correlations between: Direct observation and actometer ankle r=0.97 (p<0.01),  actometer wrist r=0.71 (p<0.05), actometer total r=0.97 (p<0.01). |
| **PA (n=4)** | | | | | |
| Liggett et al. (2012) [92];  New Zealand;  N=14; 3-4 years old, mean age 3.9 years;  7 male, 7 female. | **New Lifestyles NL-1000**  *Right side of waistband*  *4 sec epoch*  *Cut points: Internal categorisation* | Direct observation (CARS) | 15-20 minutes activity at pre-school *(usual activity-children stopped every few minutes so researcher could check device)* | New Lifestyles NL-1000-MVPA  Direct observation- Activity intensity level | **Linear mixed model:**  Clear linear association between CARS and NL-1000. Most of the variation in CARS (95.4%, with 95% CI 92.6–97.5) could be explained by variation in the NL-1000 measurements, with 2.4% of the variation being participant-specific. |
| Fairweather et al. (1999) [81];  Scotland, UK;  n=11; mean age 3.7 years;  3 male, 8 female | **Actigraph (CSA 7164)**  *Right hip*  *1 min epoch*  *Cut points: not specified* | Direct observation (CPAF) | 40-50 minute structured play session *(usual activity)* | Actigraph- Counts per minute  Direct observation- Activity level | **Spearman’s correlation:**  Rank order correlation between activity as determined by CSA and CPAF was high and statistically significant r=0.79 (p<0.01).  **Linear correlation:**  Mean CSA counts per min significantly correlated with mean CPAF counts per min r=0.87 (p<0.01). |
| Hislop et al. (2012b) [80];  Scotland, UK;  N=31; 3-5 years, mean age 4.4 years;  15 males, 16 females | **Actigraph (GT1M)**  *Right hip*  *1 sec epoch*  *Cut points: Sirard et al., 2005, Freedson et al., 2005, Pate et al., 2006, Evenson et al.., 2008, Van Cauwenberghe et al., 2011, Puyau et al., 2002*  **RT3 (Stayhealthy)**  *Right hip*  *1 sec epoch*  *Cut points: Vanhelst et al., 2011, Rowlands et al.,2004; Sun et al., 2008, Chu et al.,2007* | Direct observation (CARS) | 60 min free play in nursery setting (free living) | Accelerometers- Activity counts, MVPA  Direct observation- MVPA | **Spearman’s correlation:**  Significant correlation between counts per minute and % of time spent in MVPA as recorded by direct observation for the GT1M r=0.56 (p<0.01) and for the RT3 r=0.39 (p<0.03).  **Bland Altman:**  Mean difference between number of minutes of MVPA recorded by GT1M and direct observation was 0.8 min (15s epoch) and 3.2 min (60s epoch).  Mean difference for RT3 (walking) was 12.2 min (15s epoch) and 13.6 min (60s epoch) and for the RT3 (light jog) was 0 min (15s epoch) and 1.7 (60 s epoch). |
| Sharp et al. (2017) [91];  North Wales, UK;  N=56; 3-4 years, mean age 3.7 years;  29 female, 27 male | **FitBit (Zip)**  *Both right hip* | Direct observation (not specific tool) | 5 min structured walking task *(nursery setting, not free living)* | Step counts | **Bland Altman:**  Bias between observer counts with identified steps from devices :  Fitbit 1: 22.8±19.1 (-14.7 to 60.2) steps and  Fitbit 2:  25.2±23.2 (-20.2 to 70.5) steps.  **Absolute percent error:**  Fitbit 1: 6.44% (SE=0.66)  Fitbit 2: 7.27% (SE=0.94)  The majority of Fitbit 1 and Fitbit 2 step counts were in the exact-counting range (80.40% and 76.70%, respectively), with the remaining in the undercounting range. |
| **SB (n=3)** | | | | | |
| Reilly et al. (2003) [84];  Scotland, UK;  N=52; 3-4 years, mean age 3.5 years;  21 male, 31 female | **Actigraph (CSA/MTI AM7164)**  *Right hip*  *1 min epoch*  *Cut point: Reilly et al., 2003* | Direct observation (CPAF) | *40 minutes of free play activity in nursery setting (free living)* | *Actigraph- Activity counts*  *CPAF scores - Activity level* | **Kruskal Wallis followed by Mann Whitney-U tests:**  Median accelerometer counts per minute differed significantly between CPAF categories 1 to 4 (p <0.001). However, accelerometer output counts did overlap between categories. |
| De Decker et al. (2013) [78];  Belgium;  n=44; mean age 5.5 years; 22 male, 22 female | **ActivPAL**  *Thigh*  *15 sec epoch*  **Actigraph (GT1M)** *Right hip*  *15 sec epoch*  *Cut points: Evenson et al., 2008*  **Both devices-**  *Wear time: First day of data deleted, minimum of 6 hrs per day required ( this included data between 7am and 9pm on weekdays, 8am and 8pm on weekend days)*  *Non wear time:*  *≥10 min of consecutive zero activity counts.*  *Valid n of days: 3 days required to be included* | Direct observation (not specific tool) | Monitors to be worn for 5 consecutive days whilst being directly observed for 1 hour during pre-school time *(free living)* | ActivPAL: Posture allocation, time spent in: sit/ lie, stand or stepping.  Actigraph: SB or non-sedentary behaviour  Direct observation: SB or non-sedentary behaviour | **ROC-AUC, sensitivity and specificity:**  Sitting/Lying:  ActivPAL: ROC-AUC : 0.61; Sensitivity = 53.8%; Specificity = 67.5%.  Actigraph: ROC-AUC: 0.60; Sensitivity = 58.5; Specificity = 61.2%.  Sitting/Lying/Standing:  ActivPAL: ROC-AUC : 0.52; Sensitivity = 27.8%; Specificity = 75.8%  Actigraph: ROC-AUC : 0.61; Sensitivity = 46.3%; Specificity = 75.8% |
| Alghaeed et al. (2013) [87];  Scotland, UK;  N=30; mean age 4.1 years; 10 male, 20 female | **ActivPAL**  *Thigh*  *1, 2, 5, 10 sec epochs* | Direct observation (not specific tool) | 1 hour during usual activity in nursery *(free living)* | *Sitting bouts* | **Bland Altman:**  2s minimum sitting upright position (MSUP): minimised bias and showed no significant difference relative to DO (limits of agreement -14 to +17 bouts per hr, mean difference 1.83, p=0.02)  5s MSUP: underestimated number of sitting bouts as measured by DO (limits of agreement -23 to 8, mean difference -7.27, p=0.001.)  10s MSUP: underestimated number of sitting bouts as measured by DO (limits of agreement -29 to 4, mean difference -12.57, p=0.001)  - 90% of sitting bouts were identical between activPAL and direct observation when using 2s MSUP. 5s and 10s underestimated number of sitting bouts. |
| **Pedometers (n=8)** | | | | | |
| **PA and SB (n=4)** | | | | | |
| McKee et al. (2005) [96];  Northern Ireland, UK;  N=30; 3-4 years, mean age not reported;  13 male, 17 female | **Yamax Digiwalker (SW-200)**  *Waist*  *3 min sampling period* | Direct observation (CARS) | 1 hour during usual activity during school time *(free living)* | Yamax Digiwalker SW-200 - step counts  Direct observation: Physical activity level | **Correlations:**  Correlation between the two methods: r= 0.86.  **Multilevel linear regression:**  CARS predicted an estimate of 88.60 (±2.48) of pedometer step counts. |
| Hands et al. (2006) [82];  Australia;  N=23; 5-6 years, mean age 5.5 years;  12 male, 11 female | **Yamax Digiwalker SW-200**  *Waist* | Direct observation (CARS) | Children monitored for 30 minutes over 5 days during free playing in their school setting *(free living)* | Yamax Digiwalker SW-200- Step count  Direct observation- Activity levels | **Pearson’s correlations:**  Pedometer and direct observation r=0.90 (p<0.01).  Pedometer shared 81% of variance with direct observation  **One way ANOVA:**  When the children were grouped into low, moderate, and high activity levels using observation, there was statistically significant differences between groups for the pedometer, with the pedometer data better able to separate the groups than accelerometer data. |
| Nishikido et al. (1982) [98];  Japan;  N=49; 5-6 years, mean age not reported;  25 male, 24 female | **Yamasa AM-5 Pedometer**  *Hip* | Direct observation (not specific tool) | 2 days of children engaging in usual activities in pre-school setting *(free living)* | Yamasa AM-5 Pedometer- Step count  Direct Observation- ‘activity appearance rate’ - running, walking, standing and sitting. | **Pearson’s correlation:**  Correlation coefficients between step rate and 1) running activity r=0.69 (p<0.001), r= 0.83 (p<0.001); 2) walking r= 0.36 (p<0.05), r=0.42, (p<0.05 ); 3) standing r=-0.37 (p<0.05), r=-0.21 (ns) and; 4) sitting r=-0.50 (p<0.01), r= -0.77 (p<0.001), in school 1 and 2 respectively. |
| Saris & Binkhorst (1977) [137];  Netherlands;  N=11; 4.8-6.1 years, mean age and sex not reported | **Pedometer (type not specified)**  *Right side of waist* | Direct observation (not specified) | Children engaged in free play activity in their pre-school setting *(free living)* | Pedometer units  Direct observation - Activity category (sitting, standing, walking, running) and intensity (high, low) | **Correlations:**  Significant correlations between direct observation and pedometer r=0.93(p<0.01). |
| **PA (n=4)** | | | | | |
| Louie & Chan (2003) [95];  Hong Kong;  N=145; 3-5 years, mean age 4.2 years;  84 male, 61 female | **Yamax Digiwalker (SW-200)**  *Right hip (some invited to wear on both right and left hip)* | Direct observation (CARS) | 25 minute physical activity class in normal school day *(free living)* | Yamax Digiwalker SW-200- step count  Direct observation-Activity level | **Pearson’s correlation:**  Significant correlation was found between the total pedometer counts and CARS scores r = 0.64 (p < 0.01). |
| Oliver et al. (2007) [97];  New Zealand;  N=13; 3-4.8 years, mean age 4.2 years;  7 male, 6 female | **Yamax Digiwalker (SW-200)**  *Left hip or right hip (at anterior superior iliac spine), randomly assigned* | Direct observation (CARS) | 35 minutes free-living activity *(crèche setting, not free living)* | Yamax Digiwalker SW-200- step count  Direct observation-Activity level | **Spearman’s correlation:**  Significant correlation between direct observation and pedometry r=0.59 (p = 0.04).  Wide 95% prediction intervals  for the pedometer data against CARS. |
| Fotini et al. (2015) [136];  Greece;  N=45; mean age 5.5 years; sex not reported | **Omron Walking Style Pro Pedometer (HJ-720IT-E2) and the Walking Style II (HJ-113)**  *Right and left hip, right and left front trouser pocket, back.* | Direct observation- Actual number of steps using hand counter (Basch SJ-504). | Treadmill walking and track walking *(structured protocols)* | Step count | **Standard error of measurement:**  Pedometers underestimated actual number of steps for treadmill walking by -0.5% to -38%. Accuracy improved at higher speeds.  Pedometers accurate (-3.5% to -0.6%) in measuring steps on over ground walking test.  Back and right or left hip placements yielded most accurate information. |
| Murray (2009) [99];  USA;  N=75; 3-5 years, mean age 4.4 years;  31 male, 44 female  34.3% African American,  64.4% Hispanic,  1.4% Native American  Children recruited from Head Start Centres | **Pedometer- MVP 4 Walk4Life Digital** *Right or left hip* | Direct Observation (SOFIT-P) | Children were observed in pre-school for 4 consecutive minutes or 12 observations whilst wearing pedometer at 2 time points *(free living)* | MVP 4 Walk4Life Digital Pedometer– step count, MVPA, activity time  Direct observation- Activity level, type and context  Results presented as: step counts, MVPA, activity time*.* | **Pearson’s correlations:**  No significant correlations at pre-test r = 0.06 (*p* = 0.77) and post-test r =0.30 (*p* =0.36) |
| **Proxy reported measurement tools (n=2)** | | | | | |
| **PA and SB (n=1)** | | | | | |
| Nishikido et al. (1982) [98];  Japan;  n=49; 5-6 years, mean age not reported;  25 male, 24 female | **Proxy report of children’s Habitual Physical activity (Questionnaires to mothers and teachers)** | Direct observation (not a specific tool) | Questionnaire assesses habitual activity. Remaining protocol for 2 days of children engaging in usual activities in pre-school setting *(free living)* | Proxy report: Children rated as: Inactive; relatively inactive; medial; relatively active; active.  Direct Observation: ‘activity appearance rate’ - running, walking, standing and sitting. | **Kendall’s rank order correlation:**  Teacher’s evaluations significant correlations with direct observation running activity appearance rate r=0.27 (p<0.01) and significant negative correlations with direct observation sitting activity r=-0.19 (p<.05). No other significant correlations. |
| **PA (n=1)** | | | | | |
| Noland et al. (1990) [100];  USA;  n=21; 3.8-5.6 years, mean age 4.7 years;  11 male, 10 female  90% White, 10% Black  Families were primarily middle and upper class. | **Parent and teacher report** | Direct observation (CARS) | 20 minute free play in school setting*,* sub-sample *(8 children)* observed for 6 hours at both home and school *(free-living)* | Parent and teacher report- activity level  Direct observation- activity intensity score | **Correlations:**  No significant correlations between parent and teacher report with direct observations. |

**Abbreviations***:* PA= physical activity; SB=sedentary behaviour; LPA= light physical activity; MPA=moderate physical activity; VPA= vigorous physical activity; MVPA=moderate to vigorous physical activity; TPA=total physical activity; MET= metabolic equivalent of task; HR= heart rate; EE=energy expenditure; AEE=activity energy expenditure;MSUP= minimum sitting upright position; CARS= Children’s Activity Rating Scale; OSRAC-P= Observation System for Recording Physical Activity in Children- Preschool; SOFIT-P= System for Observing Fitness Instruction Time for Preschoolers; CPAF= Children’s Physical Activity Form; DO= direct observation; RT3= Triaxial Research Tracker 3; ECG= electrodiagram; HRM = heart rate monitor; AUC-ROC=area under the receiver operating curve; CI= confidence intervals; LoA= limits of agreement; SEP= socioeconomic profile; USA=United States of America; UK=United Kingdom
