# Supplementary material for: A systematic review of the validity, reliability, and feasibility of measurement tools used to assess the physical activity and sedentary behaviour of pre-school aged children
Source: Int J Behav Nutr Phys Act. 2021 Nov 4;18:141. doi: 10.1186/s12966-021-01132-9 (PMC8567581; doi:10.1186/s12966-021-01132-9)
Supplement: Supplementary file 6 — Additional file 6. Study details of level 3 validity evidence. [file 12966_2021_1132_MOESM6_ESM.docx]

**Additional File 6: Study details of level 3 validity studies (n=23)**

| Study details | Methods | | | Units of measure | Validity results |
| --- | --- | --- | --- | --- | --- |
|  | **Measurement tool(s) under study**  *Placement, epoch, cut points, wear time, non-wear time and number of valid days* | **Comparison tool**  *Placement, epoch, cut points* | **Study protocol** *(lab/free living)* |  |  |
| **Direct observation (n=2)** | | | | | |
| **PA and SB (n=2)** | | | | | |
| Sharma et al. (2011) [111];  USA;  n=27; 3-6 years, mean age 3.8 years;  11 male, 16 female  23.1% African American, 65.4% Hispanic, 7.7% Other (White/Native American)  Children recruited from Head Start Centres | **System for Observing Fitness Instruction Time for Preschoolers (SOFIT-P)** | **Accelerometer-**  **Actigraph (GT3X)**  *Right hip*  *5 sec epoch*  *Cut points: Sirard et al., 2005* | Children wore accelerometer for 1 entire school day, no reports on length of observations *(free living)* | SOFIT-P- % time in each PA level (SB, LPA, MPA, MVPA)  Accelerometer- activity counts to determine SB, LPA, MPA, VPA,MVPA | **Pearson’s correlations:**  Correlation between % time in MPA using SOFIT-P with accelerometer data for % time in MPA r=0.506 (p=0.007) and MVPA r=0.532 (p=0.004) and SB r=–0.532 (p=0.004).  Correlation with % time in MVPA on the SOFIT-P with accelerometer data for % time in MVPA r=0.541 (p=0.004), MPA r=0.530 (p=0.004) and SB r=–0.541 (p=0.003). |
| Larson et al. (2011) [110];  USA;  N=4; 3-5 years, mean age not reported;  2 male, 2 female | **Observation System for Recording Physical Activity in Children- Preschool (OSRAC-P)** | **Heart Rate Monitor-**  **Polar F-4**  *Chest and wristwatch*  **Pedometer-**  **New Lifestyles -NL-2000**  *Not reported* | 25 min of structured activity protocol under researchers instruction *(usual activity, but not free living)* | OSRAC-P – Activity levels (1 – stationary or motionless; 2 – stationary with limb or trunk movement; 3 – slow, easy movements; 4 – moderate movements; 5 – fast movements)  Heart rate monitor – beats per minute (bpm)  Pedometer- step count | **Agreement:**  Activity levels 1 and 2 – step total were at or near 0. Most (3/4) participants had highest step total during activity level 5. Step total increased in activity level 3, dropped slightly during activity level 4. Pedometers registered increased steps during activity level 2 even though children were stationary.  Heart rate bpm systematically increased from activity level 1 through to activity level 5. |
| **Accelerometers (n=3)** | | | | | |
| **PA and SB (n=3)** | | | | | |
| Martin et al. (2011) [113];  Scotland, UK;  N=23; 3-5 years, mean age 4.5 years;  9 male, 14 female | **ActivPAL**  *Thigh*  *Wear time:*  *>6 hours per day*  *Non-wear time:*  *Parents to complete daily log to state when devices were removed with reasons. Data between 7am and 9pm eligible for inclusion.*  *Valid n of days:*  *At least 3 days* | **Actigraph (GT1M and GT3X)**  *Right hip*  *15 sec epoch*  *Cut points: <1100 cpm= SB; >1100 cpm = PA*  *Wear time:*  *>6 hours per day*  *Non-wear time:*  *Parents to complete daily log to state when devices were removed with reasons. Data between 7am and 9pm eligible for inclusion.*  *Valid n of days:*  *At least 3 days* | Monitors worn simultaneously for 7 consecutive days during waking hours, expect during water based activities *(free living activity)* | Actigraph- SB and PA  ActivPAL- Sedentary behaviour (time spent sitting/lying plus ‘quiet standing’) and PA (any time not classified as SB) | **Rank order correlations:**  Correlations between the 2 devices r = 0.676 (p <0 .001 and r = 0.663 (p = 0.001), both when Actigraph data were corrected and when uncorrected, respectively.  **Paired t-test:**  Differences in % of time spent sedentary between the monitors were significant when Actigraph data was corrected (p<.001) and not corrected (p=0.04).  **Bland Altman:**  Difference for percentage of time spent sedentary of -4.3% ± 4.8 of daily time (and limits of agreement -14.0–5.4%) when Actigraph data were corrected, and -2.1% ± 4.6 (limits of agreement-11.4–7.2%) when Actigraph data were uncorrected. |
| Byun et al. (2018b) [112];  USA;  n=27; 3-5 years, mean age 4.9 years;  16 male, 11 female | **Fitbit (Flex)**  *Non dominant wrist*  *Non-wear time:*  *≥90 minutes consecutive zero counts*  *Parents asked to report times for non-wear period, unusual PA and sleep.* | **Actigraph (GT3X+)**  *Right hip*  *15 sec epoch*  *Cut points: Evenson et al., 2008; Pate et al., 2006*  *Non-wear time:*  *≥90 minutes consecutive zero counts*  *Parents asked to report times for non-wear period, unusual PA and sleep.* | Children wore both monitors simultaneously for 24 hours *(free living)* | Fitbit- SB, LPA, MPA, VPA  Actigraph- Activity counts to determine SB, MVPA, TPA | **Pearson’s correlation:**  Correlations between Actigraph and Fitbit were all significant. SB r=0.85 [AG_E_], r=0.87 [AG_P_] (p<0.01), TPA r=0.69 [AG_E_], r=0.71 [AG_P_] (p<0.01), MVPA r=0.59 [AG_P_], r=0.58 [AG_E_] (p<0.01).  Overall correlations stronger between the Fitbit and Actigraph using Pate cut points r=0.71-0.87 (p<0.01), than Evenson cut points r=0.69-0.85 (p<0.01)  **Mean absolute percent errors (MAPE):**  No difference in the estimated  SB when the FF was compared with the AG, although the FF recorded slightly higher minutes of SB than the AG_P_ and the AG_E_ (FF, 673 minutes; AG_P_, 631 minutes; AG_E_, 602 minutes). The MAPE was lower when the FF was compared with the AG_P_ (9.1%) than with the AG_E_ (13%).  The FF produced significantly lower MVPA estimates than the AG_P_ and the AG_E_ (FF, 32 minutes; AG_P_, 99 minutes; and AG_E_, 62 minutes); the MAPE was greater for the comparison with the AG_P_ (70.1%) than with the AG_E_ (55.7%).    No significant difference in total PA and MAPE was found between the FF and AG_P_ (FF, 337 minutes vs AG_P_, 379 minutes; MAPE, 14.3%), but the difference and MAPE was slightly greater between the FF and the AG_E_ (AG_E_, 407 minutes; MAPE, 18.1%). |
| Shin (2015) [114];  USA;  N=19; 3-5 years, mean age 4.6 years;  10 male, 9 female | **Best Fit Friend (BFF)**  **accelerometer**  *Left and right wrist, back, waist, chest, right ankle, upper arm*  *Cut points: determined using ROC-AUC analysis* | **Actigraph (GT3X and GT3X+)**  *Waist and right wrist*  *1 sec epoch* | Children followed structured activity protocol at the pre-school under researchers instruction *(usual activity, not free living)* | BFF Accelerometer- Raw signal data  Actigraph Accelerometer- Vector magnitude counts | **Pearson’s Correlation:**  No significant correlations between BFF and Actigraph at either the wrist or waist, for all activities. |
| **Pedometers (n=6)** | | | | | |
| **PA and SB (n=2)** | | | | | |
| Pagels et al. (2011) [116];  Sweden/USA;  N=55; 3.4-5.7 years, mean age 4.5 years; 28 male, 27 female | **Pedometer –**  **Yamax Digi-Walker (SW-200)**  *Right side of waist* | **Accelerometer-**  **Actigraph (GT1M)**  *Waist belt*  *15 sec epoch*  *Cut points: Sirard et al., 2005* | Children wore monitors simultaneously for 5 days during nursery time *(free living)* | Pedometer- step counts  Accelerometer- activity counts | **Linear regression analyses:**  Correlation between: 1) Mean step counts and mean accelerometer counts r=0.67 (p<0.001), accounting for 45% of variance and minutes of light to vigorous PA r=0.76 (p=0.001).  Correlation between step counts and MVPA in 3 year olds r=-0.19 (p=0.191) and 4-5 year olds combined r=0.50 (p=0.001). |
| Saris & Binkhorst (1977) [137];  Netherlands;  N=4; 4-6 year, mean age not reported; sex not reported | **Pedometer (type not reported)**  *Right side of waist* | **Actometer**  *Right ankle and right wrist* | Children engaged in free play activity in their pre-school setting *(free living)* | Pedometer and Actometer units | **Correlations:**  Correlations between pedometer and actometer ankle r=0.89 (p<0.001); actometer wrist r=0.78 (p<0.05); actometer total r=0.95 (p<0.001).  Correlations between actometer total and 1) actometer ankle r=0.96 (p<0.001) and 2) actometer wrist r=0.86 (p<0.05). No significant correlation between actometer ankle and wrist r=0.69. |
| **PA (n=4)** | | | | | |
| De Craemer et al. (2015) [118];  Belgium;  N=41; 4-6 years, mean age 5.4 years;  21 male, 20 female | **Pedometer** *-*  **Omron Walking Style Pro (HJ-720IT-E2)**  *Right hip*  *Wear time:*  *≥5 hours data (recorded between 7am and 8pm weekdays, 7am and 9pm weekend days)*  *Non wear time:*  *Manually checked, if both monitors registered 0 per hour, the hour was deleted.* | **Actigraph (GT1M)**  *Left hip*  *15 sec epoch*  *Wear time:*  *≥5 hours data (recorded between 7am and 8pm weekdays, 7am and 9pm weekend days)*  *Non wear time:*  *Manually checked, if both monitors registered 0 per hour, the hour was deleted.*  *Valid n of days:*  *At least 1 day of data.* | Children wore monitors simultaneously for four days *(free living)* | Pedometer- step count  Accelerometer- step count and activity count | **Pearson’s correlations:**  Pedometer based steps correlated with accelerometer activity counts per hour r=0.65 (p<0.001) and per day r=0.64 (p<0.001), and with accelerometer step count per hour r=0.92 (p<0.001) and per day r=0.89 (p<0.001).  **Independent samples t-test**  No significant difference on group level between hourly steps (p=0.359) and daily steps (p=0.592) from both devices during the four days.  **Paired sample t-tests:**  Significant difference on the individual level between hourly steps from both devices (p=0.001), but no significant difference was found between daily steps (p=0.122)  **Bland Altman:**  Agreement between step count on the devices- Bias of 221.81 (±1679.78), limits of agreement ranged from -3070.57 to 3514.18 steps per day (accelerometer-pedometer). |
| Lee et al. (2014) [119];  Korea;  N=131; 3-6 years, mean age not reported; sex not reported | **Pedometer-**  **Omron HJ-720ITC**  *Not reported* | **Accelerometer-**  **Actigraph (GT3X)**  *Not reported*  *No cut points reported* | 7 days activity *(free living)* | Accelerometer-MVPA  Pedometer- step count | **Pearson’s Correlation:**  Correlations between total steps and MVPA were r=0.59 for 7 days, r=0.55 for weekdays and r=0.37 for weekend days.  Younger age groups (3–4 years) showed higher correlation coefficients than older age groups (5–6 years): 7 days, r=0.67 vs. 0.54; weekday, r=0.60 vs. 0.52; and weekend, r=0.56 vs. 0.38, respectively. |
| Bikchu (2014) [117];  Hong Kong;  N=143; 4-5 years, mean age not reported;  80 male, 63 female | **Pedometer-**  **Yamax Digi-Walker (SW-700)**  *Left side of waist* | **Accelerometer-**  **RT3 (StayHealthy)**  *Right side of waist*  *60 sec epoch* | 30 minutes of structured activity protocol whilst wearing monitors simultaneously *(usual activity, but not free living)* | Accelerometer- activity units presented as vector magnitude score  Pedometer- step count | **Pearson’s correlations:**  Correlations for various activities between devices ranged from r=0.24 to r=0.78, with sliding (r=0.16 for girls) and cycling (r=0.26 for girls) having the lowest correlations. While correlations were all higher than r=0.57 for the other 8 play activities (crawl, throw/catch, kick, roll, agility run, walk balance, rhythmic, hopscotch). |
| Cardon & De Bourheaudhiuj (2007) [115];  Belgium;  N=76; 4-5.9 years, mean age 4.9 years; 37 male, 39 female  55% from high SEP;  45% from low SEP | **Pedometer-**  **Yamax Digiwalker (SW-200)**  *Left hip*  *Wear time:*  *Pedometer data satisfactory when missing data (reported by parent) did not exceed 1 hour on 1 or more days.*  *First day of data omitted (resulting in data being collected on 2 weekdays and 2 weekend days).*  *Diary for parents to record daily step count, as well as time frame and reasons for removing the devices.* | **Accelerometer-**  **Actigraph (MTI AM 7164)**  *Right hip*  *15 sec epoch*  *Cut points: Sirard et al.,2005*  *Wear time:*  *>8.6 hours per day*  *First day of data omitted (resulting in data being collected on 2 weekdays and 2 weekend days).*  *Diary for parents to record daily step count, as well as time frame and reasons for removing the devices.* | Monitors worn during waking hours for 5 consecutive days *(free living)* | Pedometer- step counts  Accelerometer- Vector magnitude accelerometer output to determine minutes engaged in MVPA | **Pearson’s correlation:**  Correlation between mean step counts and minutes of MVPA engagement r=0.73 (p<0.001). |
| **Proxy reported measurement tools (n=12)** | | | | | |
| **PA and SB (n=7)** | | | | | |
| Bacardi-Gascón et al. (2012) [124];  Mexico;  N=35; 3-5 years, mean age 4.4 years;  17 male, 18 female | **Questionnaire developed for parents of pre-schoolers in Mexico**  Broad question on PA level | **Accelerometer- Actigraph (GT1M)**  *Right hip*  *15 sec epoch*  *Cut points: Pate et al.,2006 and Sirard et al., 2005*  *Wear time:*  *10 hours per day*  *Valid n of days:*  *3 weekdays and 1 weekend day* | No specific time frame- questionnaire assesses usual activity, accelerometer worn for 4 days *(free living)* | Questionnaire: Minutes and % of time in different intensities of PA (low-reported as SB, moderate, vigorous)  PA level category: inactive to very active  Actigraph accelerometer: % of time spent in different intensity of activities | **Spearman’s correlation***:*  Significant correlation between parents’ answers to broad PA question and accelerometer (Sirard cut points) for time in MPA, VPA r=0.57 and MVPA r=0.62 (both p<0.001).  Correlations between % SB time reported by questionnaire and accelerometer; Sirard cut point: r=0.35 (p<0.05) and Pate cut points: r=0.34 (p<0.05).  Correlations for % time spent in VPA and MVPA as reported by questionnaire were significant correlated with VPA as assessed by accelerometry; Sirard cut point: r=0.53 (p=0.001) and Pate cut point: r=0.41 (p=0.01) |
| Dwyer et al. (2011) [122];  Australia;  N= 67; 3-5 years, mean age 3.8 years;  35 male, 32 female  91% White, 3% Mediterranean, 6% other ethnicity.  9% low SEP, 34% middle,  57% high | **Pre School Physical Activity Questionnaire (PRE-PAQ)** | **Accelerometer- Actigraph (MTI 7164)**  *Right hip*  *15 sec epoch*  *Cut points: Sirard et al., 2005 and Reilly et al., 2003*  *Wear time:*  *>6 hours of recorded activity per day (between 8am and 6pm)*  *Non wear time:*  *>20 minutes of 0 counts not reported by parent log explaining when device had been removed*  *Valid n of days:*  *First day data excluded and accelerometer had to have 3 days of monitoring.* | 3 day activity *(free living)* | Pre-PAQ:  1-Stationary (no movement); 2- Stationary (limb/trunk movement); 3- moving slowly; 4- moving at a medium or moderate pace; 5- moving at a fast pace.  Accelerometer:  Time in different intensities: Stationary, SB, LPA, MPA, VPA, MVPA, light MVPA (or non sedentary) | **Bland Altman:**  Agreement between PRE-PAQ and accelerometer (mean difference in mins per day):  Stationary: 7.6 (LoA -141.3 to 156.4)  SB: -208.6 (LoA -349.8 to -67.5 using Reilly cut points); -235.4 (LoA -383.1 to -87.7 using Sirard cut points)  LPA: -4.8 (LoA -105.5 to 96.0 mins per day)  MPA: 48.2 (LoA -24.9 to 121.3 mins per day)  VPA: 1.9 ( LoA -37.5 to 41.3 mins per day)  MVPA: 50.1 (LoA -42.9 to 143.1 mins per day)  Non-Sedentary: 20.9 (LoA -121.9 to 163.7 mins per day)  LMVPA: 45.2 (LoA -103.6 to 194.1 mins per day)  Wide limits of agreement.  **Pearson’s Correlations:**  Stationary: r=0.25  Sedentary: r=0.28 (Reilly cut points), r=0.19 (Sirard cut points)  LPA: r=-0.07  MPA: r=0.13  VPA: r=0.17  MVPA: r=0.17  Non-sedentary: r=0.16  LMVPA: r=0.05 |
| Janz et al. (2005) [123];  USA;  N=204; 4-7 years, mean age 5.7 years;  91 male, 113 female  96% Caucasian  Almost all had families of relatively high SEP; only 10% listed a family income below 20,000 dollars. | **Netherland’s Physical Activity Questionnaire (NPAQ)** | **Accelerometer- Actigraph (MTI 7164)**  *Waist*  *Cut point for vigorous activity: ≥2818*  *Wear time:*  *At least 8 hours per day*  *Valid n of days:*  *At least 3 days of data.* | Questionnaire asks about activity over past 6 months, remaining protocol 4 days including 1 weekend day *(free living)* | NPAQ: Total activity score  Actigraph: activity counts to determine TPA and VPA | **Spearman’s correlation***:*  NPAQ and accelerometer derived variables were r=0.33 (p<0.01) for total activity and r=0.36 (p<0.01) for vigorous activity.  TV viewing and accelerometry-derived variables were r= -0.16 (p <0.05) for total activity and r= -0.18 for vigorous activity (p <0.05).  Associations between individual NPAQ items and accelerometer variables were lower than associations between total NPAQ score and accelerometry.  **Logistic Regressions:**  Children with high NPAQ scores were 2.7 times more likely to be in the upper tertile for total activity than those with low or moderate NPAQ scores, and 3.2 times more likely for vigorous activity.  Children with high TV viewing score were 10% less likely to be in the upper tertile of total activity when compared to children with low or moderate TV viewing scores. |
| Manios et al. (1998) [121];  Greece;  N=39; 6 years,  17 male, 22 female | **Proxy report measures (Teacher Activity Rating / 3 Day Leisure Time Report –parental reported)** | **Heart rate monitoring –Sport Tester 3000**  *Chest and wrist watch* | ‘Real time’ activity- 5 day reports for teacher, 3 days for leisure reports, 3 days HRM *(free living)* | Teacher rating: time spent in MVPA  Parent rating: Time spent in different intensities of PA (SB, LPA, MVPA- focus on MVPA)  Heart rate monitor: Activity intervals to determine MVPA (based on >149 bpm or above for at least 10 minutes) | **Spearman’s correlation:**  Teacher Report- Significant correlations between reports and activity intervals during school hours r=0.587 (p<0.001), and during the 3 days of HRM recording r=0.406 (p<0.05), not significant for weekend days r=0.072 (p>0.05).  Leisure Time Report- Significant correlation between report and activity intervals for the 3 day report r=0.715 to 0.815 (p<0.001), significant correlations between MVPA reported on leisure report and activity intervals during the same days r=0.68 (p<0.001). |
| Nishikido et al. (1982) [98];  Japan;  n=49; 5-6 years, mean age not reported;  25 male, 24 female | **Proxy report of children’s Habitual Physical activity (Questionnaires to mothers and teachers)** | **Pedometer-**  **Yamasa AM-5**  *Hip* | Questionnaire assesses habitual activity. Remaining protocol for 2 days of children engaging in usual activities in pre-school setting *(free living)* | Proxy report: Children rated as: Inactive; relatively inactive; medial; relatively active; active.  Yamasa AM-5: Step count | **Kendall’s rank order correlation:**  Teacher’s evaluations significant correlations with pedometer step rate r=0.25 (p<0.05). No other significant correlations. |
| Wen et al. (2010) [125];  Australia;  n=31 ; 3-5 years, mean age 3.5 years;  19 male, 12 female  Children recruited from centers said to vary in SEP. | **Brief Survey of Activity Preferences (based on NPAQ)**  **7 day activity diary (adapted from CLASS)** | **Accelerometer- Actigraph (GT1M)**  *Right hip*  *15 sec epoch*  *Cut points: Sirard et al., 2005*  *Wear time:*  *At least 8hr wear time per day*  *Non wear time:*  *Consecutive 0 counts for at least 60 minutes*  *Valid n of days:*  *At least 4 days of data* | 7 day activity measured by diary and accelerometer; brief survey of habitual activity *(free living)* | Brief survey: activity preferences  Diary: Total time in PA and SB (PA defined as walking and other MVPA activities.)  Actigraph: activity counts to determine SB, MPA, VPA, and steps count | **Spearman’s correlation:**  Brief survey was not significantly correlated with any of the accelerometer outcomes (correlations ranged from -0.11 to 0.24).  Significant correlations between the diary outcomes for PA (excluding walking) r=0.42 (p=0.02) and total PA r=0.44 (p=0.014) in comparison with vigorous PA determined by accelerometer. No other significant correlations, including no significant correlations for screen time or sedentary time. |
| Ettienne et al. (2016) [86];  USA;  n=45; Mean age =3.5 years; 27 male, 18 female  Children were primarily native Hawaiian or other Pacific Islander- all of mixed ethnicities.  Children recruited from Head Start sites | **Parental reported activity logs** (report all children’s activities for 3 days) | **Actical (Respironics, Philips)**  *Non dominant wrist*  *15 sec epoch*  *Cut points: Schaefer et al., 2014*  *Non-wear time: >1300 minutes of SB per day.* | 7 days habitual activity examined by accelerometer and log *(free living)* | Accelerometer- activity counts to determine activity intensity- SB, LPA, MPA, VPA.  Parental logs- Metabolic equivalent of task (MET) values and activity categories of behaviours | **Cohen’s Kappa:**  Proportion of agreement between the accelerometers and PA logs was 40%, weighed kappa coefficient was 0.15 (p<0.001).  PA logs compared to accelerometer underestimated moderate and vigorous types of activity. |
| **PA (n=4)** | | | | | |
| Chen et al. (2002) [120];  Japan;  n=21; 3-4 years, mean age 3.8 years;  12 male, 9 female | **Nursery teacher’s proxy report (based on Toyama Cohort Study survey questions)** | **Accelerometer- Actiwatch-L**  *Ankle*  *1 min epoch*  Caloriecounter- *Waist*  *4 sec epoch* | Nursery teachers report of activity during the past week, remaining protocol for 3 days *(free living)* | Proxy report: frequency, level and preferences of activity  Accelerometer Actiwatch-L: Activity counts  Caloriecounter: TEE; energy expenditure from PA (EEPA); step count. | **ANOVA:**  Children rated ‘very often’ activity had a significantly higher level of TEE, mean activity counts and daytime activity counts per day, compared with peers rated ‘not often’ (1257.1 ±101.9 kcal vs. 1148.1 ±71.4 kcal, p=0.019; 570.5 ±192.8 counts vs. 334.9 ±123.4 counts, p=0.026; 796.1 ±194.9 vs. 486.7 ±191.7 counts, p=0.014, respectively).  Children rated ‘very active’ had a significantly higher TEE, EEPA, steps, mean activity counts and daytime activity counts, compared with ‘inactive’ peers (1257.1 ±101.9 kcal vs. 1126.7 ±124.4 kcal, p=0.024; 140.6 ±17.5 kcal vs. 78.2 ±14.7 kcal, p=0.03; 16103 ±1896 steps vs. 10038 ±320 steps, p=0.031; 570.5 ±192.8 counts vs. 215 ±165.8, p=0.002; 796.1 ±194.9 vs. 396.6 ±336.2 counts, p=0.02, respectively)  Children with PA rated as "like very much" had higher activity counts than those with PA rated as "like"(p<0.05). No other significant correlations between likeability of PA and indices of PA. |
| Chow & Au (2009) [126];  Hong Kong;  n=23; 5-6 years, mean age not reported;  12 male, 11 female  Majority of the children from lower to lower-middle families. | **Habitual Activity Estimation Scale (HAES)– Parent Proxy report** | **Pedometer-**  **Yamax SW-700**  *Right side of waist*  *Parents asked to report time of child waking up in the morning, sleeping at night and taking naps during the pedometer data collection period. Step count reading shown by the pedometer recorded by parent every day of use.* | Questionnaire examining usual activity, protocol involved 4 days of habitual activity *(free living)* | HAES- % MVPA  Pedometer- daily step count (recorded each night by parent) | **Pearson’s correlation:**  No significant correlation between parent report of %MVPA and daily step counts of children for weekday r=-0.33 (p=0.13) or weekend r=0.17 (p=0.44). |
| Corder et al. (2009) [68];  UK;  N=27; 4-5 years, mean age 4.9 years;  17 male, 10 female | **Children’s physical activity questionnaire (CPAQ)** | **Accelerometer- Actigraph (MTI 7164)**  *Hip*  60 sec epoch  *Cut points: >3000 cpm*  *Wear time:*  *>600 minutes of valid data per day*  *Non wear time:*  *20 minutes of consecutive 0* | Questionnaire for past 7 days, remaining protocol for 11 days assessing habitual activity *(free living)* | CPAQ- Physical activity energy expenditure (PAEE) and time spent in MVPA  Actigraph- Time spent in MVPA | **Spearman’s correlations:**  MVPA assessed by CPAQ significantly correlated with MVPA obtained from accelerometer r=0.42 (p=0.04).  **Bland Altman:**  Mean bias (MVPA) = 235.9 ±362.0 (95% CI 86.5, 385.3). |
| Telford et al. (2004) [127];  Australia;  n=58; 5-6 years, mean age 5.3 years;  37 male, 21 female | **Children’s Leisure Activities Study Survey (CLASS)** | **Accelerometer- Actigraph (MTI AM7164)**  *Waist*  *1 min epoch*  *Cut points: Freedson et al., 1997*  *Wear time:*  *10,000 movement counts per day*  *Non wear time:*  *When 10,000 movement counts per day were not collected it was assumed that the device had not been worn*  *Valid n of days:*  *At least 4 days of data* | Usual activity, accelerometer 8 days during waking hours *(free living)* | CLASS- units of time spent in MPA, VPA and MVPA (total) PA.  Actigraph- activity counts to determine time (minutes per week) spent in moderate and vigorous intensity activites | **Spearman’s correlation:**  No significant correlation between proxy and accelerometer for MPA r=-0.06, VPA r=-0.04, TPA r=-0.04 or raw movement counts per day r=0.05 (individual level).  Mean difference between proxy reported and accelerometer for MPVA was -135.8 min per day (95% CI -155.3 to -116.2), VPA -5.0 min per day (95% CI -13.3 to 3.4), TPA -140.7 min per day (95% CI -164.9 to -116.6). |
| **SB (n=1)** | | | | | |
| Mendoza et al. (2013) [128];  USA;  N=96; 3-5 years, mean age 4.7 years;  53 male, 41 female (sex of 2 participants not reported)  100% Latino or Hispanic.  Children recruited from Head Start Centers | **TV Diary** | **Accelerometer-**  **Actigraph (GT1M)**  *Hip*  *15 sec epoch*  *Cut points: Reilly et al., 2003*  *Wear time:*  *≥ 8 hours per day*  *Valid n of days:*  *≥1 day*  TV allowance  Ecological Momentary Assessment (EMA) | Measurement tools administered simultaneously for 7 days *(free living)* | Diary, TV allowance and EMA- TV and video viewing (mins per day)  Accelerometer - Activity counts to determine SB | **Spearman’s Correlation:**  TV diary was not significantly correlated with accelerometer, but was significantly correlated with TV allowance (time 1 r=0.45, p<0.001 and time 2 r=0.55, p<0.001) and EMA (time 1 r=0.47, p<0.001 and time 2 r=0.51, p<0.001). |

**Abbreviations***:* PA= physical activity; SB=sedentary behaviour; LPA= light physical activity; MPA=moderate physical activity; VPA= vigorous physical activity; MVPA=moderate to vigorous physical activity; LMVPA= light moderate to vigorous physical activity; TPA=total physical activity; MET= metabolic equivalent of task; TEE =total energy expenditure; EEPA= energy expenditure from physical activity; bpm= beats per minute; cpm= counts per minute; OSRAC-P= Observation System for Recording Physical Activity in Children- Preschool; SOFIT-P= System for Observing Fitness Instruction Time for Preschoolers; AG= Actigraph; FF =Fitbit Flex; BFF= Best fit friend; MAPE= mean absolute percent erros; RT3= Triaxial Research Tracker 3; HRM = heart rate monitor; PRE-PAQ= Pre School Physical Activity Questionnaire; NPAQ= Netherland’s Physical Activity Questionnaire; HAES= Habitual Activity Estimation Scale; CPAQ= Children’s physical activity questionnaire; CLASS = Children’s Leisure Activities Study Survey; EMA= Ecological Momentary Assessment; ROC-AUC=area under the receiver operating curve; CI= confidence intervals; LoA= limits of agreement; VM=vector magnitude; SEP= socioeconomic profile; USA=United States of America; UK=United Kingdom
