# Supplementary material for: A systematic review of the validity, reliability, and feasibility of measurement tools used to assess the physical activity and sedentary behaviour of pre-school aged children
Source: Int J Behav Nutr Phys Act. 2021 Nov 4;18:141. doi: 10.1186/s12966-021-01132-9 (PMC8567581; doi:10.1186/s12966-021-01132-9)
Supplement: Supplementary file 7 — Additional file 7. Study details of level 4 validity evidence. [file 12966_2021_1132_MOESM7_ESM.docx]

**Additional File 7: Study details of level 4 validity evidence (n=9)**

| Study details | Methods | | Units of measure | Validity results |
| --- | --- | --- | --- | --- |
|  | **Measurement tools under study**  *Placement, epoch, cut points, wear time, non-wear time and number of valid days* | **Study protocol** *(lab/free living)* |  |  |
| **Combined heart rate and accelerometry and accelerometers (n=1)** | | | | |
| **PA and SB (n=1)** | | | | |
| Adolph et al. (2012) [57];  USA;  n=64; 3-5 years, mean age 4.5 years;  37 male, 27 female  50% White, 27% Black, 22% Hispanic, 2% Asian | **Actiheart (MiniMitter)**  *Chest*  *15 sec epoch*  **Actical (Respironics, MiniMitter)**  *Right hip*  *15 sec epoch*  **Triaxial Research Tracker 3 (RT3- Stayhealthy)**  *Right hip*  *1 sec epoch*  *Cut points: Piecewise linear regressions determined thresholds into sedentary, light, moderate and vigorous levels of PA* | All accelerometers worn during 3 hour whole room calorimeter protocol *(laboratory based)* | Accelerometers- activity counts per minute | **Pearson’s correlations:**  Accelerometer counts from each of the devices were significantly correlated with one another r=0.80-0.95 (p=0.001). |
| **Accelerometers (n=6)** | | | | |
| **PA and SB (n=3)** | | | | |
| Alhassan et al. (2017) [76];  USA;  N=33; mean age 4.4 years;  21 male, 12 female | **Actiwatch (Spectrum)**  *Non dominant wrist*  *15 sec epoch*  *Cut points: Ekblom et al., 2012*  **Actigraph (GT3X)**  *Waist, centre of lower back*  *15 sec epoch*  *Cut points: Pate et al., 2006 and Sirard et al., 2006* | Monitors worn 24 hours per day for up to 16 days *(free living)* | Accelerometers- activity counts to determine SB, LPA, MPA, VPA, MVPA | **Spearman’s Correlation:**  Correlation between Actiwatch and Actigraph r=0.41. |
| Kelly et al. (2004) [83];  UK;  n=78; 3-4 year olds, mean age 3.5 years; 30 male, 48 female | **Actiwatch (AW16)**  *Right hip*  *1 min epoch*  **Actigraph (CSA/MTI – WAM 7164)**  *Right hip*  *1 min epoch* | 39-45 minutes structured play class *(usual activity, reflective of free living)* | Accelerometers- activity counts per minute | **Correlation:**  Correlation between Actigraph and Actiwatch counts r = 0.36 (p< 0.01). |
| Vanderloo et al. (2016) [129];  Canada;  N=23; 4-5 years, mean age 5.08 years;  12 male, 16 female (sex sample size prior to exclusion of participants) | **Actical (B series)**  *Right hip*  *15, 60 sec epoch*  *Cut points: Pfeiffer et al., 2006*  **Actigraph (GT3X+)**  *Right hip*  *15, 60 sec epoch*  *Cut points:* *Pate et al., 2006*  **Both devices-**  *Wear time:*  *8 hours per day*  *Non-wear time:*  *>60 minutes of consecutive zeroes*  *Valid n of days:*  *>3 days valid data*  *All wear time information recorded in daily log by parents/guardians.* | Both monitors worn simultaneously for 7 consecutive days *(free living)* | Accelerometers- Activity counts to determine SB, TPA, MVPA | **Paired t-tests, with Bonferroni correction:**  Lower rates of both MVPA and TPA were measured with the Actical method compared with Actigraph (p<0.001). Higher SB by Actical compared with Actigraph (p<0.001), at both 15 and 60s epoch.  At 15s epoch: significantly lower rates of MVPA (t[22] = –12.75, p <0.00, Cohen’s d = –2.93) and TPA (t[22] = –5.75, p <0 .00, Cohen’s d = –1.52) measured with the Actical compared with the ActiGraph. Significantly higher level of SB via the Actical in comparison with the ActiGraph (t[22] = 11.00, p <0.00, Cohen’s d = 1.73).  At 60s epoch: significantly lower rates of MVPA (t[22] = –11.57, p <0.00, Cohen’s d = –2.87) and TPA (t[22] = –12.50, p <0.00, Cohen’s d =–2.54) measured via the Actical in comparison with the ActiGraph. Significantly higher level of SB with the Actical in comparison with the ActiGraph (t[22] = 12.41, p < .00, Cohen’s d = 2.14).  **Bland Altman (mean difference in minutes):**  15s epoch: SB (9.18±7.84), MVPA (-6.61±4.87), TPA (-9.41±15.37).  60s epoch : SB (12.70±9.62), MVPA (-6.78±5.50) and TPA (-14.19±10.67).  The difference between Actical and Actigraph: 95.7% (MVPA), 95.7% (TPA) and 95.7-100% (SB) of the values were within 2SD of the difference between the devices. |
| **PA (n=1)** | | | | |
| Hislop et al. (2012b) [80];  Scotland, UK;  N=31; 3-5 years, mean age 4.4 years;  15 male, 16 female | **Actigraph (GT1M)**  *Waist band*  *1 sec epoch*  *Cut points:* *Sirard et al., 2005, Freedson et al., 2005, Pate et al., 2006, Evenson et al.., 2008, Van Cauwenberghe et al., 2011, Puyau et al., 2002*  **RT3 (Stayhealthy)**  *Waist band*  *1 sec epoch*  *Cut points: Vanhelst et al., 2000, Rowlands et al.,2004, Sun et al., 2008, Chu et al.,2007* | 60 min free play in nursery setting *(free living)* | Accelerometers- Activity counts | **Pearson’s Correlation:**  Significant correlation in total counts between the two accelerometers r=0.72 (p <.001). |
| **SB (n=2)** | | | | |
| De Decker et al. (2013) [78];  Belgium;  n=52; mean age 5.5 years;  26 male, 26 female | **ActivPAL**  *Thigh*  *15 sec epoch*  **Actigraph (GT1M)**  *Right hip*  *15 sec epoch*  *Cut points: Evenson et al., 2008*  **Both devices-**  *Wear time: First day of data deleted, minimum of 6 hrs per day required ( this included data between 7am and 9pm on weekdays, 8am and 8pm on weekend days)*  *Non wear time:*  *≥10 min of consecutive zero activity counts.*  *Valid n of days: 3 days required to be included* | Children wore monitors simultaneously for 5 days *(free living)* | ActivPAL: Posture allocation- Time spent in sit/ lie, stand or stepping.  Actigraph: Activity counts to determine sedentary or non-sedentary behaviour | **ANOVA:**  Significant differences between the Actigraph and ActivPAL data (p<0.05).  **Bland Altman:**  Mean time defined as SB was lower for ActivPAL compared to Actigraph (mean bias -7.71%±10.87%).  No systematic bias for SB and wide limits of agreement in 95% confidence (-29.01% to 13.6%). Mean time defined as SB including standing still indicated a mean bias of -7.26%±21.15% and wide limits of agreement (-48.75%to 34.21%) were found in 95% confidence analysis. |
| Van Cauwenberghe et al. (2012) [130];  New Zealand;  N=49; 3-4 years, mean age 4 years;  22 male, 27 female | **Actical (Respironics, MiniMitter)**  *Hip (above the right iliac crest)*  *15 sec epoch*  *Cut points: Evenson et al., 2008*  **ActivPAL**  *Thigh*  *15 sec epoch*  *Wear time:*  *At least 3 hours of data from both devices* | Monitors worn simultaneously during 1 day at nursery *(free living)* | Both monitors: time spent in SB and non-SB | **Bland Altman:**  Group mean time in SB as detected by the Actical and activPAL was similar and that the 95% limits of agreement for time in SB were wide. No obvious systematic bias.  **K statistic:**  The κ statistic = 0.46 (95% CI: 0.45–0.47; p < 0.001).  **Percent agreement:**  The difference in SB between both monitors for each participant showed 73% agreement and 27% disagreement  ActivPAL classified 15.4% of all epochs as standing still, with 54.9% of these defined as SB by Actical.  Epochs defined as SB by Actical and non-SB by ActivPAL, 61.6% regarded as standing still by ActivPAL. |
| **Proxy reported measurement tools (n=2)** | | | | |
| **PA and SB (n=1)** | | | | |
| Nishikido et al. (1982) [98];  Japan;  n=49; 5-6 years, mean age not reported;  25 male, 24 female | **Proxy report of children’s habitual physical activity (Mothers and teachers)** | Questionnaire assesses habitual activity *(free living activity)* | Proxy report- Children rated as: Inactive; relatively inactive; medial; relatively active; active. | **Kendall’s rank order correlation:**  Correlation between mother and teacher evaluations were r= 0.41 (p<0.01) and r=0.55 (p<0.001) in school A and B, respectively. |
| **PA (n=1)** | | | | |
| Noland et al. (1990) [100];  USA; n=21; 3.8-5.6 years, , mean age 4.7 years;  11 male, 10 female  90% White, 10% Black  Families were primarily middle and upper class. | **Parent, Teacher and Child Report** | Habitual overall activity level *(free-living)* | Parent and teacher report- activity level  Child report- activity preference | **Correlations:**  Significant correlation between behaviourally anchored rating from parents and teachers r=0.40 (p<0.05).  Children’s activity preference not significantly correlated with parents or teachers behaviourally anchored rating. |

**Abbreviations**: PA= physical activity; SB= sedentary behaviour; LPA= light physical activity; MPA=moderate physical activity; VPA=vigorous physical activity; MVPA= moderate to vigorous physical activity; TPA= total physical activity; RT3= Triaxial Research Tracker 3
