# Supplementary material for: A systematic review of the validity, reliability, and feasibility of measurement tools used to assess the physical activity and sedentary behaviour of pre-school aged children
Source: Int J Behav Nutr Phys Act. 2021 Nov 4;18:141. doi: 10.1186/s12966-021-01132-9 (PMC8567581; doi:10.1186/s12966-021-01132-9)
Supplement: Supplementary file 8 — Additional file 8. Study details of reliability evidence. [file 12966_2021_1132_MOESM8_ESM.docx]

**Additional file 8: Study details of studies examining reliability of measurement tools (n=11)**

**Test-retest reliability of measurement tools (n=10)**

| **Study details** | **Methods** | | **Units of Measure** | **Reliability Results** |
| --- | --- | --- | --- | --- |
|  | **Measurement tool** | **Test Retest period** |  |  |
| **Accelerometers and pedometers (n=2)** | | | | |
| **PA (n=2)** | | | | |
| Bikchu (2014) [117];  Hong Kong;  N=143; 4-5 years, mean age not reported.  80 male, 63 female | Pedometer- Yamax Digi-Walker SW-700  *Left side of waist* | Same structured activity protocol whilst wearing monitors simultaneously completed at 2 time points | Step count | **Intaclass correlation coefficient:**  ICC= 0.34 to 0.87. |
|  | Accelerometer- TriTrac RT3 triaxial (StayHealthy)  *Right side of waist*  *60 sec epoch* |  | Vector magnitude score | ICC= 0.38 to 0.82. |
| Murray (2009) [99];  USA;  n=75; 3-5 years, mean age 4.4 years;  31 male, 44 female  34.3% African American, 64.4% Hispanic, 1.4% Native American  Children recruited from Head Start Centres | MVP 4 Walk4Life Digital Pedometer  *Left or right hip* | Each child was observed by two measurements at baseline and two measurements at post-test whilst wearing pedometer and engaging in free play activities. | Step counts  MVPA  Activity time | **Pearson’s Correlation:**  No significance at pre-test, yet show significant results for all PA outcomes at post-test:  Step counts r=0.52 (p<0.001).  MVPA r=0.57 (p<0.001)  Activity time r=-0.55 (p<0.001). |
| **Proxy reported tools (n=8)** | | | | |
| **PA and SB (n=5)** | | | | |
| Bacardi-Gascón et al. (2012) [124];  Mexico;  N=21; 3-5 years, mean age not reported;  sex not reported | Questionnaire developed for parents of pre-schoolers in Mexico | 7 days | Duration of activities in:  Low  Moderate  Vigorous | **Correlations:**  Low r=0.86 (p=0.01)  Moderate r= 0.79 (p=0.04)  Vigorous r=0.94 (p=0.002) |
|  | Broad question on PA level |  |  | r=0.97 (p=0.001) |
| Dwyer et al. (2011) [122];  Australia;  N=103; 3-5 years, mean age 3.8 years;  54 males, 49 females  87% Caucasian, 6% Mediterranean, 7% other ethnicity  7% low SEP, 29% middle, 64% high | Pre School Physical Activity Questionnaire (PRE-PAQ) | 1-2 weeks | Pre-PAQ levels 1-2  Pre-PAQ levels  Pre-PAQ levels 4  Pre-PAQ levels 5 | **Intraclass correlation coefficient**:  ICC= 0.44  ICC =0.53  ICC =0.44  ICC =0.64 |
| Janz et al. (2005) [123];  USA;  n=72; 4-7 years, mean age 5.7 years;  sex not reported  96% Caucasian  Almost all had families of relatively high socioeconomic status; only 10% listed a family income below 20,000 dollars. | Netherland’s Physical Activity Questionnaire (NPAQ) | 2-8 weeks | NPAQ items | **Spearman’s Correlation**:  All individual NPAQ items were significant (p <0.05) and ranged from r=0.30 to 0.66. Outdoor play preferences had the lowest correlation r= 0.30.  **Intraclass correlation coefficient:**  ICC= 0.70 (95% CI = 0.56, 0.80, p <0.01).  **Cohen’s Kappa:**  κ =0.39 (95% CI = 0.22, 0.56).  Individual items ranged from κ =0.21 to 0.48.  Outdoor play preference had lowest kappa score κ =0.21. |
|  | TV viewing |  | TV viewing | **Intraclass correlation coefficient:**  ICC=0.70(95% CI=0.55, 0.80 p <0.01).  **Cohen’s Kappa:**  κ = 0.53 (95% CI= 0.35, 0.74). |
| Manios et al. (1998) [121];  Greece;  N=39; 6 years;  17 male, 22 female | Proxy report measures -  3 Day Leisure Time Report –parental reported | 2 weeks | Time spent in:  Sedentary  LPA  MVPA | **Spearman’s Correlation:**  r=0.53 (p=0.002)  r=0.27 (p>0.05).  r=0.64 (p<0.001). |
|  | Teacher activity rating |  | Time spent in MVPA | r =0.84 (p<0.001). |
| González‐Gil et al. (2014) [135];  Belgium, Bulgaria, Germany, Greece, Poland and Spain;  n=93; 3.5-5.5 years;  sex not reported  29 children attended kindergartens from the low SEP, 26 from middle, 38 from high. | ‘Toybox’ Primary Caregivers Questionnaire on Lifestyle Behaviours | 2 weeks | PA  SB | **Intaclass correlation coefficient**:  ICC range = 0.304–0.966 (25.9% of questions excellent reliability; 22.2% good; 40.7% moderate; 11.1% poor)  ICC range = 0.058–0.959 (10.5% excellent; 44.7% good; 31.6% moderate; 13.2% poor) |
| **PA (n=2)** | | | | |
| Corder et al. (2009) [68];  UK;  n=20; 4-5 years;  12 male, 8 female | Children’s physical activity questionnaire (CPAQ) | 7 days | MVPA  PAEE | **Intaclass correlation coefficient:**  ICC = 0.39 (p<0.05).  ICC =0.25 |
| Telford et al. (2004) [127];  Australia;  n=58; 5-6 years, mean age 5.3 years;  37 male, 21 female | Children’s Leisure Activities Study Survey (CLASS) | 2 weeks | Frequency (Q1+Q2)  Duration (Q1+Q2) | **Intaclass correlation coefficient:**  ICC=0.83 (p<0.001)  ICC=0.76 (p<0.001). |
| **SB (n=1)** | | | | |
| Mendoza et al. (2013) [128];  USA;  N=80; 3-5 years;  sex not reported  100% Latino or Hispanic.  Children recruited from Head Start Centers | TV Diary | 3-4 weeks | Minutes per day of TV and video viewing | **Intaclass correlation coefficient:**  ICC=0.82 (p<0.001) |

**Intra-instrument reliability of measurement tools (n=1)**

| **Study details** | **Methods** | | **Units of Measure** | **Results** |
| --- | --- | --- | --- | --- |
|  | **Measurement tool(s) examined**  *Placement* | **Study protocol**  *(lab/free living)* |  |  |
| **Accelerometers (n=1)** | | | | |
| **PA (n=1)** | | | | |
| Sharp et al. (2017) [91];  North Wales, UK;  n=56; 3-4 years, mean age 3.7 years;  27 male, 29 female | **Fitbit Zip**  *Both right hip* | Two Fitbit Zip monitors worn simultaneously on the right hip during a 5 minute structured walking task in the nursery *(lab- structured activity protocol)* | Step counts | **Intraclass correlation coefficient:**  ICC between the 2 Fitbits =0.91 (95% CI= 0.85, 0.95) |

**Abbreviations**: PA=physical activity; SB=sedentary behaviour; ICC= Intraclass correlation coefficient; k=kappa; RT3=; MVPA=moderate to vigorous physical activity; LPA= light physical activity; PAEE= physical activity energy expenditure; PRE-PAQ=Pre School Physical Activity Questionnaire; NPAQ= Netherland’s Physical Activity Questionnaire; CPAQ= Children’s physical activity questionnaire; CLASS= Children’s Leisure Activities Study Survey; TV=television; SEP=socioeconomic profile; UK=United Kingdom; USA=United States of America
