# Supplementary material for: A systematic review of the validity, reliability, and feasibility of measurement tools used to assess the physical activity and sedentary behaviour of pre-school aged children
Source: Int J Behav Nutr Phys Act. 2021 Nov 4;18:141. doi: 10.1186/s12966-021-01132-9 (PMC8567581; doi:10.1186/s12966-021-01132-9)
Supplement: Supplementary file 9 — Additional file 9. Study details of feasibility evidence. [file 12966_2021_1132_MOESM9_ESM.docx]

**Additional file 9: Study details of studies with an element of feasibility of measurement tools (n=**41**)**

| **Study details** | Methods | | | **Results** |
| --- | --- | --- | --- | --- |
|  | **Measurement Tool(s)**  *Placement* | **Aspect of feasibility** | **Study protocol** (free living/lab) |  |
| **Calorimetry (n=5)** | | | | |
| **PA and SB (n=4)** | | | | |
| Janssen et al. (2013c) [131];  Australia, mean age 5.3 years;  n=40; 4-6 years;  22 male, 18 female | **WRC** | Practical utility of WRC, assessed by compliance with the graded activity protocol, refusal to participate and data loss. | **150 minute room calorimeter protocol**  *(laboratory based)* | 74% of those eligible agreed to participate.  5% missing data due to calorimeter malfunction (n=2) and 25% did not comply with full protocol (n=10). |
| Oortwjin et al. (2009) [132];  Australia;  N=5; mean age 5.2 years;  3 male, 2 female. | **WRC** | Acceptability, assessed by adherence to study protocol | **150 minute room calorimeter protocol**  *(laboratory based)* | 80% (n=4/5) children completed the protocol  All able to follow instructions, none felt anxious, claustrophobic, or terminated the study early. |
| Janssen et al. (2015) [62];  Australia;  N=40, 4-6 years;  22 male, 18 female | **WRC** | Reasons for drop out/exclusion | **150 minute room calorimeter protocol**  *(laboratory based)* | 5% exclusion due to calorimeter malfunction (n=2) |
| Janssen et al. (2013a) [61];  Australia  N=40; 4-6 years, mean age 5.3 years;  22 male, 18 female. | **WRC** | Reasons for drop out/exclusion | **150 minute room calorimeter protocol**  *(laboratory based)* | 14% exclusion due to: illness (n=1), inability to schedule second visit (n=1), refusal to participate in activity protocol (n=2) and calorimeter malfunction (n=2) |
| **PA (n=1)** | | | | |
| Steenbock et al. (2019) [60];  Germany;  n=41; 3yrs – 6.3 years;  22 male, 19 female  100% Caucasian | Indirect calorimetry: **MetaMax3b**  **DLW** | Reasons for drop out/exclusion | **75 minute protocol of both pre-determined and free play activities** *(both free living and structured)* | 34% exclusion due to: withdrawal consent on day of measurement (n-11), illness (n=5), did not fast prior to DLW (n=2), did not complete activity protocol (n=1) and calorimeter malfunction (n=2). |
| **Accelerometers and calorimetry (n=3)** | | | | |
| **PA and SB (n=1)** | | | | |
| Janssen et al. (2014) [65];  Australia;  N=18; 4-6 years, mean age 5.2 years;  9 male, 9 female | Accelerometer:  **ActivPAL**  *Thigh*  **WRC** | Reasons for drop out/exclusion | **150 minute room calorimeter protocol**  *(laboratory based)* | 10% exclusion due to: accelerometer failure (n=2) and calorimeter failure (n=2) |
| **PA (n=2)** | | | | |
| Lopez-Alarcon et al. (2004) [66];  USA;  N=29; 4-6 years;  17 male, 12 female  58% White  42% African American  6 of the children recruited from Head Start Center | Accelerometer-  **Actiwatch (AW16)**  *Right ankle*  **DLW** | Reasons for drop out/exclusion | **7 days of habitual activity** *(free living)* | 7% exclusion due to: accelerometer failure (n=1) and no urine sample collected by parent (n=1) |
| Sijtsma et al. (2012) [67];  Netherlands;  N=30; 3.1-4.1 years;  12 male, 18 female | Accelerometer:  **Direct life triaxal accelerometer (Tracmor_D_)***-*  *Middle of lower back*  **DLW** | Reasons for drop out/exclusion | **3-5 days of habitual activity** *(free living)* | 17% exclusion due to: accelerometer battery problems (n=1) and no valid energy expenditure data (n=4). |
| **Direct observation (n=3)** | | | | |
| **PA and SB (n=3)** | | | | |
| Puhl et al. (1990) [133];  USA;  n=192, 3-4 year old, mean age not reported;  sex not reported | **Children’s Activity Rating Scale (CARS)** | Reactivity of the children to the presence of an observer (observer reported using brief form). | **Children observed 1-4 times a year, for approx. 10-12 hours per day (observers alternating 2 hour shifts)** *(free living)* | 922 reports filed based on the 491 observations-no child reactivity problems reported in 93.3% of the reports.  153 (16.6%) reactive behaviours were reported where observers thought that the child’s behaviour seemed modified for approx. 30 mins or less (38% of reactive reports), 3 hours or less (30%), half or full day (29%), and an undetermined amount of time (3%). |
| Sharma et al. (2011) [111];  USA;  n= 67; 3-6 years, mean age 4.3 years;  27 male, 40 female  34.8% African American, 63.6% Hispanic,  1.5% Other (White/Native American)  Children recruited from Head Start Centres | **System for Observing Fitness Instruction Time for Preschoolers (SOFIT-P)** | Feasibility of method determined by feedback from trained observers | **1 day of protocol training, followed by two days of implementation and coding** (free living) | Observers stated that SOFIT-P was relatively easy to use and feasible for both indoor and outdoor settings. Training was not overwhelming. No teachers/children refused to participate in measurement, reacted negatively or voiced any discomfort or unease with the observation. |
| Klesges et al. (1985) [72];  USA;  n=30; 41-77 months; 10 male, 20 female | **Fargo Activity Timesampling Survey (FATs)– Time sampling vs continuous observation.** | Completion rate | **Child observed for 1 hour during freeplay in nursery setting** *(free living)* | 100% parents contacted agreed to participate, 100% of these completed the protocol and had usable data. |
| **Direct observation and accelerometers (n=3)** | | | | |
| **PA and SB (n=1)** | | | | |
| Alhassan et al. (2017) [76];  USA;  N=33; Mean age 4.4 years; 21 male, 12 female | **Actiwatch (Spectrum)**  *Non dominant wrist*  **Actigraph (GT3X)**  *Waist, centre of lower back*  **Direct observation (OSRAC-P)** | Reasons for drop out/exclusion | **30 minutes of daily activity in pre-school** *(free living)* | 55% exclusion due to: Actiwatch reported as ‘off-wrist’ (n=11- 15%), Actigraph registered continuous zeros (n=15- 20%), participants absent from direct observation (n=15- 20%) |
| **PA (n=1)** | | | | |
| Sharp et al. (2017) [91];  North Wales, UK;  N=56; 3-4 years, mean age 3.7 years;  29 female, 27 male | **FitBit (Zip)**  *Both right hip*  **Direct observation** | Reasons for drop out/exclusion | **5 min structured walking task** *(nursery setting)* | 15% exclusion due to: not performing task as requested (n=5) and technical errors in video recordings (n=5) |
| **SB (n=1)** | | | | |
| Alghaeed et al. (2013) [87];  Scotland, UK;  N=30; Mean age 4.1 years; 10 male, 20 female | **ActivPAL**  *Thigh*  **Direct observation** | Reasons for drop out/exclusion | **1 hour during usual activity in nursery** *(free living)* | 6% exclusion due to not having complete datasets for accelerometer and direct observation (n=2) |
| **HRM (n=1)** | | | | |
| **PA and SB (n=1)** | | | | |
| Bar-Or et al. (1996) [73];  Canada;  n=23; 3-5 years, mean age not reported;  11 female, 12 male | **Polar Vantage XL Monitor**  *Chest and wrist watch* | Acceptability of device (researcher reported) | **Child observed twice for 60-90 mins wearing HRM, researcher evaluated child’s acceptance based on 6 point scale** *(free living).* | 90% of children rated between enthusiastic and agreed. Decrease by 4% in refusal ratings by observation 2. |
| **Accelerometers (n=20)** | | | | |
| **PA and SB (n=13)** | | | | |
| Bacardi-Gascón et al. (2012) [124];  Mexico;  N=35; 3-5 years;  17 male, 18 female | **Actigraph (GT1M)**  *Right hip* | Reasons for drop out/exclusion | **4 days habitual activity- accelerometer used as comparison method against proxy report tool** *(free living)* | 22% exclusion due to not wearing accelerometer for sufficient amount of time (n=10) |
| Dobell et al. (2019) [75];  UK;  N=62; 3-4 years, mean age 3.5 years;  36 males, 30 females (sex of participants prior to exclusion of 4 data points) | **Actigraph (GT3X)**  *Right hip and non-dominant wrist* | Reasons for drop out/exclusion | **60 minutes of activity in pre-school setting** *(free living)* | 6% exclusion due to: accelerometer failure (wrist worn) (n=1) and child withdrawal while wearing accelerometers (n=3) |
| Hands & Larkin (2006) [82];  Australia;  N=23; 5-6 years;  12 male, 11 female | **Actigraph (MTI AM7164)**  *Waist* | Reasons for drop out/exclusion | **Children monitored for 30 minutes over 5 days during free playing in their school setting** *(free living)* | 4% exclusion due to child spending majority of the time on a swing which inflated the accelerometer count (n=1) |
| Sirard et al. (2005) [85];  USA;  N=269; 3-5 years, mean age not reported;  125 male, 144 female  3yr; 37.7% white  4yr; 25.6% white  5yr; 38.7% white | **Actigraph (MTI)**  *Right hip* | Reasons for drop out/exclusion | **Children wore Actigraph for the entire time they were at school for up to 10 consecutive weekdays, direct observation for 1-3 hours of activity in preschool** *(free living)* | 4% exclusion due to incomplete or missing data (n=12) |
| Dwyer et al. (2011) [122];  Australia;  N= 67; 3-5 years;  36 male, 31 female  91% White, 3% Mediterranean, 6% other ethnicity.  9% low SEP, 34% middle,  57% high | **Actigraph (MTI 7164)**  *Right hip* | Reasons for drop out/exclusion | 3 day activity *(free living)* | 36% exclusion due to: not wearing accelerometer for sufficient amount of time (n=38) |
| Wen et al. (2010) [125];  Australia;  n=31 ; 3-5 years;  19 male, 12 female  Children recruited from centers said to vary in SES. | Accelerometer-  **Actigraph (GT1M)**  *Right hip* | Did not complete measures for required period of time. | **7 day accelerometer wearing protocol** *(free living)* | 30% exclusion due less than 4 days accelerometer data (n=13). |
| Martin et al. (2011) [113];  Scotland, UK;  N=23; 3-5 years;  9 male, 14 female | **Actigraph (GT1M and GT3X)**  *Right hip*  **ActivPAL**  *Thigh* | Reasons for drop out/exclusion | **Monitors worn simultaneously for a 7 consecutive days during waking hours, expect during water based activities** *(free living activity)* | 28% exclusion due to : lost/missing or malfunctioning accelerometer (n=2), non-compliance with study protocol (n=6) or illness (n=1) |
| Davies et al. (2012) [88];  UK;  n=20; 3.2-4.9 years, mean age 4.4 years;  6 male,14 female | **ActivPAL**  *Thigh* | Acceptability of devices (parent reported); reasons for missing or invalid data | **Children wore activPAL monitor for up to 7 days. Parents completed 10 item questionnaire to assess acceptability of devices on 5 point Likert scale** *(free living).* | **Acceptability:**  Support for practicality of using activPAL in preschool children. One parent reported that they stopped using the overlying Tegaderm dressing because it was uncomfortable for their child.  **Missing or invalid data:**  206 hours of data loss over the week, equivalent to 10.3 hours of missing data per participant per week. |
| Janssen et al. (2013b) [89];  Australia;  n=38; 4-6 years, mean age 5.3 years;  20 male, 18 female | **ActivPAL**  *Thigh* | Reasons for drop out/exclusion | **150 minute structured activity protocol**  *(laboratory based)* | 5% exclusion due to accelerometer failure (n=2) |
| Vanderloo et al. (2016) [129];  Canada;  N=23; 4-5 years;  12 male, 16 female | **Actical**  *Right hip*  **Actigraph (GT3X+)**  *Right hip* | Reasons for drop out/exclusion | **Both monitors worn simultaneously for 7 consecutive days** *(free living)* | 18% exclusion due to not wearing accelerometer for required amount of time (n=5) |
| Ettienne et al. (2016) [86];  USA;  n=30; Mean age =3.5 years;  17 male, 13 female  46% Native Hawaiian, 14% Other Pacific Islander-  All of mixed ethnicities.  Children recruited from Head Start sites | **Actical**  *Non dominant wrist* | Reasons for drop out/exclusion | **7 days habitual activity** *(free living)* | 10% exclusion due to not completing both phases of work (39/49) and/or having at least one day of data deleted due to the total number of mins of SB being greater than 1300 minutes per day (23/49) |
| Kelly et al. (2004) [83];  UK;  n=78; 3-4 year olds, mean age 3.5 years;  30 male, 48 female | **Actiwatch (AW16)**  *Right hip*  **Actigraph (CSA/MTI)**  *Right hip* | Reasons for drop out/exclusion | **39-45 minutes structured play class** *(usual activity, reflective of free living)* | 7% exclusion due to technical failures and lack of cooperation (n=6) |
| Shin (2015) [114];  USA;  N=19; 3-5 years;  10 male, 9 female | **BFF**  *Left and right wrist, back, waist, chest, ankle, upper arm*  **Actigraph (GT3X/GT3X+)** *Waist and Right Wrist* | Acceptability of device | **Children followed structured activity protocol at the pre-school under researchers instruction – participant feedback used to determine feasibility** *(usual activity, not free living)* | **Acceptability**:  According to their feedback, participants agreed to accept the size and features of devices in use. However, the comfort level of the devices was in question- participants sometimes requested to stop testing when they felt uncomfortable wearing devices on their chests, especially when they slipped. Many participants strongly opposed attaching the devices on their chests directly, but accepted them attached over clothes.  (Also reported low response rate – 36% as part of feasibility aspect) |
| **PA (n=4)** | | | | |
| Nyström et al. (2017) [64];  Sweden;  N=40; 5.2- 5.7 years, mean age 5.5 years;  22 male, 18 female | **Actigraph (wGT3X-BT)**  *Non-dominant wrist*  *1 sec epoch*  *No cut points reported for PA*  *Non-wear time:* *Determined using raw acceleration data. SD of each axis calculated over a 30-min period. If SD of acceleration of any two axes less than 0.002 g for the same window= non-wear time.*    *Valid day based on wear time:*  *When the awake wearing time (non-sleeping data) was ≥600 minutes*  *Number of days: Minimum of 3 days of valid data.* | Compliance with study protocol | **7 days activity** *(free living)* | 100% compliance with activity protocol and accelerometer wearing; Thirty-four children (85%) wore the ActiGraph for 7 days,4 children (10%) for 6 days, 1 child (2.5%) for 5 days and 1 child (2.5%) for 4 days |
| Corder et al. (2009) [68];  UK;  N=27; 4-5 years;  17 male, 10 female | **Actigraph (MTI 7164)** | Reasons for drop out/exclusion | **Questionnaire for past 7 days, remaining protocol for 6-10 days assessing habitual activity** *(free living)* | 7% participants had accelerometer malfunction (n=2) |
| Curtis Ellison et al. (1992) [134];  USA;  N=100; 3-5 years, mean age and sex not reported  Predominantly white, middle class families. | **Caltrac accelerometer**  *Right hip* | Reasons for lack of usable data; Cost of devices (including replacement for malfunctions*)* | **Children asked to wear accelerometer for 5 consecutive days, at 2 time points, 6 months apart** *(free living).* | 82% of schedule days were available, all families provided usable data.  Looking at a random sub-set of 500 reports, it was identified that on days when data were not obtained:  52% of the time it was due to the parent forgetting to attach/refusal from child to wear the device in the morning; 28% of the time information parent record was insufficient in regards to when monitor was attached/removed; 20% of the time it was due to instrument malfunction.  With overall costs for devices, batteries and repairs, the average cost per day of successful monitoring = £1.31. |
| Chen et al. (2002) [120];  Japan;  n=21; 3-4 years;  12 male, 9 female | Accelerometer-  **Actiwatch-L**  **Caloriecounter** | Reasons for drop out/exclusion | **3 days of accelerometer wearing** *(free living activity)* | 8% exclusion due to measurement failure from instruments (n=2) |
| **SB (n=3)** | | | | |
| De Decker et al. (2013) [78];  Belgium;  n=52; mean age 5.5 years; 26 male, 26 female | **ActivPAL**  *Thigh*  **Actigraph (GT1M)**  *Right hip* | Practical utility of the accelerometers (parent reported); Reasons for drop out/exclusion*.* | **Monitors worn for at least 3 days simultaneously, direct observation for 1 hour during pre-school hours. Parents asked to indicate on 5 point scale whether their child found it pleasant or unpleasant to wear the devices** *(free living)* | **Practical utility**:  71% (n=28) parents indicated on a 5 point scale that their child found it pleasant to wear both monitors. 4 parents indicated that their child found it very unpleasant to wear the ActivPAL during measurements; 38% (n=15) reported that their child had some skin irritation to on the thigh due to wearing the waterproof ActivPAL for 5 consecutive days.  Reasons for exclusion/drop out:  15% children did not wear ActivPAL (n=8), 13% did not have adequate data from both devices for at least 3 days (n=7). |
| Van Cauwenberghe et al. (2012) [130];  New Zealand;  N=49; 3-4 years;  22 male, 19 female | **Actical**  *Waist, above the right iliac crest*  **ActivPAL**  *Thigh* | Acceptability of devices (researcher reported); Reasons for drop out/exclusion | **Monitors worn simultaneously during 1 day at nursery. Researchers coded children’s acceptability to wearing monitors on 5 point scale** *(free living)* | **Acceptability**:  81.6% of children had high or very high acceptability of wearing both monitors, very low acceptability was not reported for any children.  **Reasons for drop out/exclusion**:  36% excluded data due to: did not attend the centre on the day of measurement (n=8); did not assent to wear one or both monitors (n = 7); technical failure when downloading the raw activPAL data (n = 5); monitors not worn simultaneously (n= 3): monitors removed during the measurement period (n = 2); and monitors worn for less than 3 h (n = 2). |
| Mendoza et al. (2013) [128];  USA;  N=96; 3-5 years;  41 female, 53 male, 2 missing  100% Latino or Hispanic.  Children recruited from Head Start Centers | Accelerometer- **Actigraph (GT1M)**  *Hip* | Compliance; Retention rate | **Measurement tools administered for 7 days at 2 time periods** *(free living)* | 100% compliance at time 1 and 82.3% at time 2.  Retention rate- 85.4% |
| **Pedometers (n=3)** | | | | |
| **PA (n=3)** | | | | |
| Murray (2009) [99];  USA;  N=75; 3-5 years;  21 male, 44 female  34.3% African American;  64.4% Hispanic;  1.4% Native American  Children recruited from Head Start Centres | **MVP 4 Walk4Life Digital Pedometer**  *Right or left hip* | Process evaluation from data collectors and teacher input, to determine feasibility. | **Children were observed in pre-school whilst wearing pedometer at 2 time points**  *(free living).* | Reports of inability to wear the pedometer for the full day – failing off, restroom breaks, loose pants, nap time, play time. At pre-test, measurement 1 – 45% (n=34) of the children did not achieve sufficient wear time and at post-test, measurement 1 49% (n=37) did not achieve sufficient wear time.  Lacked feasibility due to logistical barriers in design. |
| Chow & Au (2009) [126];  Hong Kong  n=23; 5-6 years;  12 male, 11 female  Majority of the children from lower to lower-middle families. | **Yamax Digiwalker (SW-700)**  Right side of w*aist* | Reasons for exclusion/drop out | **Pedometer worn for 4 days, as comparison method for proxy report** *(free living)* | 23% (n=7) missing data as parents forgot to complete pedometer record. |
| Louie & Chan (2003) [95];  Hong Kong;  N=145; 3-5 years, mean age 4.2 years;  84 male, 61 female | **Yamax Digiwalker (SW-200)**  *Right hip (some invited to wear on both right and left hip)* | Reasons for exclusion/drop out | **25 minute physical activity class in normal school day** *(free living)* | 2% exclusion of data due to pedometer battery problems (n=3) |
| **Accelerometers and pedometers (n=3)** | | | | |
| **PA and SB (n=1)** | | | | |
| Pagels et al. (2011) [116];  Sweden/USA;  N=55; 3.4-5.7 years;  28 male, 27 female | **Accelerometer-**  **Actigraph (GT1M)**  *Waist belt*  **Pedometer –**  **Yamax SW-200**  *Right side of waist* | Reasons for exclusion/drop out | **Children wore monitors simultaneously for 5 days during nursery time** *(free living)* | 5% (n=3) excluded due to incomplete data. |
| **PA(n=2)** | | | | |
| Cardon & De Bourdeaudhuij (2007) [115];  Belgium;  N=76; 4-5.9 years; 37 male, 39 female  55% from high SEP;  45% from low SEP | **Accelerometer- Actigraph (MTI AM 7164)**  *Right hip*  **Pedometer- Yamax Digiwalker (SW-200)**  *Right hip* | Receptivity to measurement tools (parent reported);  Reasons for drop out/exclusion | **Monitors worn simultaneously for waking hours up to 5 days. Parents asked to report child’s receptivity to wearing the instruments on 5 point scale** *(free living).* | **Receptivity/Acceptability**:  83% of children found it very pleasant (59,46%) or pleasant (50 children, 38%) to wear accelerometer or accelerometer and pedometer; 8% (11 children) found it not pleasant or not unpleasant, 2% (2 children) found It unpleasant  Receptivity of wearing the instruments was higher in children concurrently wearing the accelerometer and the pedometer (1.7 ± 0.8) than in children wearing only the pedometer  (1.4 ± 0.5; t = 2.6, p = 0.01).  **Reasons for drop out/exclusion:**  5% excluded: Incomplete data due to not wearing pedometer more than 1hr on 1 or more days (n=7), pedometer lost (n=1), monitor damaged (n=1).  15% excluded due to satisfactory measurements not obtained from accelerometer (n=13) |
| De Craemer et al. (2015) [118];  Belgium;  N=41; 4-6 years;  21 male, 20 female | **Actigraph (GT1M)**  *Left hip*  **Pedometer** *-*  **Omron Walking Style Pro (HJ-720IT-E2)**  *Right hip* | Reasons for exclusion/drop out | **Children wore monitors simultaneously for at least 1 day** *(free living)* | 44% missing data (n=23), as children did not have valid data for both measurement devices |

**Abbreviations**: WRC= whole room calorimetry; DLW= doubly labelled water; CARS= Children’s Activity Rating Scale; SOFIT-P= System for Observing Fitness Instruction Time for Preschoolers; OSRAC-P= Observation System for Recording Physical Activity in Children- Preschool; HRM= heart rate monitor; USA=United States of America; UK=United Kingdom
