# Supplementary material for: A systematic review of the validity, reliability, and feasibility of measurement tools used to assess the physical activity and sedentary behaviour of pre-school aged children
Source: Int J Behav Nutr Phys Act. 2021 Nov 4;18:141. doi: 10.1186/s12966-021-01132-9 (PMC8567581; doi:10.1186/s12966-021-01132-9)
Supplement: Supplementary file 10 — Additional file 10. Source of funding for each study. [file 12966_2021_1132_MOESM10_ESM.docx]

**Additional file 10: Source of funding for each study**

| Study details | Source of funding |
| --- | --- |
| Adolph et al. (2012) [57];  USA;  n=64; 3-5 years;  37 male, 27 female  50% White, 27% Black, 22% Hispanic, 2% Asian | Federal funds from the USDA/ARS under cooperative agreement (6250-51000-037) and NIH grant (US National Institutes of Health R01 DK074387). |
| Algaheed et al. (2013) [87];  Scotland, UK;  N=30; Mean age 4.1 years; 10 male, 20 female | Study funded by MD Bench fees of first author. |
| Alhassan et al. (2017) [76];  USA;  N=33; Mean age 4.4 years; 21 male, 12 female | The study was supported, in part, by NIH (R01-HL111695). |
| Bacardi-Gascon et al. (2012) [124];  Mexico;  N=35; 3-5 years;  17 male, 18 female | No funding source reported. |
| Bar Or et al. (1996) [73];  Canada;  n=27; 3-5 years;  18 male, 9 female- Study 1 | No funding source reported. |
| Bar Or et al. (1996) [73];  Canada;  n=23; 3-5 years;  11 female, 12 male- Study 2 | No funding source reported. |
| Bikchu (2014) [117];  Hong Kong;  N=143; 4-5 years;  80 male, 93 female | No funding source reported. |
| Byun (2018a) [90];  USA;  n=28; 3-5 years;  15 male, 13 female | No funding source reported. |
| Byun et al. (2018b) [112];  USA;  n=27; 3-5 years;  16 male, 11 female | No funding source reported. |
| Cardon and De Bourheaudhiuj (2007) [115];  Belgium;  N=76; 4-5.9 years; 37 male, 39 female  55% from high SEP;  45% from low SEP | No funding source reported. |
| Chen et al. (2002) [120];  Japan;  n=21; 3-4 years;  12 male, 9 female | No funding source reported. |
| Chow et al. (2009) [126]  Hong Kong  n=23; 5-6 years;  12 male, 11 female | No funding source reported. |
| Corder et al. (2009) [68]  UK  N=27; 4-5 years;  17 male, 10 female | No funding source reported. Authors supported by the Medical Research Council. |
| Curtis Ellison et al. (1992) [134];  USA;  N=100; 3-5 years;  sex not reported | Study supported by grant (HL-35653) from National Heart, Lung, and Blood Institute and a Grant from the American Heart Association, Massachusetts Affiliate. |
| Davies et al. (2012) [88];  UK;  n=32; 3.1-4.9 years;  11 male, 21 female | No funding source received.  One of the authors is a co-inventor of the accelerometer, but was not involved in data collection or statistical analysis of the results. |
| Davies et al. (2012) [88];  UK;  n=20; 3.2-4.9 years;  6 male,14 female | No funding source received.  One of the authors is a co-inventor of the accelerometer, but was not involved in data collection or statistical analysis of the results. |
| De Craemer et al. (2015) [118]  Belgium;  N=41; 4-6 years;  21 male, 20 female | No funding source reported. |
| De Decker et al. (2013) [78];  Belgium;  n=52; mean age 5.5 years;  26 male, 26 female | No funding source reported. |
| Djafarian et al. (2013) [94];  Scotland, UK;  N=42; 3-5 years;  22 male, 20 female | No funding source reported. |
| Dobell et al. (2019) [75];  UK;  N=62; 3-4 years, mean age 3.5 years;  36 males, 30 females (sex of participants prior to exclusion of 4 data points) | No funding source reported. |
| Dwyer et al. (2011) [122];  Australia;  N= 67; 3-5 years;  35 male, 32 female  91% White, 3% Mediterranean, 6% other ethnicity.  9% low SEP, 34% middle,  57% high | Coles Community Development Fund Grant. Lead author was supported by NHMRC Postgrad Allied Health Research Scholarship. |
| Ettienne et al. (2016) [86];  USA;  n=30; Mean age =3.5 years;  17 male, 13 female  46% Native Hawaiian, 14% Other Pacific Islander-  All of mixed ethnicities.  Children recruited from Head Start sites | Part of the Children’s Healthy Living (CHL) Program. CHL program funded by the Agriculture and Food research initiative grant (2011-68001-30335), from the USDA National Institute of Food and Agricultural Science Enhancement Coordinated Agricultural Program. |
| Fairweather et al. (1999) [81];  Scotland, UK;  n=11; 3.7 years;  3 male, 8 female – Study 1 | No funding source reported. |
| Fairweather et al. (1999) [81];  Scotland, UK;  n=10;4 years; 1 male, 9 female- Study 2 | No funding source reported. |
| Finn & Specker (2000) [93];  USA;  n=40; 3-4 years;  16 male, 24 female  95% Caucasian | NIH grant (R01 AR45310) |
| Fotini et al. (2015) [136];  Greece;  N=45; mean age 66.35 months; sex not reported | Funding from the EU Social Fund and Greek National Funds through Operational Program 'Education and Lifelong learning' of the National Strategic Reference Framework. |
| Gonzalez-Gil et al. (2014) [135];  Belgium, Bulgaria, Germany, Greece, Poland and Spain;  n=93; 3.5-5.5 years;  sex not reported  29 children attended kindergartens from the low SEP, 26 from middle, 38 from high. | Part of a larger study (ToyBox)- funded by the Seventh Framework Programme (CORDIS FP7) of the European Commission Grant. |
| Hands et al. (2006) [82];  Australia;  N=23; 5-6 years;  12 male, 11 female | No funding source reported. |
| Hislop et al.(2012a) [79];  Scotland, UK;  N=31; 3-5 years; 15 male, 16 female | Funding provided by Chartered Society of Physiotherapy’s physiotherapy research foundation grant. |
| Hislop et al. (2012b) [80];  Scotland, UK;  N=31; 3-5 years;  15 males, 16 females | Funding provided by Chartered Society of Physiotherapy’s physiotherapy research foundation grant. |
| Hislop et al. (2016) [74];  Scotland, UK;  N=32; 3-5 years;  21 male, 11 female | No funding source reported. |
| Janssen et al. (2013a) [61];  Australia  N=40; 5.3 years;  22 male, 18 female | National Heart Foundation of Australia (GIA09S4441). Authors supported by National heart foundation of Australia- postdoctoral research fellowship (PH 09S 4603) and national heart foundation of Australia career development fellowship (CR 11S 6099). |
| Janssen et al. (2013b) [89];  Australia;  n=38; 4-6 years;  20 male, 18 female | National Heart Foundation of Australia (GIA09S4441). |
| Janssen et al. (2013c) [131];  Australia;  n=40; 4-6 years;  22 male, 18 female | National Heart Foundation of Australia (GIA09S4441). Authors supported by National heart foundation of Australia- postdoctoral research fellowship (PH 09S 4603) and national heart foundation of Australia career development fellowship (CR 11S 6099). |
| Janssen et al. (2014) [65];  Australia;  N=18; 4-6 years;  9 male, 9 female | National Heart Foundation of Australia (GIA09S4441). |
| Janssen et al. (2015) [62];  Australia;  N=40, 4-6 years;  22 male, 18 female | National Heart Foundation of Australia (GIA09S4441). |
| Janz et al. (2005) [123];  USA;  N=204; 4-7 years;  91 male, 113 female  96% Caucasian  Almost all had families of relatively high socioeconomic status; only 10% listed a family income below 20,000 dollars. | National Institute of Dental and Craniofacial Research (EO1-DE12101; RO1-DE09551) and General Clinical Research Centers Program, National Center for Research Resources (RR00059). |
| Kahan et al (2013) [77] –  Study 1:  USA; n=57; 4-5 years;  25 male, 32 female  70.2% White, 12/3% Asian/Pacific Islander, 10.5% Black/African American,  7% Hispanic/Latino. | Partially funded by the California Association of Health, Physical Education, Recreation & Dance Foundation for Promotion of Health Lifestyles and Active Living research, a program of the Robert Wood Johnson foundation. |
| Kahan et al (2013) [77]-  Study 2:  USA; n=12; 4-5 years;  3 male, 9 female | Partially funded by the California Association of Health, Physical Education, Recreation & Dance Foundation for Promotion of Health Lifestyles and Active Living research, a program of the Robert Wood Johnson foundation. |
| Kelly et al. (2004) [83];  UK;  n=78; 3-4 year olds; 30 male, 48 female | Study funded by Sport Aiding Medical Research for Kids (SPARKS). |
| Klesges et al. (1985) [72];  USA;  n=30; 41-77 months; 10 male, 20 female | National Institute of child health and human development grant (R23-HD17796). |
| Larson et al., 2011 [110];  USA;  N=4; 3-5 years;  2 male, 2 female | No funding source reported. |
| Lee et al. (2014) [119];  Korea;  N=131; 3-6 years, sex not reported | No funding source reported. |
| Liggett et al. (2012) [92];  New Zealand;  N=14; 3-4 years old;  7 male, 7 female. | Research lead by Southland District Health Board, in partnership with Sport Southland. Co funded by Ministry of Health through the HEHA Innovations Fund. |
| Lopez Alarcon et al. (2004) [66];  USA;  N=29; 4-6 years;  17 male, 12 female  58% White  42% African American  6 of the children recruited from Head Start Center | Work supported by: NIH Grant (RO1 DK51684) ; General Clinical Research Center Grant (M01- RR00032); Clinical Nutrition Research Unit Grant (P30-DK56336).  Manufacturer provided the accelerometers.  Costs of article defrayed, in part, by the payment of page charges. The article must therefore be marked as 'advertisement' in accordance with 18 U.S.C Section 1734 to indicate the fact. |
| Louie and Chan (2003) [95];  Hong Kong;  N=145; 3-5 years;  84 male, 61 female | No funding source reported. |
| Manios et al. (1998) [121];  Greece;  N=39; 6 years,  17 male, 22 female | Funded by the EU, the Europe Against Cancer Program, and the Mediterranean Integrated Program of Crete, General Secretary of the Periphery of Crete. |
| Martin et al. (2011) [113];  Scotland, UK;  N=23; 3-5 years;  9 male, 14 female | No funding source reported. |
| McKee et all. (2005) [96];  Northern Ireland, UK;  N=30; 3-4 years;  13 male, 17 female | No funding source reported. |
| Mendoza et al. (2013) [128];  USA;  N=96; 3-5 years;  53 male, 41 female, sex of 2 participants not reported  100% Latino or Hispanic.  Children recruited from Head Start Centers | US National Cancer Institute, National Institutes of Health K07CA131178 and the US Department of Agriculture Cooperative Agreement (6250-51000-053). |
| Murray (2009) [99];  USA;  N=75; 3-5 years;  31 male, 44 female  34.3% African American,  64.4% Hispanic,  1.4% Native American  Children recruited from Head Start Centres | This study was conducted as part of the Coordinated Approach to Child Health in Underserved  Populations (CATCH UP) obesity intervention that was be pilot tested in two Head Start  centers over the 2008-2009 school year. Funding for CATCH UP was provided by the Michael & Susan Dell Center for Advancement of Healthy Living and the University of Texas, School of Public Health. |
| Nishikido et al. (1982) [98]  Japan;  n=49; 5-6 years;  25 male, 24 female | No funding source reported. |
| Noland et al. (1990) [100];  USA;  n=21; 3.8-5.6 years  11 male, 10 female  90% White, 10% Black  Families were primarily middle and upper class. | Supported, in part, by NIH (HL-35100). |
| Nyström et al. (2017) [64];  Sweden;  N=40; 5.2- 5.7 years;  22 male, 18 female | Authors supported by: the Swedish Nutrition Foundation; Swedish Research Council (project no. 2012–2883), the Swedish Research Council for Health, Working Life and Welfare (2012-0906), Bo and Vera Axson Johnsons Foundation and Karolinska Institutet; and Henning and Johan Throne-Holst Foundation. |
| Oliver et al. (2007) [97];  New Zealand;  N=13; 3-4.8 years;  7 male, 6 female | First author supported by Tertiary Education Commission Top achiever doctoral scholarship. Part of the study was funded by Sport and Recreation New Zealand as a component of active movement scoping exercise and programme evaluation. |
| Oortwjin et al. (2009) [132];  Australia;  N=5; mean age 5.2 years;  3 male and 2 female. | No funding source reported. |
| Pagels et al. (2011) [116];  Sweden/USA;  N=55; 3.4-5.7 years; 28 male, 27 female | No funding source reported. |
| Pate et al. (2006) [58];  USA;  N=29; 3-5 years;  13 male, 16 female  55.2% African American,  44.8% White | Project funded by an International Life Sciences Institute Research Foundation grant and NIH Grant (R01 HD043125).  Costs of article defrayed, in part, by the payment of page charges. The article must therefore be marked as 'advertisement' in accordance with 18 U.S.C Section 1734 to indicate the fact. |
| Pfeiffer et al. (2006) [63];  USA;  n=18; 3.4-5.7 years;  7 male, 11 female  89% African American | Project funded by ILSI Research Foundation Grant and NIH (RO1 HD043125).  Some accelerometers provided by manufacturer. |
| Puhl et al. (1990) [133];  USA;  n=192, 3-4 year olds;  sex not reported | Research supported by National heart, lung, and blood institute (#5R01 HL35131 03). |
| Reilly et al. (2003) [84];  Scotland, UK;  N=52; 3-4 years; 21 male, 31 female | Study funded by Sport Aiding Medical Research for Kids (SPARKS) AND Rank prize funds. |
| Reilly et al. (2006) [59];  Scotland, UK;  N=85; 3-6 years;  51 male, 34 female | Study funded by Sport Aiding Medical Research for Kids (SPARKS). |
| Saris and Binkhorst (1977) [137];  Netherlands;  N=4; 4-6 years (exact ages not reported); sex not reported | No funding source reported. |
| Sharma et al. (2011) [111];  USA;  n= 67; 3-6 years;  27 male, 40 female- Study 1  34.8% African American, 63.6% Hispanic,  1.5% Other (White/Native American)  Children recruited from Head Start Centres | Research supported by grants from the Ministry of Health and Welfare (H10-Child-020) and Toyama Medical Association. |
| Sharma et al. (2011) [111];  USA;  n=27; 3-6 years;  11 male, 16 female – Study 2  23.1% African American, 65.4% Hispanic, 7.7% Other (White/Native American)  Children recruited from Head Start Centres | Research supported by grants from the Ministry of Health and Welfare (H10-Child-020) and Toyama Medical Association. |
| Sharp et al. (2017) [91]  North Wales, UK;  N=56; 3-4 years;  29 female, 27 male | School of Psychology, Bangor University supported the study financially. |
| Shin (2015) [114];  USA;  N=19; 3-5 years;  10 male, 9 female | No funding source reported. |
| Sijtsma et al. (2013) [67];  Netherlands;  N=30; 3.1-4.1 years;  12 male, 18 female | No funding source reported.  Manufacturer provided the accelerometers. |
| Sirard et al. (2005) [85];- Study 1  USA;  N=16; 3-5 years; 10 male, 6 female-  3yr100% white, 4yr 50% white, 5yr 33.3% white | Supported by grant from Gerber Products Inc. |
| Sirard et al. (2005) [85];- Study 2  USA;  N=269; 3-5 years;  125 male, 144 female  3yr; 37.7% white, 4yr; 25.6% white, 5yr; 38.7% white | Supported by grant from Gerber Products Inc. |
| Steenbock et al. (2019) [60];  Germany;  n=41; 3yrs – 6.3 years;  22 male, 19 female  100% Caucasian | Study supported by internal innovations grant of the Leibniz Institute for Prevention Research and Epidemiology. |
| Telford et al. (2004) [127];  Australia;  n=58; 5-6 years;  37 male, 21 female | Financial Markets Foundation for Children; authors funded by: Australian Postgraduate Award PhD Scholarship; public health fellowship from Victorian Health Promotion Foundation; National Health and Medical research council and National Heart Foundation Career Development Award in Population Health. |
| Van Cauwenberghe et al. (2012) [130];  New Zealand;  N=49; 3-4 years;  22 male, 27 female | Authors funded by: PhD scholarship from Research Foundation- Flanders (FWO B/10525/01); and Auckland University of Technology Faculty of Health and Environmental Sciences Summer Studentship Grant. |
| Vanderloo et al. (2016) [129];  Canada;  N=23; 4-5 years;  12 male, 16 female (sex sample size prior to exclusion of participants) | Study conducted in conjunction with Health Outcomes and Physical activity in Preschoolers (HOPP) study. HOPP study funded by Canadian Institute of Health Research (CIHR) Operating Grant (#MOP 102560). Authors supported by CIHR New Investigator Salary Award and CIHR Doctoral Research Award. |
| Wen et al. (2010) [125];  Australia;  n=31 ; 3-5 years;  19 male, 12 female  Children recruited from centers said to vary in SEP. | No funding source reported. |
